# Synthesis of Novel Bisindolylmethane Schiff bases and Their Antibacterial Activity

**DOI:** 10.3390/molecules190811722

**Published:** 2014-08-06

**Authors:** Syahrul Imran, Muhammad Taha, Nor Hadiani Ismail, Khalid Mohammed Khan, Farzana Naz, Memona Hussain, Saima Tauseef

**Affiliations:** 1 Atta-ur-Rahman Institute for Natural Product Discovery, Universiti Teknologi MARA, Puncak Alam Campus 42300, Malaysia; 2 Faculty of Applied Science, Universiti Teknologi MARA, Shah Alam 40450, Selangor D.E., Malaysia; 3 H. E. J. Research Institute of Chemistry, International Center for Chemical and Biological Sciences, University of Karachi, Karachi-75270, Pakistan; 4 Department of Pharmaceutical Chemistry, Faculty of Pharmacy, Federal Urdu University of Arts, Science and Technology, Karachi-75370, Pakistan; 5 Department of Microbiology, Federal Urdu University of Arts, Sciences and Technology, Gulshan-e-Iqbal Campus, Karachi-75370, Pakistan

**Keywords:** indole, bisindolylmethane, bisindole Schiff base, antibacterial, sodium borohydride, nickel acetate

## Abstract

In an effort to develop new antibacterial drugs, some novel bisindolylmethane derivatives containing Schiff base moieties were prepared and screened for their antibacterial activity. The synthesis of the bisindolylmethane Schiff base derivatives **3**–**26** was carried out in three steps. First, the nitro group of 3,3'-((4-nitrophenyl)-methylene)bis(1*H*-indole) (**1**) was reduced to give the amino substituted bisindolylmethane **2** without affecting the unsaturation of the bisindolylmethane moiety using nickel boride *in situ* generated. Reduction of compound **1** using various catalysts showed that combination of sodium borohydride and nickel acetate provides the highest yield for compound **2**. Bisindolylmethane Schiff base derivatives were synthesized by coupling various benzaldehydes with amino substituted bisindolylmethane **2**. All synthesized compounds were characterized by various spectroscopic methods. The bisindolylmethane Schiff base derivatives were evaluated against selected Gram-positive and Gram-negative bacterial strains. Derivatives having halogen and nitro substituent display weak to moderate antibacterial activity against *Salmonella typhi*, *S. paratyphi* A and *S. paratyphi* B.

## 1. Introduction

As the rate of resistance to current antibacterial therapy has increased alarmingly, the search for new selective [1] and nontoxic [2] antibacterial agents has become an important area of investigation in medicinal chemistry. Resistance of pathogenic bacteria to drugs is constantly emerging, even though various classes of antibacterial agents are available. Chemical diversity and various mechanisms of action make it difficult to find a definite way of identifying new drugs. This has not only made treating infectious diseases more problematic, but has also resulted in reappearance of many diseases which were thought to be under control such as malaria. To overcome this severe medical issue, discovery of new types of antibacterial agents or improvement for the bioactivity of the existing drugs is an important task [3]. Hence, an increasing number of current research is oriented towards development of new antibacterial agents using novel lead compounds to overcome the problem of bacterial resistance against available drugs.

Synthesis, characterization and structural activity relationship of Schiff bases have been studied worldwide as it is proven that C=N linkage in Schiff bases is an essential feature for bioactivity [4]. Schiff bases have been reported to possess noteworthy antibacterial [5], antifungal [6], anticancer [7], urease inhibition [8], antioxidant [9,10,11,12,13,14] and antiglycation [15,16,17] activities.

Because of their vast pharmacological activities, indole and their derivatives are an important class of heterocycles and bioactive intermediates in the pharmaceutical industry and organic synthesis [18]. Chemists are continuously developing simple and efficient synthetic protocols to synthesize indoles and their derivatives due to their excellent biological activities [19]. The indole ring has become an important structural requirement in many pharmaceutical drugs because of the structural diversity of biologically active indoles and their derivatives [20]. Indole is a valuable compound which has become prominent in medicinal chemistry because of its various biological activities [21] such as anticonvulsant [22], anti-inflammatory and analgesic activities [23].

The importance of bisindoles as bioactive intermediates in pharmaceutical industry cannot be underestimated. Bisindoles display a wide range of biological activities such as antimicrobial and antifungal [24], antibacterial [25], analgesic and anti-inflammatory [26] activities. Naturally occurring bisindoles such as hamacanthin A isolated from the sponge, *Hamacantha* sp [27] and *Spongosorites* sp [28] have shown potent antibacterial activity against *Staphylococcus aureus* and *Bacillus subtilis* [21]. Besides antitumor [29] and anticancer [30] activities, bisindoles have also been reported to be able to promote beneficial estrogen metabolism, promote useful estrogen, and induce apoptosis in human cancer cells [31]. Naturally occurring bisindoles such as bisindolylmethane or diindolylmethane are found mostly in cruciferous plants like broccoli, brussels sprouts, cauliflower, kale, and cabbage [31]. Medical epidemiologists believe that bisindolylmethane plays an important role in lowering the risk of cancer for people consuming plenty of cruciferous vegetables. Bisindolylmethane is also able to normalize abnormal cell growth associated with cervical dysplasia. Hong [32] and Kedmi [33] reported on the potential of bisindolylmethanes to proliferate and induce apoptosis in human prostate and breast cancer cells. Bhowmik [34] claimed that bisindolylmethane induces apoptosis in breast cancer cells by inhibiting epidermal growth factor receptor pathway. It has been reported that bisindolylmethane and its derivatives are potent inhibitors for *Leishmanial donovani* [35].

Schiff bases and bisindolylmethane had been reported to show potent antibacterial activity against various pathogenic bacteria [13,25]. Therefore, in this study, novel derivatives of bisindolylmethane Schiff bases have been synthesized and tested against pathogenic bacteria such as *Staphylococcus aureus,* *Salmonella typhi*, *S. paratyphi* A, *S. paratyphi* B and *Escherichia coli* to find out the effect of combining a Schiff base and bisindolylmethane.

## 2. Results and Discussion

### 2.1. Chemistry

**Scheme 1:**
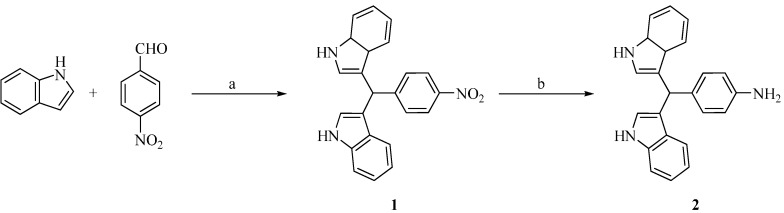
Reaction scheme for synthesis of compounds **1** and **2**.

**Scheme 2:**
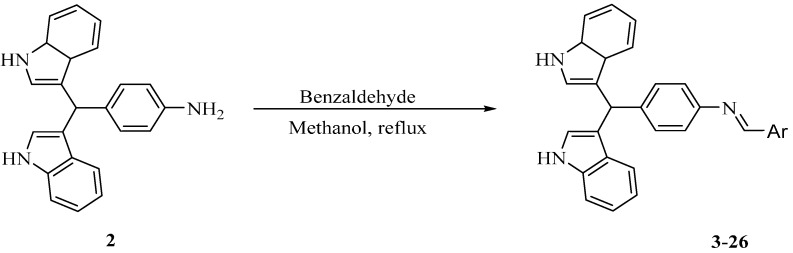
Reaction scheme for synthesis of bisindolylmethane Schiff bases (**3**–**26**).

All compounds in this study were synthesized by the method outlined in Scheme 1 and Scheme 2. Compound **1** was synthesized through acid catalyzed electrophilic substitution reaction of indole and aldehyde. Compound **1** was reduced to produce 4-(di(1*H*-indol-3-yl)methyl)aniline (**2**, Scheme 1). Acetic acid acts as the activating agent which increases electropositivity of electrophilic center on *p*-nitrobenzaldehyde and enables the addition of indole which act as a nucleophile to produce compound **1** which had been reported previously [36].

*Reagents and conditions*: (**a**) Glacial acetic acid, reflux; (**b**) NaBH_4_, Ni(OAc)_2_.4H_2_O, room temperature.

Reduction of the nitro substituent of compound **1** to an amino moiety was carried out using various reducing agents, as mentioned in Table 1. Since, there is a tendency for indole to be reduced to indoline, the reduction of compound **1** was studied using different catalysts to select the best one that provides the best yield for compound **2** in a reasonably short time. Besides the formation of indoline, uncontrolled reduction reactions could also result in production of azo compounds [37]. Other parameters such as the amount of reducing agent used and temperature were kept constant to observe the effect of different reducing agents on compound **1**. It was observed that *in situ* generation of nickel boride plays an important role in the reduction process.

**Table 1:** Synthesis of compound **2** using different reducing agents.

Reducing Agents	Nickel Acetate	Reaction Time	Yield (%)
Lithium aluminum hydride	+	2 h	54
Sodium borohydride	+	3 h	95
Lithium aluminum hydride	−	6 h	51
Sodium borohydride	−	4 h	78
Raney nickel	−	4 h	70
Hydrazine hydrate	−	15 h	76

+ Present; − Not present.

Observation shows that a mixture of sodium borohydride and nickel acetate provides the best yield in a fairly short reaction time (Table 1). The high yield obtained with sodium borohydride and nickel acetate evidences the *in situ* generation of nickel boride by the reaction of these reagents. Longer reaction times and lower yields were observed for the control experiment which was conducted without the presence of nickel acetate. This newly formed nickel boride resulting from the combination of sodium borohydride and nickel acetate is a milder reducing agent and able to selectively reduce the nitro group to an amino group without affecting the main indole skeleton. It was observed that there is not much difference in product yield when lithium aluminium hydride was used in the absence of nickel acetate, which suggest that of lithium aluminium hydride could reduce nitro group independently without the presence of nickel boride. The ^1^H-NMR spectrum of compound **2** shows the appearance of a singlet for NH_2_ at 4.81 ppm which was not observed in ^1^H-NMR spectra of compound **1**. Lithium aluminum hydride gives a shorter reaction time, but at the same time produces a low product yield compared to other catalysts. The presence of double bonds of bisindolylmethane would be the main reason for the reduced yield recorded by lithium aluminum hydride. The nonspecific reduction effect of other catalysts, especially lithium aluminum hydride, on both nitro and double bond of indole might have led to the formation of bisindoline as the minor product.

Compound **2** was converted to bisindolylmethane Schiff bases **3**–**26** with various substitution patterns (Table 2). Bisindolylmethane Schiff bases having imine functionalities were formed through a dehydration reaction between the aniline of compound **2** with various substituted benzaldehydes (Scheme 2).

The first part of the mechanism for Schiff base formation involves reaction of compound **2** with benzaldehyde which produces an unstable intermediate known as a carbinolamine which then undergoes dehydration to form an imine or Schiff base. A non-catalyzed reaction was employed in this study to prevent protonation of the amine. Protonation of the amine will shift the reaction equilibrium to the left, decreasing the formation of carbinolamine. Thus, most of the synthetic methods used for Schiff base synthesis employ either neutral or mildly acidic media.

**Table 2:** Structures of bisindolylmethane Schiff base derivatives.

Compound	Ar	Compound	Ar
**3**	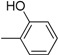	**15**	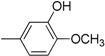
**4**	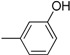	**16**	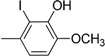
**5**	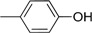	**17**	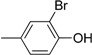
**6**	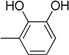	**18**	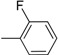
**7**	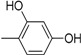	**19**	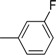
**8**	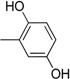	**20**	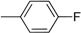
**9**	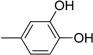	**21**	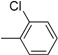
**10**	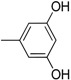	**22**	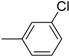
**11**	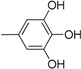	**23**	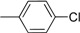
**12**	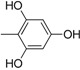	**24**	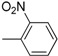
**13**	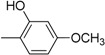	**25**	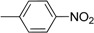
**14**	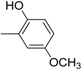	**26**	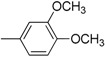

All compounds synthesized gave satisfactory spectroscopic information. The results obtained through ^1^H-NMR and mass spectrometry showed consistency with the assigned structures and molecular weight of the synthetic compounds. Functional group transformation was confirmed using FTIR. Compound **1** showed the presence of two important peaks at 1340 and 1543 cm^−1^ which indicates the presence of a NO_2_ group. Disappearance of these bands followed by the appearance of a NH_2_ absorption band at 3425 cm^−1^ suggests the successful conversion of NO_2_ to NH_2_.

^1^H-NMR was used to confirm the formation of the final Schiff base derivatives **3**–**26** by observing the disappearance of the NH_2_ peak at 4.81 ppm followed by the appearance of a singlet due to the C-H of the imine between 8.28–8.93 ppm. Other characteristic peaks include a singlet of one proton observed between 5.85–5.89 ppm, which represents the C-H of the bisindolylmethane methine linkage, and a singlet of two N-H protons on both indole rings, observed between 10.81–10.84 ppm.

### 2.2. Antibacterial Activity

As a continuation of our work on bioactive compounds [38,39,40,41,42,43]. Bisindolylmethane Schiff bases **3**–**26** were evaluated for their ability to inhibit both Gram-positive and Gram-negative bacterial strains using the disc diffusion method [44]. Gram-negative bacterial strains used in this study include *Escherichia coli*, *Klebsiella pneumoniae*, *Salmonella typhi*, *S. parathypi A*, *S. parathypi B*, *Shigella dysenteriae*, *S. flexneri*, *Acinobacter baumannii*, *Vibrio cholerae*. Results listed in Table 3 show that these derivatives display weak to moderate inhibitory activity against Gram-negative bacteria. Decreased or no activity against both Gram-negative and Gram-positive bacterial strains is generally observed for most compounds having hydroxyl groups substituted in any position, perhaps due to decrease in lipophilicity [45]. In accordance with Overtone’s concept of cell permeability, liposolubility plays an important role in manipulating antibacterial activity based on the ability of cell membranes to allow passage of only lipid-soluble materials [46]. Therefore, compounds having higher lipophilicity such as compounds with halogen groups have a greater capability of diffusing into bacteria and result in an increased antibacterial activity of those compounds. Further observation (Table 3) shows that derivatives having chloro and fluoro residues possessed the capability of inhibiting *S. typhi*, *S. parathypi A* and *S. parathypi B*. Compounds having halo, nitro and dimethoxy substituents displayed significant inhibitory activity. Compound **21** having a chloro group at the *ortho* position recorded the highest inhibition for *S. typhi* and *S. para typhi A* with inhibition zones of 14 and 12 mm, respectively. *S. para typhi B* inhibition was demonstrated by compound **20** bearing a fluoro substitution at the *para* position. Other compounds having chloro, fluoro and nitro substituents (**19**, **22** and **23**) showed comparably moderate activity against *S. paratyphi B*.

Compound **5**, having a hydroxy at the *para* position inhibited *S. dysenteriae* and *A. baumannii*. Compound **7**, **9**, and **10** which are dihydroxy-substituted compounds inhibited *K. pneumoniae*. Compound **8** which is also a dihydroxy-substituted compound showed inhibitory activity against *S. dysenteriae*. Compounds **11** and **12** which are trihydroxy-substituted compounds did not inhibit any of the tested Gram-negative bacterial strains. Compound **13**, **14**, **15**, and **16** having both hydroxy and methoxy substituents did not inhibit any Gram-negative bacterial strains. Compound **17** inhibited *S. flexneri*, while compound **18** inhibited *S. dysenteriae*. Compounds **19** and **20** having fluoro at the *meta* and *para* positions were capable of inhibiting three different strains of *Salmonella*. Compounds **21** and **22** having chloro at the *ortho* and *meta* position successfully inhibited the three different strains of *Salmonella*. Compound **26** which is a dimethoxy-substituted compound inhibited all three different strains of *Salmonella*. Compound **23** having a chloro at the *para* position showed activity against *S. typhi* and *S. paratyphi B*. Compounds **24** and **25** having nitro at *ortho* and *para* position displayed inhibition against *S. typhi* and *S. paratyphi B*. Compound **20** inhibited growth of *A. baumannii* and *V. cholerae*.

**Table 3:** Antibacterial activity of compounds **3**–**26** against Gram-negative bacteria (inhibition zones in mm using the disc diffusion method).

Compound	Inhibition Zone (mm)								
	A	B	C	D	E	F	G	H	I
**3**	-	-	-	-	-	-	-	-	-
**4**	-	-	-	-	-	-	-	-	-
**5**	-	-	-	-	-	11	-	09	-
**6**	-	-	-	-	-	-	-	-	-
**7**	-	07	-	-	-	-	-	-	-
**8**	-	-	-	-	-	10	-	-	-
**9**	-	09	-	-	-	-	-	-	-
**10**	-	06	-	-	-	-	-	-	-
**11**	-	-	-	-	-	-	-	-	-
**12**	-	-	-	-	-	-	-	-	-
**13**	-	-	-	-	-	-	-	-	-
**14**	-	-	-	-	-	-	-	-	-
**15**	-	-	-	-	-	-	-	-	-
**16**	-	-	-	-	-	-	-	-	-
**17**	-	-	-	-	-	-	07	-	-
**18**	-	-	-	-	-	10	-	-	-
**19**	-	-	08	10	15	-	-	-	-
**20**	10	-	12	09	17	-	-	12	13
**21**	-	-	14	12	16	-	-	-	12
**22**	-	-	11	10	15	-	-	-	-
**23**	-	-	10	-	16	-	-	-	10
**24**	09	-	09	-	11	-	-	-	-
**25**	-	-	10	-	09	-	-	-	-
**26**	-	-	08	08	10	-	-	-	-
**Gentamicin**	29	23	25	25	25	23	28	14	25

A = *Escherichia coli*, B = *Klebsiella pneumoniae*, C = *Salmonella typhi*, D = *Salmonella paratyphi A,* E = *Salmonella paratyphi B,* F = *Shigella dysenteriae,* G = *Shigella flexneri*, H = *Acinobacter baumannii*, I = *Vibrio cholera; -* No activity

Result for the inhibition activity of compounds **3**–**26** against Gram-positive bacterial strains are presented in Table 4, which shows that most of the derivatives having hydroxy residues were found to be inactive. Some derivatives having hydroxy substituents such as compounds **5**, **6** and **7** demonstrated weak activity against *Staphylococcus aureus.* Compound **8**, a derivative having a dihydroxy substituent showed weak inhibition against *S. aureus, S. epidermidis,* and MRSA. Comparison between hydroxyl- and non-hydroxy-substituted compounds reveals that compounds with chloro, fluoro, and nitro substituents exhibited better antibacterial activity in terms of number of bacterial strains inhibited. Compounds with halogens were found to be active against *Bacillus subtilis*, *Corynebacterium diphtheriae* and *S. aureus*, while compounds having nitro and dimethoxy substituents showed potential against *S. epidermidis* and *S. faecalis*. Compound **20** with a fluoro substituted at the *para* position inhibited all tested Gram-positive bacterial strains. However, compounds having a chloro substituent, such as **21** and **23** demonstrated inhibitory ability against most of the tested Gram-positive bacterial strains. Compound **21** with a chloro at the *ortho* position was found to be inactive against MRSA, while compound **23** with chloro substitution at the *para* position was observed to inhibit all the tested Gram-positive bacterial strains, except *B. subtilis* and MRSA.

**Table 4:** Antibacterial activity of compounds **3**–**26** against Gram-positive bacteria (inhibition zones in mm using the disk diffusion method).

Compound	Inhibition Zone (mm)					
	A	B	C	D	E	F
**3**	-	-	-	-	-	-
**4**	-	-	-	-	-	-
**5**	-	-	10	-	-	-
**6**	-	-	11	-	-	-
**7**	-	-	08	-	-	-
**8**	-	-	09	11	-	08
**9**	-	-	-	-	-	-
**10**	-	-	-	-	-	-
**11**	-	-	-	07	-	-
**12**	-	-	-	-	-	-
**13**	-	-	-	-	-	-
**14**	-	-	-	-	-	-
**15**	-	08	08	-	-	-
**16**	07	07	NA	09	08	-
**17**	-	-	-	-	10	-
**18**	12	08	09	10	-	-
**19**	-	09	-	09	12	09
**20**	11	10	09	11	11	-
**21**	09	13	08	12	10	-
**22**	09	-	07	-	09	-
**23**	-	07	08	08	12	-
**24**	-	-	-	07	09	-
**25**	-	-	-	09	-	-
**26**	-	-	-	08	08	-
**Gentamicin**	24	25	25	28	25	20

A = *Bacillus subtilis*, B = *Corynebacterium diphtheriae*, C = *Staphylococcus aureus*, D = *Staphylococcus epidermidis*, E = *Staphylococcus faecalis*, F = *MRSA*; - No activity.

## 3. Experimental Section

### 3.1. General Information

Melting points were determined on a Stuart Scientific SMP11 Analog melting point apparatus (Staffordshire, UK). IR spectra was obtained with a Perkin–Elmer system 2000 FTIR spectrophotometer (Norwalk, CT, USA) using KBr disks. NMR spectroscopy was performed on a Bruker Ultra Shield FT NMR 500 MHz spectrometer (Wissembourg, Switzerland). EI-MS spectroscopic analysis was carried out using Finnigan-MAT-311-A instrument (Bremen, Germany). Thin layer chromatography (TLC) was performed using precoated silica gel on aluminum sheets (Kieselgel 60 F-254, 0.20 mm, Merck, Darmstadt, Germany). Chromatograms were visualized using a handheld UV lamp at 254 nm (UVGL-58; UVP, Upland, CA, USA).

### 3.2. Synthesis of 3,3'-((4-Nitrophenyl)methylene)bis(1H-indole) (**1**)

Acetic acid (40 mL) was added to a mixture of indole (36 mmol) and *p*-nitrobenzaldehyde (18 mmol) in a round-bottomed flask and then refluxed. The progress of the reaction was monitored by TLC and upon completion; water was added to allow precipitate formation. The precipitate was filtered and dried at room temperature. The precipitate was finally rinsed with diethyl ether to afford pure product. Light yellow solid; Yield 92%; m.p. 219–221 °C; IR cm^−1^: 3420, 1543, 1457, 1342, 1226; ^1^H-NMR (DMSO-*d*_6_): *δ* 6.03 (s, 1H), 6.87–6.90 (m, 4H), 7.05 (t, *J =* 7.0 Hz, 2H), 7.29 (d, *J =* 6.5 Hz, 2H), 7.37 (d, *J =* 6.5 Hz, 2H), 7.62 (d, *J =* 6.5 Hz, 2H), 8.16 (d, *J =* 7.5 Hz, 2H), 11.05 (s, 2H); ^13^C-NMR (DMSO-*d*_6_): *δ* 40.35, 112.46, 112.46, 117.55, 117.55, 119.29, 119.29, 119.78, 119.78, 121.97, 121.97, 124.28, 124.28, 124.73, 124.73, 127.24, 130.32, 130.32, 137.47, 137.47, 146.63, 146.63, 154.00. EI MS *m/z* (% rel. abund.): 367.13 (100.0, M), 368.14 (25.1), 369.14 (3.4), 368.13 (1.1). Anal. Calcd for C_23_H_17_N_3_O_2_: C, 75.19; H, 4.66; N, 11.44; O, 8.71. Found: C, 75.18; H, 4.69; N, 11.43; O, 8.74.

### 3.3. Synthesis of 4-(di(1H-Indol-3-yl)methyl)aniline (**2**)

Acetonitrile (50 mL) was added to mixture of compound **1** (16.3 mmol) and nickel acetate tetrahydrate (3.3 mmol) in a round-bottomed flask and the mixture stirred in an ice bath. While stirring, sodium borohydride (66 mmol) was added in portions. Progress of the reaction was monitored using TLC. Upon completion water (90 mL) was added and stirred for another 3 min. The product was extracted using dichloromethane (100 mL × 3). The dichloromethane layer was collected and washed using a small quantity of water, dried over anhydrous Na_2_SO_4_, and evaporated under vacuum to give the crude product, which was rinsed with diethyl ether to afford pure product. Dark orange solid; Yield 95%; m.p. 230–232 °C; IR cm^−1^: 3425, 3387, 1629, 1276; ^1^H-NMR (DMSO-*d*_6_): *δ* 4.81 (s, 2H), 5.62 (s, 1H), 6.46 (d, *J =* 8.5 Hz, 2H), 6.76 (d, *J =* 2.0 Hz, 2H), 6.84 (t, *J =* 7.5 Hz, 2H), 6.98–7.03 (m, 4H), 7.26 (d, *J =* 8.0 Hz, 2H), 7.32 (d, *J =* 8.0 Hz, 2H), 10.72 (s, 2H); ^13^C-NMR (MeOD-*d*_4_): *δ* 39.88, 110.72, 110.72, 115.51, 115.51, 118.08, 118.08, 118.48, 118.48, 119.75, 119.75, 120.86, 120.86, 123.40, 123.40, 126.97, 126.97, 129.59, 129.59, 137.10, 137.01, 144.01. EI MS *m/z* (% rel. abund.): 337.16 (100.0, M), 338.16 (25.1), 339.16 (3.2), 338.15 (1.1). Anal. Calcd for C_23_H_19_N_3_: C, 81.87; H, 5.68; N, 12.45. Found: C, 81.89; H, 5.70; N, 12.44.

### General Procedure for Synthesis of Bisindolylmethane Schiff bases **3**–**26**

Methanol (20 mL) was added to a mixture of compound **2** (0.5 mmol) and a substituted benzaldehyde (0.5 mmol) in round-bottomed flask and the resulting mixture was refluxed until TLC monitoring of the progress of the reaction indicated its completion. The solvent was then removed using a rotary evaporator and the crude product was collected and rinsed using hot hexane to afford pure product.

*(E)-2-(((4-(di(1H-Indol-3-yl)methyl)phenyl)imino)methyl)phenol* (**3**). Dark orange solid; Yield 74%; m.p. 209–211 °C; IR cm^−1^: 3572, 3389, 1670, 1590, 1361, 1222; ^1^H-NMR (DMSO-*d*_6_): *δ* 5.89 (s, 1H), 6.88 (dd, *J* = 12.5 Hz, 5.1 Hz, 4H), 6.97 (dd, *J* = 13.7 Hz, 7.3 Hz, 2H), 7.05 (dd, *J* = 11.6 Hz, 4.4 Hz, 2H), 7.31 (d, *J* = 8.0 Hz, 2H), 7.34 (d, *J* = 8.4 Hz, 2H), 7.37 (d, *J* = 8.1 Hz, 2H), 7.39–7.42 (m, 1H), 7.44 (d, *J* = 8.4 Hz, 2H), 7.62 (dd, *J* = 7.7 Hz, 1.6 Hz, 1H), 8.96 (s, 1H), 10.84 (s, 2H). ^13^C-NMR (DMSO-*d*_6_): *δ* 39.84, 111.96, 111.96, 117.02, 118.34, 118.34, 118.71, 118.71 119.57, 119.57, 121.41, 121.41, 121.55, 124.07, 124.07, 127.06, 127.06, 129.77, 132.95, 132.95, 133.54, 136.89, 137.10, 137.10, 144.53, 146.17, 149.48, 160.77, 160.77, 163.22. EI MS *m/z* (% rel. abund.): 441.05 (100.0, M), 325.03 (5.0), 321.01 (4.9), 245.03 (29.0), 196.00 (0.7), 204.01 (6.9). Anal. Calcd for C_30_H_23_N_3_O: C, 81.61; H, 5.25; N, 9.52; O, 3.62. Found: C, 81.63; H, 5.27; N, 9.54; O, 3.59.

*(E)-3-(((4-(di(1H-Indol-3-yl)methyl)phenyl)imino)methyl)phenol* (**4**). Light orange solid; Yield 68%; m.p. 138–140 °C; IR cm^−1^: 3565, 3383, 1652, 1602, 1335, 1220; ^1^H-NMR (DMSO-*d*_6_): *δ* 5.86 (s, 1H), 6.85–6.90 (m, 4H), 7.05 (t, *J =* 8.0 Hz, 2H), 7.18 (d, *J =* 8.5 Hz, 2H), 7.30–7.31 (m, 4H), 7.35–7.40 (m, 5H), 8.52 (s, 1H), 10.83 (s, 2H); ^13^C-NMR (DMSO-*d*_6_): *δ* 39.79, 103.04, 111.92, 111.92, 114.61, 117.61, 118.51, 118.51, 118.65, 118.65, 119.59, 119.59, 120.63, 121.21, 121.21, 121.34, 124.01, 124.01, 127.09, 127.09, 129.50, 130.26, 130.26, 137.09, 137.09, 158.14, 160.35, 160.35, 162.77, 170.85. EI MS *m/z* (% rel. abund.): 440.99 (100.0, M), 335.99 (33.8), 323.99 (11.3), 244.99 (45.5), 116.99 (27.8). Anal. Calcd for C_30_H_23_N_3_O: C, 81.61; H, 5.25; N, 9.52; O, 3.62. Found: C, 81.62; H, 5.24; N, 9.54; O, 3.61.

*(E)-3-(((4-(di(1H-Indol-3-yl)methyl)phenyl)imino)methyl)phenol* (**5**). Brownish red solid; Yield 86%; m.p. 118–120 °C; IR cm^−1^: 3551, 3386, 1664, 1578, 1365, 1210; ^1^H-NMR (DMSO-*d*_6_): *δ* 5.84 (s, 1H), 6.85–6.89 (m, 7H), 7.04 (t, *J =* 8.0 Hz, 2H), 7.12 (d, *J =* 8.5 Hz, 2H), 7.30 (d, *J =* 8.0 Hz, 2H), 7.35 (dd, *J =* 2.0 Hz, 8 Hz, 4H), 7.73 (d, *J =* 8.5 Hz, 2H), 8.45 (s, 1H), 10.82 (s, 2H); ^13^C-NMR (MeOD-*d*_4_): *δ* 39.84, 110.82, 110.82, 115.37, 118.12, 118.12, 118.67, 118.67, 119.16, 119.16, 120.30, 120.30, 120.90, 120.90, 123.42, 123.42, 127.00, 127.00, 129.31, 130.61, 130.61, 137.09, 137.09 142.82, 149.48, 158.17, 158.17 160.26, 160.26, 162.53. EI MS *m/z* (% rel. abund.): 441.00 (100.0, M), 337.00 (59.7), 244.99 (39.9), 116.99 (9.7). Anal. Calcd for C_30_H_23_N_3_O: C, 81.61; H, 5.25; N, 9.52; O, 3.62. Found: C, 81.64; H, 5.23; N, 9.53; O, 3.60.

*(E)-3-(((4-(di(1H-Indol-3-yl)methyl)phenyl)imino)methyl)benzene-1,2-diol* (**6**). Light orange solid; Yield 71%; m.p. 157–159 °C; IR cm^−1^: 3549, 3378, 1673, 1585, 1359, 1223; ^1^H-NMR (DMSO-*d*_6_): *δ* 5.89 (s, 1H), 6.75 (t, *J =* 8.0 Hz, 1H), 6.87–6.92 (m, 4H), 6.93 (d, *J =* 1.0 Hz, 1H), 7.03–7.07 (m, 3H), 7.31 (d, *J =* 8.0 Hz, 2H), 7.34 (d, *J =* 8.5 Hz, 2H), 7.36 (d, *J =* 8.0 Hz, 2H), 7.44 (d, *J =* 8.0 Hz, 2H), 8.90 (s, 1H), 10.84 (s, 2H); ^13^C-NMR (MeOD-*d*_4_): *δ* 39.89, 110.82, 110.82, 118.02, 118.12, 118.12, 118.43, 118.43, 119.07, 119.28 119.28, 120.39, 120.39, 120.91 120.91, 122.86, 123.42, 123.42, 126.95, 126.95, 129.52, 129.52, 137.11, 137.11, 144.16, 145.37, 145.68, 149.75, 162.13, 171.68. EI MS *m/z* (% rel. abund.): 456.98 (100.0, M), 337.00 (49.2), 321.97 (6.7), 244.99 (39.7), 116.97 (2.9). Anal. Calcd for C_30_H_23_N_3_O_2_: C, 78.75; H, 5.07; N, 9.18; O, 6.99. Found: C, 78.76; H, 5.08; N, 9.16; O, 6.96.

*(E)-3-(((4-(di(1H-Indol-3-yl)methyl)phenyl)imino)methyl)benzene-2,4-diol* (**7**). Dark red solid; Yield 88%; m.p. 153–155 °C; IR cm^−1^: 3550, 3395, 1667, 1587, 1361, 1220; ^1^H-NMR (DMSO-*d*_6_): *δ* 5.86 (s, 1H), 6.27 (d, *J =* 2.5 Hz, 1H), 6.38 (dd, *J =* 2.5 Hz, 8.5 Hz, 1H), 6.85–6.89 (m, 4H), 7.04 (t, *J =* 7.0 Hz, 2H), 7.26 (d, *J =* 8.5 Hz, 2H), 7.30 (d, *J =* 8.0 Hz, 2H), 7.35 (d, *J =* 8.5 Hz, 2H), 7.38-7.40 (m, 3H), 8.77 (s, 1H),10.83 (s, 2H); ^13^C-NMR (MeOD-*d*_4_): *δ* 39.88, 60.35, 94.16, 107.60, 108.55, 110.79, 110.79, 112.21, 118.08, 118.08, 118.46, 118.46, 119.04, 119.04, 119.70, 119.70, 120.88, 120.88, 123.39, 123.39, 126.95, 126.95, 129.55, 129.55, 134.29, 137.11, 137.11, 143.51, 144.31, 159.98. EI MS *m/z* (% rel. abund.): 456.98 (28.9), 337.00 (100.0, M), 244.99 (26.0), 116.99 (18.9). Anal. Calcd for C_30_H_23_N_3_O_2_: C, 78.75; H, 5.07; N, 9.18; O, 6.99. Found: C, 78.73; H, 5.10; N, 9.19; O, 7.01.

*(E)-3-(((4-(di(1H-Indol-3-yl)methyl)phenyl)imino)methyl)benzene-2,5-diol* (**8**). Dark red solid; Yield 76%; m.p. 103–105 °C; IR cm^−1^: 3561, 3383, 1664, 1584, 1363, 1223; ^1^H-NMR (DMSO-*d*_6_): *δ* 5.88 (s, 1H), 6.78 (d, *J =* 9.0 Hz, 1H), 6.84–6.90 (m, 5H), 7.00 (d, *J =* 3 Hz, 1H), 7.04 (t, *J =* 7 Hz, 2H), 7.31 (d, *J =* 8.5 Hz, 4H), 7.36 (d, *J =* 8.5 Hz, 2H), 7.42 (d, *J =* 8.5 Hz, 2H), 8.82 (s, 1H), 9.16 (s, 1H), 10.84 (s, 2H), 12.36 (s, 1H); ^13^C-NMR (MeOD-*d*_4_): *δ* 39.79, 110.80, 110.80, 116.87, 116.96, 117.60, 118.08, 118.08, 118.46, 118.46, 119.04, 119.31, 119.31, 120.41, 120.41 120.47, 120.88, 123.40, 123.40, 126.95, 126.95, 129.48, 129.48, 137.11, 137.11, 144.09, 144.09, 146.37, 149.44, 161.91.EI MS *m/z* (% rel. abund.): 456.98 (100.0, M),337.00(92.3), 245.03 (53.0), 116.99 (15.8). Anal. Calcd for C_30_H_23_N_3_O_2_: C, 78.75; H, 5.07; N, 9.18; O, 6.99. Found: C, 78.76; H, 5.06; N, 9.16; O, 6.96.

*(E)-3-(((4-(di(1H-Indol-3-yl)methyl)phenyl)imino)methyl)benzene-3,4-diol* (**9**). Dark red solid; Yield 88%; m.p. 144–145 °C; IR cm^−1^: 3554, 3390, 1670, 1585, 1365, 1225; ^1^H-NMR (DMSO-*d*_6_): *δ* 5.84 (s, 1H), 6.81 (d, *J =* 8.0 Hz, 1H), 6.85 (s, 2H), 6.87 (t, *J =* 8.0 Hz, 2H), 7.04 (t, *J =* 7.5 Hz, 2H), 7.11 (d, *J =* 8.5 Hz, 2H), 7.15 (dd, *J =* 2.0 Hz, 8.0 Hz, 1H), 7.30 (d, *J =* 7.5 Hz, 2H), 7.34–7.36 (m, 5H), 8.36 (s, 1H), 10.81 (s, 2H), 12.01 (s, 1H); ^13^C-NMR (MeOD-*d*_4_): *δ* 39.89, 56.08, 110.72, 110.81, 110.81, 113.52, 113.62, 114.12, 118.09, 118.09, 118.64, 118.64, 119.11, 120.30, 120.30, 120.88, 120.88, 122.57, 122.57, 122.92, 123.41, 124.71, 126.99, 126.99, 129.28, 129.28, 137.09, 137.09, 153.59, 160.75. EI MS *m/z* (% rel. abund.): 456.98 (10.2), 336.95 (100.0, M), 244.96 (49.3), 116.96 (94.5). Anal. Calcd for C_30_H_23_N_3_O_2_: C, 78.75; H, 5.07; N, 9.18; O, 6.99. Found: C, 78.73; H, 5.09; N, 9.21; O, 6.98.

*(E)-3-(((4-(di(1H-Indol-3-yl)methyl)phenyl)imino)methyl)benzene-3,5-diol* (**10**). Brown solid; Yield 70%; m.p. 222–224 °C; IR cm^−1^: 3541, 3376, 1665, 1589, 1357, 1224; ^1^H-NMR (DMSO-*d*_6_): *δ* 5.85 (s, 1H), 6.34 (t, *J =* 2.0 Hz, 1H), 6.77 (d, *J =* 2.0 Hz, 2H), 6.85–6.89 (m, 4H), 7.04 (t, *J =* 7.0 Hz, 2H), 7.15 (d, *J =* 8.5 Hz, 2H), 7.30 (d, *J =* 8.0 Hz, 2H), 7.30–7.38 (m, 4H), 8.39 (s, 1H), 10.83 (s, 2H), 12.15 (s, 2H); ^13^C-NMR (DMSO-*d*_6_): *δ* 39.96, 107.10, 107.73, 111.80, 111.80, 111.93, 118.44, 118.44, 118.52, 118.52, 118.66, 118.66, 119.47, 119.47, 119.59, 119.59, 119.70, 119.70, 121.20, 121.36, 121.36, 123.76, 124.01, 127.09, 127.09, 129.48, 129.48, 137.08, 137.08, 159.12. EI MS *m/z* (% rel. abund.): 456.98 (1.1), 336.95 (100.0, M), 244.96 (49.3), 116.96 (94.5). Anal. Calcd for C_30_H_23_N_3_O_2_: C, 78.75; H, 5.07; N, 9.18; O, 6.99. Found: C, 78.76; H, 5.05; N, 9.20; O, 7.01.

*(E)-5-(((4-(di(1H-Indol-3-yl)methyl)phenyl)imino)methyl)benzene-1,2,3-triol* (**11**). Dark yellow solid; Yield 75%; m.p. 201–202 °C; IR cm^−1^: 3564, 3362, 1662, 1603, 1357, 1220; ^1^H-NMR (DMSO-*d*_6_): *δ* 5.88 (s, 1H), 6.85–6.89 (m, 6H), 7.05 (t, *J =* 7.0 Hz, 2H), 7.11 (d, *J =* 8.0 Hz, 2H), 7.31 (d, *J =* 7.5 Hz, 2H), 7.35-7.37 (m, 4H), 8.28 (s, 1H), 9.04(s, 3H), 10.82 (s, 2H); ^13^C-NMR (MeOD-*d*_4_): *δ* 39.84, 108.15, 110.78, 110.78, 115.44, 118.05, 118.05, 118.64, 118.64, 119.08, 119.08, 120.11, 120.11, 120.85, 120.85, 123.39, 123.39, 126.99, 126.99, 129.31, 129.31, 137.11, 137.11, 142.79, 145.83, 160.88, 160.88, 171.69, 171.69, 191.91. EI MS *m/z* (% rel. abund.): 472.99 (100.0, M), 336.95 (38.7), 244.99 (40.6), 116.99 (58.8). Anal. Calcd for C_30_H_23_N_3_O_3_: C, 76.09; H, 4.90; N, 8.87; O, 10.14. Found: C, 76.12; H, 4.92; N, 8.85; O, 10.16.

*(E)-5-(((4-(di(1H-Indol-3-yl)methyl)phenyl)imino)methyl)benzene-1,3,5-triol* (**12**). Dark orange solid; Yield 83%; m.p. 215–217 °C; IR cm^−1^: 3553, 3364, 1668, 1578, 1360, 1220; ^1^H-NMR (DMSO-*d*_6_): *δ* 5.80 (s, 2H), 5.85 (s, 1H), 6.85–6.89 (m, 4H), 7.04 (t, *J =* 7.0 Hz, 2H), 7.20 (d, *J =* 10.0 Hz, 2H), 7.30 (d, *J =* 8.0 Hz, 2H), 7.35 (d, *J =* 8.0 Hz, 2H), 7.38 (d, *J =* 8.0 Hz, 2H), 8.90 (s, 1H), 10.82 (s, 2H), 12.22 (s, 1H); ^13^C-NMR (MeOD-*d*_4_): *δ* 39.81, 60.14, 102.65, 110.78, 110.78, 118.09, 118.09, 118.39, 118.39, 118.61, 119.01, 119.01, 120.88, 120.88, 123.40, 123.40, 126.52, 126.93, 126.93, 129.76, 129.76, 137.11, 137.11, 143.37, 143.37, 152.85, 162.13, 162.13, 171.68, 171.68. EI MS *m/z* (% rel. abund.): 472.96 (100.0, M), 336.95 (32.8), 245.02 (5.8), 116.99 (16.1). Anal. Calcd for C_30_H_23_N_3_O_3_: C, 76.09; H, 4.90; N, 8.87; O, 10.14. Found: C, 76.06; H, 4.88; N, 8.89; O, 10.15.

*(E)-2-(((4-(di(1H-Indol-3-yl)methyl)phenyl)imino)methyl)-5-methoxyphenol* (**13**). Dark orange solid; Yield 74%; m.p. 121–123 °C; IR cm^-1^: 3552, 3383, 1665, 1587, 1361, 1225; ^1^H-NMR (DMSO-*d*_6_): *δ* 3.85 (s, 3H), 5.88 (s, 1H), 6.48 (d, *J =* 2.0 Hz, 1H), 6.54 (dd, *J =* 2.0 Hz, 8.5Hz, 1H), 6.87–6.90 (m, 4H), 7.05 (t, *J =* 7.0 Hz, 2H), 7.31 (t, *J =* 7.5 Hz, 4H), 7.37 (d, *J =* 8.0 Hz, 2H), 7.42 (d, *J =* 8.5 Hz, 2H), 7.50 (d, *J =* 8.5 Hz, 2H), 8.85 (s, 1H), 10.84 (s, 2H); ^13^C-NMR (MeOD-*d*_4_): *δ* 39.87, 54.50, 100.80, 106.66, 110.78, 110.78, 115.46, 115.46, 118.06, 118.06, 118.44, 118.44, 119.02, 119.02, 119.79, 119.79, 120.87, 120.87, 123.38, 123.38, 126.95, 126.95, 129.57, 129.57, 133.81, 137.12, 144.00, 160.06, 165.29, 167.29, 186.82. EI MS *m/z* (% rel. abund.): 471.00 (100.0, M), 354.05 (21.3), 337.07 (12.4), 245.12 (51.7), 117.08 (10.0). Anal. Calcd for C_31_H_25_N_3_O_2_: C, 78.96; H, 5.34; N, 8.91; O, 6.79. Found: C, 78.95; H, 5.32; N, 8.92; O, 6.76.

*(E)-2-(((4-(di(1H-Indol-3-yl)methyl)phenyl)imino)methyl)-4-methoxyphenol* (**14**). Light orange solid; Yield 76%; m.p. 207–209 °C; IR cm^−1^: 3548, 3375, 1661, 1571, 1353, 1220; ^1^H-NMR (DMSO-*d*_6_): *δ* 3.74 (s, 3H), 5.89 (s, 1H), 6.86–6.90 (m, 5H), 7.01–7.06 (m, 3H), 7.22 (d, *J =* 3.0 Hz, 1H), 7.31 (t, *J =* 8.0 Hz, 4H), 7.36 (d, *J =* 8.5 Hz, 2H), 7.43 (d, *J =* 8.0 Hz, 2H), 8.91 (s, 1H), 9.95 (s, 1H), 10.84 (s, 2H); ^13^C-NMR (DMSO-*d*_6_): *δ* 39.95, 56.03, 111.96, 111.96, 115.72, 115.72, 117.88, 117.88, 118.35, 118.35, 118.70, 119.58, 119.58, 119.68, 119.68, 120.70, 121.40, 121.52, 124.08, 124.08, 127.07, 127.07, 129.77, 129.77, 137.11, 137.11, 144.49, 146.43, 152.33, 154.82, 162.79. EI MS *m/z* (% rel. abund.): 471.00 (100.0, M), 354.07 (19.7), 337.13 (48.3), 321.06 (15.7), 117.09 (17.8). Anal. Calcd for C_31_H_25_N_3_O_2_: C, 78.96; H, 5.34; N, 8.91; O, 6.79. Found: C, 78.97; H, 5.36; N, 8.93; O, 6.76.

*(E)-5-(((4-(di(1H-Indol-3-yl)methyl)phenyl)imino)methyl)-2-methoxyphenol* (**15**). Brown solid; Yield 78%; m.p. 132–134 °C; IR cm^−1^: 3566, 3382, 1668, 1584, 1362, 1222, ^1^H-NMR (DMSO-*d*_6_): *δ* 3.83 (s, 3H), 5.86 (s,1H), 6.86–6.89 (m, 4H), 7.01–7.06 (m, 4H), 7.14 (d, *J =* 8.5 Hz, 2H), 7.31 (d, *J =* 8.0 Hz, 2H), 7.354–7.377 (m, 4H), 7.42 (s, 1H), 8.43 (s, 1H), 9.37 (s, 1H), 10.82 (s, 2H); ^13^C-NMR (MeOD-*d*_4_) *δ* 39.89, 56.08, 110.72, 110.72, 110.81, 113.52, 113.62, 113.62, 114.12, 118.09, 118.09, 118.64, 118.64, 119.11, 119.11, 120.30, 120.30, 120.88, 122.57, 122.92, 123.41, 123.41, 124.71, 126.99, 126.99, 129.28, 129.28, 137.09, 137.09, 153.59, 160.75. EI MS *m/z* (% rel. abund.): 471.00 (100.0, M), 53.00 (11.3), 337.05 (10.9), 245.09 (27.0), 117.09 (9.3). Anal. Calcd for C_31_H_25_N_3_O_2_: C, 78.96; H, 5.34; N, 8.91; O, 6.79. Found: C, 78.98; H, 5.32; N, 8.94; O, 6.76.

*(E)-3-(((4-(di(1H-Indol-3-yl)methyl)phenyl)imino)methyl)-2-iodo-6-methoxyphenol* (**16**). Dark red solid; Yield 86%; m.p. 135–137 °C; IR cm^−1^: 3556, 3387, 1673, 1582, 1357, 1222; ^1^H-NMR (DMSO-*d*_6_): *δ* 3.89 (s, 1H), 5.86 (s,1H), 6.86–6.89 (m, 4H), 7.05 (t, *J =* 7.0 Hz, 2H), 7.10 (d, *J =* 8.5 Hz, 1H), 7.14 (d, *J =* 8.5 Hz, 2H), 7.31 (d, *J =* 8.0 Hz, 2H), 7.36 (d, *J =* 8.0 Hz, 2H), 7.40 (d, *J =* 8.5 Hz, 2H), 7.59 (d, *J =* 8.5 Hz, 2H), 8.62 (d, 2H), 10.83 (s, 2H), 12.13 (s, 1H),; ^13^C-NMR (MeOD-*d*_4_): *δ* 38.68, 110.11, 110.11, 110.50, 110.81, 113.18, 113.18, 114.21, 118.11, 118.11, 118.58, 118.58, 119.10, 119.10, 120.11, 120.11, 120.47, 120.89, 122.69, 123.05, 123.05, 123.45, 126.98, 126.98, 128.69, 129.39, 129.39, 137.09, 137.09, 152.14, 164.28. EI MS *m/z* (% rel. abund.): 597.00 (2.7), 471.11 (80.0), 337.11 (78.4), 245.11 (53.5), 117.08 (100.0, M). Anal. Calcd for C_31_H_24_IN_3_O_2_: C, 62.32; H, 4.05; I, 21.24; N, 7.03; O, 5.36. Found: C, 62.35; H, 4.07; I, 21.25; N, 7.01; O, 5.34.

*(E)-2-Bromo-4-(((4-(di(1H-indol-3-yl)methyl)phenyl)imino)methyl)phenol* (**17**). Light red solid; Yield 78%; m.p. 139–141 °C; IR cm^−1^: 3552, 3384, 1667, 1587, 1350, 1215, 1060; ^1^H-NMR (DMSO-*d*_6_): *δ* 5.85 (s,1H), 6.85–6.89 (m, 4H), 7.03–7.06 (m, 3H), 7.15 (d, *J =* 7.5 Hz, 2H), 7.30 (d, *J =* 8.0 Hz, 2H), 7.35-7.38 (m, 4H), 7.74 (d, *J =* 8.5 Hz, 2H), 8.03 (s, 1H), 8.47 (s, 1H), 10.82 (s, 2H), 11.87 (s, 1H); ^13^C-NMR (MeOD-*d*_4_): *δ* 39.86, 110.43, 110.43, 110.83, 116.11, 118.13, 118.13, 118.60, 118.60, 119.15, 119.15, 120.29, 120.29, 120.91, 123.44, 123.44, 126.98, 126.98, 129.33, 129.33, 129.52, 129.52, 133.49, 137.08, 137.08, 143.16, 148.73, 157.94, 158.91, 190.12. EI MS *m/z* (% rel. abund.): 520.95 (100.0, M), 441.06 (14.8), 337.05 (81.5), 245.07 (67.3), 117.05 (25.3). Anal. Calcd for C_30_H_22_BrN_3_O: C, 69.24; H, 4.26; Br, 15.35; N, 8.07; O, 3.07. Found: C, 69.25; H, 4.25; Br, 15.37; N, 8.09; O, 3.09.

*(E)-4-(di(1H-Indol-3-yl)methyl)-N-(2-fluorobenzylidene)aniline* (**18**). Dark orange solid; Yield 64%; m.p. 120–122 °C; IR cm^−1^: 3396, 1661, 1591, 1357, 1235 (C–F); ^1^H-NMR (DMSO-*d*_6_): *δ* 5.88 (s, 1H), 6.86–6.89 (m, 4H), 7.03 (t, *J =* 8.0 Hz, 2H), 7.23–7.25 (m, 3H), 7.30–7.36 (m, 7H), 7.40 (d, *J =* 8.5 Hz, 2H), 8.78 (s, 1H), 10.83 (s, 2H); ^13^C-HMR (MeOD-*d*_4_): *δ* 39.87, 110.68, 110.68, 110.81, 115.52, 117.87, 118.08, 118.08, 118.51, 118.51, 119.05, 119.05, 120.40, 120.40, 120.70, 120.70, 120.88, 123.41, 123.41, 124.37, 126.96, 126.96, 128.29, 129.38, 129.38, 137.10, 137.10, 143.81, 153.30 (d, *J =* 206.25 Hz).EI MS *m/z* (% rel. abund.): 442.95 (100.0, M), 325.98 (34.5), 245.08 (72.5), 117.06 (34.5). Anal. Calcd for C_30_H_22_FN_3_: Elemental Analysis: C, 81.24; H, 5.00; F, 4.28; N, 9.47. Found: Elemental Analysis: C, 81.25; H, 4.98; F, 4.29; N, 9.49.

*(E)-4-(di(1H-Indol-3-yl)methyl)-N-(3-fluorobenzylidene)aniline* (**19**). Light orange solid; Yield 66%; m.p. 139–141 °C; IR cm^−1^: 3392, 1661, 1591, 1364, 1238; ^1^H-NMR (DMSO-*d*_6_): *δ* 5.87 (s, 1H), 6.86–6.89 (m, 4H), 7.00 (t, *J =* 9.0 Hz, 1H), 7.05 (t, *J =* 7.0 Hz, 2H), 7.22 (d, *J =* 8.0 Hz, 2H), 7.31 (d, *J =* 8.5 Hz, 2H), 7.36 (d, *J =* 8.5 Hz, 2H), 7.41 (d, *J =* 8.5 Hz, 2H), 7.56 (d, *J =* 5.5 Hz, 1H), 7.70 (d, *J =* 9.5 Hz, 2H), 7.75 (d, *J =* 7.5 Hz, 2H), 8.66 (s, 1H), 10.83 (s, 2H); ^13^C-NMR (MeOD-*d*_4_): *δ* 39.88, 110.83, 110.83,113.80, 113.98, 113.98, 118.12, 118.12, 118.54, 118.54, 119.11, 119.11, 120.43, 120.43, 120.91, 120.91, 123.43, 123.43, 124.85, 126.97, 126.97, 129.34, 129.34, 130.24, 130.31, 137.10, 137.10, 143.71, 148.81, 159.07 (d, *J =* 203.75 Hz), 174.86. EI MS *m/z* (% rel. abund.): 442.95 (100.0, M), 325.98 (22.9), 245.08 (64.4), 117.06 (5.4). Anal. Calcd for C_30_H_22_FN_3_: Elemental Analysis: C, 81.24; H, 5.00; F, 4.28; N, 9.47. Found: Elemental Analysis: C, 81.21; H, 5.01; F, 4.30; N, 9.50.

*(E)-4-(di(1H-Indol-3-yl)methyl)-N-(4-fluorobenzylidene)aniline* (**20**). Dark red solid; Yield 78%; m.p. 139–141 °C; IR cm^−1^: 3397, 1664, 1583, 1359, 1235; ^1^H-NMR (DMSO-*d*_6_): *δ* 5.87 (s, 1H), 6.76 (s, 1H), 6.83–6.89 (m, 4H), 7.00 (t, *J =* 8.5 Hz ,2H), 7.04 (d, *J =* 7.0 Hz, 2H), 7.19 (d, *J =* 8.0 Hz, 2H), 7.22 (d, *J =* 7.5 Hz, 1H), 7.30–7.36 (m, 5H), 7.39 (d, *J =* 8.0 Hz, 1H), 8.63 (s, 1H), 10.83 (s, 2H); ^13^C-NMR (MeOD-*d*_4_): *δ* 39.86, 110.85, 110.85, 115.30, 115.30, 115.47, 115.47, 118.14, 118.14, 118.58, 118.58, 119.13, 119.13, 120.37, 120.37, 120.92, 120.92, 123.44, 123.44, 126.97, 126.97, 130.60, 130.67, 130.67, 137.08, 137.08, 143.37, 143.37, 149.07, 159.21 (d, *J =* 228.75 Hz). EI MS *m/z* (% rel. abund.): 442.95 (100.0, M), 325.92 (14.4), 245.04 (53.4), 117.06 (3.9). Anal. Calcd for C_30_H_22_FN_3_: Elemental Analysis: C, 81.24; H, 5.00; F, 4.28; N, 9.47. Found: Elemental Analysis: C, 81.32; H, 5.02; F, 4.31; N, 9.45.

*(E)-4-(di(1H-Indol-3-yl)methyl)-N-(2-chlorobenzylidene)aniline* (**21**). Dark red solid; Yield 84%; m.p. 145–147 °C; IR cm^−1^: 3386, 1668, 1584, 1363, 1092; ^1^H-NMR (DMSO-*d*_6_): *δ* 5.88 (s, 1H), 6.87–6.90 (m, 4H), 7.05 (t, *J =* 8.0 Hz, 2H), 7.24 (d, *J =* 8.5 Hz, 2H), 7.31 (d, *J =* 8.0 Hz, 2H), 7.36 (d, *J =* 8.0 Hz, 2H), 7.42(d, *J =* 8.5 Hz, 2H), 7.48 (t, *J =* 7.5 Hz, 1H), 7.48 (t, *J =* 7.5 Hz, 1H), 7.55 (t, *J =* 7.5 Hz, 1H), 7.58 (dd, *J =* 1 Hz, 8.0 Hz, 1H), 8.15 (dd, *J =* 1.5 Hz, 8.0 Hz, 1H), 8.88 (d, 1H), 10.84 (s, 2H); ^13^C-NMR (DMSO-*d*_6_): *δ* 39.79, 103.04, 111.92, 111.92, 114.61, 117.61, 118.51, 118.51, 118.65, 118.65, 119.59, 119.59, 120.63, 120.63, 121.21, 121.34, 124.01, 124.01, 127.09, 127.09, 129.50, 129.50, 130.26, 130.26, 137.09, 137.09, 158.14, 160.35, 162.77, 170.85. EI MS *m/z* (% rel. abund.): 458.95 (100.0, M), 337.03 (60.8), 245.05 (85.1), 117.04 (4.9). Anal. Calcd for C_30_H_22_ClN_3_: C, 78.34; H, 4.82; Cl, 7.71; N, 9.14. Found: C, 78.36; H, 4.81; Cl, 7.74; N, 9.16.

*(E)-4-(di(1H-Indol-3-yl)methyl)-N-(3-chlorobenzylidene)aniline* (**22**). Light orange solid; Yield 75%; m.p. 148–150 °C; IR cm^−1^: 3390, 1674, 1586, 1365, 1090; ^1^H-NMR (DMSO-*d*_6_): *δ* 5.87 (s, 1H), 6.86–6.89 (m, 4H), 7.05 (t, *J =* 7 Hz, 2H), 7.23 (d, *J =* 8 Hz, 2H), 7.31 (d, *J =* 8.0 Hz, 2H), 7.36 (d, *J =* 7.5 Hz, 2H), 7.54 (m, 3H), 7.87 (d, *J =* 7.5 Hz, 2H), 8.65 (s, 1H), 10.83 (s, 2H); ^13^C-NMR (MeOD-*d*_4_): *δ* 39.89, 110.80, 110.80, 113.20, 118.07, 118.07, 118.52, 118.52, 119.07, 120.44, 120.44, 120.87, 120.87, 123.39, 123.39, 126.97, 126.97, 127.70, 129.34, 129.34, 129.98, 129.98, 130.76, 134.55, 137.11, 137.11, 138.16, 143.78, 149.01, 158.82. EI MS *m/z* (% rel. abund.): 458.95 (100.0, M), 337.10 (45.3), 245.10 (66.6), 117.06 (3.4). Anal. Calcd for C_30_H_22_ClN_3_: C, 78.34; H, 4.82; Cl, 7.71; N, 9.14. Found: C, 78.36; H, 4.85; Cl, 7.69; N, 9.13.

*(E)-4-(di(1H-Indol-3-yl)methyl)-N-(4-chlorobenzylidene)aniline* (**23**). Light orange solid; Yield 89%; m.p. 131–133 °C; IR cm^−1^: 3383, 1679, 1584, 1367, 1090; ^1^H-NMR (DMSO-*d*_6_): *δ* 5.87 (s, 1H), 6.84–6.89 (m, 4H), 6.98-7.06 (m, 4H), 7.21 (d, *J =* 8 Hz, 2H), 7.30 (d, *J =* 8.0 Hz, 2H), 7.36 (d, *J =* 8.0 Hz, 2H), 7.40 (d, *J =* 8.0 Hz, 2H), 7.93 (d, *J =* 8.5 Hz, 2H), 8.64 (s, 1H), 10.83 (s, 2H); ^13^C-NMR (MeOD-*d*_4_): *δ* 39.88, 110.68, 110.68, 110.83, 118.11, 118.11, 118.55, 118.55, 119.11, 119.11, 119.22, 119.22, 120.39, 120.39, 120.90, 123.28, 123.28, 123.42, 126.97, 126.97, 128.64, 129.08, 129.08, 129.33, 129.33, 129.76, 129.76, 137.10, 137.10, 159.08. EI MS *m/z* (% rel. abund.): 459.10 (41.0), 337.10 (100.0, M), 245.09 (52.2), 117.08(3.2). Anal. Calcd for C_30_H_22_ClN_3_: C, 78.34; H, 4.82; Cl, 7.71; N, 9.14. Found: C, 78.36; H, 4.80; Cl, 7.74; N, 9.15. 

*(E)-4-(di(1H-Indol-3-yl)methyl)-N-(2-nitrobenzylidene)aniline* (**24**). Dark yellow solid; Yield 83%; m.p. 124–126 °C; IR cm^−1^: 3379, 1664, 1576, 1361, 1340; ^1^H-NMR (DMSO-*d*_6_): *δ* 5.88 (s, 1H), 6.87–6.90 (m, 4H), 7.05 (t, *J =* 7.0 Hz, 2H), 7.23 (d, *J =* 8.5 Hz, 2H), 7.31 (d, *J =* 8.0 Hz, 2H), 7.36 (d, *J =* 8.0 Hz, 2H), 7.43 (d, *J =* 8.5 Hz, 2H), 7.76 (t, *J =* 9.0 Hz, 1H), 7.86 (t, *J =* 7.5 Hz, 1H), 8.10 (d, *J =* 8.0 Hz, 2H), 8.16 (d, *J =* 8.0 Hz, 2H), 8.88 (s, 1H), 10.84 (s, 2H); ^13^C-NMR (MeOD-*d*_4_,): *δ* 39.87, 110.68, 110.68, 110.81, 115.52, 117.87, 118.08, 118.08, 118.51, 118.51, 119.05, 119.05, 120.40, 120.40, 120.70, 120.70, 120.88, 120.88, 123.41, 123.41, 124.37, 126.96, 126.96, 127.48, 129.10, 129.38, 129.38, 137.10, 143.81, 153.30. EI MS *m/z* (% rel. abund.): 470.10 (61.1), 337.12 (100.0, M), 245.07 (73.4), 117.09 (3.4). Anal. Calcd for C_30_H_22_N_4_O_2_: C, 76.58; H, 4.71; N, 11.91; O, 6.80. Found: C, 76.56; H, 4.73; N, 11.92; O, 6.82.

*(E)-4-(di(1H-Indol-3-yl)methyl)-N-(4-nitrobenzylidene)aniline* (**25**). Dark yellow solid; Yield 80%; m.p. 187–189 °C; IR cm^−1^: 3383, 1665, 1574, 1362, 1342; ^1^H-NMR (DMSO-*d*_6_): *δ* 5.89 (s, 1H), 6.87–6.89 (m, 4H), 7.05 (t, *J =* 7.0 Hz, 2H), 7.30-7.32 (m, 4H), 7.36 (d, *J =* 8.0 Hz, 2H), 7.43 (d, *J =* 8.5 Hz, 2H), 8.17 (d, *J =* 9.0 Hz, 2H), 8.36 (d, *J =* 8.5 Hz, 2H), 8.82 (s, 1H), 10.84 (s, 2H); ^13^C-NMR (DMSO-*d*_6_): *δ* 49.07, 111.94, 111.94, 118.36 118.36, 118.67, 118.67, 119.56, 119.56, 121.37, 121.37, 121.61, 121.61, 124.04, 124.04, 124.48, 124.48, 127.06, 127.06, 129.63, 129.95, 129.95, 137.09, 137.09, 142.19, 142.19, 144.60, 148.71, 149.21, 158.42. EI MS *m/z* (% rel. abund.): 469.90 (100.0, M), 336.99 (2.6), 244.99 (44.3), 117.04 (3.2). Anal. Calcd for C_30_H_22_N_4_O_2_: C, 76.58; H, 4.71; N, 11.91; O, 6.80. Found: C, 76.60; H, 4.68; N, 11.93; O, 6.81.

*(E)-4-(di(1H-Indol-3-yl)methyl)-N-(3,4-dimethoxybenzylidene)aniline* (**26**). Dark red solid; Yield 71%; m.p. 214–216 °C; IR cm^−1^: 3395, 1671, 1583, 1358, 1224, 1012; ^1^H-NMR (DMSO-*d*_6_): *δ* 3.86 (s, 6H), 5.85 (s,1H), 6.85–6.89 (m, 4H), 7.04 (t, *J =* 7.0 Hz, 2H), 7.16 (d, *J =* 8.5 Hz, 2H), 7.30 (d, *J =* 8.0 Hz, 2H), 7.35–7.38 (m, 4H), 7.42 (dd, *J =* 1.5 Hz, 8.5 Hz, 1H), 7.52 (d, *J =* 1.5 Hz, 1H), 8.50 (s, 1H), 10.85 (s, 2H); ^13^C-NMR (MeOD-*d*_4_): *δ* 37.95, 56.08, 56.18, 110.81, 110.81, 113.52, 113.62, 114.12, 118.09, 118.09, 118.64, 118.64, 119.11, 119.11, 120.30, 120.30, 120.88, 120.88, 122.57, 122.57, 122.92, 123.41, 124.71, 124.71, 126.99, 126.99, 129.28, 129.28, 137.09, 137.09, 153.59, 160.75. EI MS *m/z* (% rel. abund.): 485.00 (100.0, M), 337.03 (3.3), 321.04 (11.0), 245.03 (22.2), 117.09 (1.2). Anal. Calcd for C_32_H_27_N_3_O_2_: C, 79.15; H, 5.60; N, 8.65; O, 6.59. Found: C, 79.16; H, 5.63; N, 8.67; O, 6.57.

### 3.4. Bioassay

The antibacterial activity of compounds **3**–**26** was evaluated by using the agar diffusion method [44]. Gentamicin was used as positive control. Bacterial cultures which had been subjected through 24 h incubation were mixed with sterile physiological saline solution (0.85%). Turbidity of all cultures was adjusted to match standard inoculum of 0.5 Mac-Farland scale [~10^6^ CFU/mL]. Petri dishes containing 20 mL of Mueller Hinton Agar (MHA) were used for all bacterial strains. Inocula were swabbed on the surface of solidified media. Whatman No. 1 filter paper discs (6 mm in diameter) impregnated with the test compound (10 µL/disc) were placed on the plates. Plates inoculated with the bacteria were incubated for 24 h at 37 °C. All tests were replicated thrice and the final readings were determined by using average diameter recorded.

## 4. Conclusions

Nickel boride generated through an *in-situ* reaction between sodium borohydride and nickel acetate and found to be a selective as well as mild reducing agent for compound **1**. Nickel boride is capable of converting its nitro group into amino group with very high yield in a shorter reaction time without affecting the main bisindolylmethane skeleton. The antibacterial activity of bisindolylmethane Schiff base derivatives **3**–**26** demonstrated that compounds **19**, **20**, **21**, **22** and **26** specifically inhibit the *Salmonella typhi*, *S. paratyphi A* and *Salmonella paratyphi B* bacterial strains. Further modification of the structure or introduction of other substituents could improve the antibacterial potential of these compounds and help in the discovery of new and antibacterial agents.

## Supplementary Materials

Supplementary materials can be accessed at: http://www.mdpi.com/1420-3049/19/8/11722/s1.

## Supplementary Files

Supplementary File 1
