# Supplementary material for: Synthesis of Novel Bisindolylmethane Schiff bases and Their Antibacterial Activity
Source: Molecules. 2014 Aug 6;19(8):11722–40. doi: 10.3390/molecules190811722 (PMC6271760; doi:10.3390/molecules190811722)
Supplement: Supplementary File 1 [file molecules-19-11722-s001.pdf]

Supplementary Materials

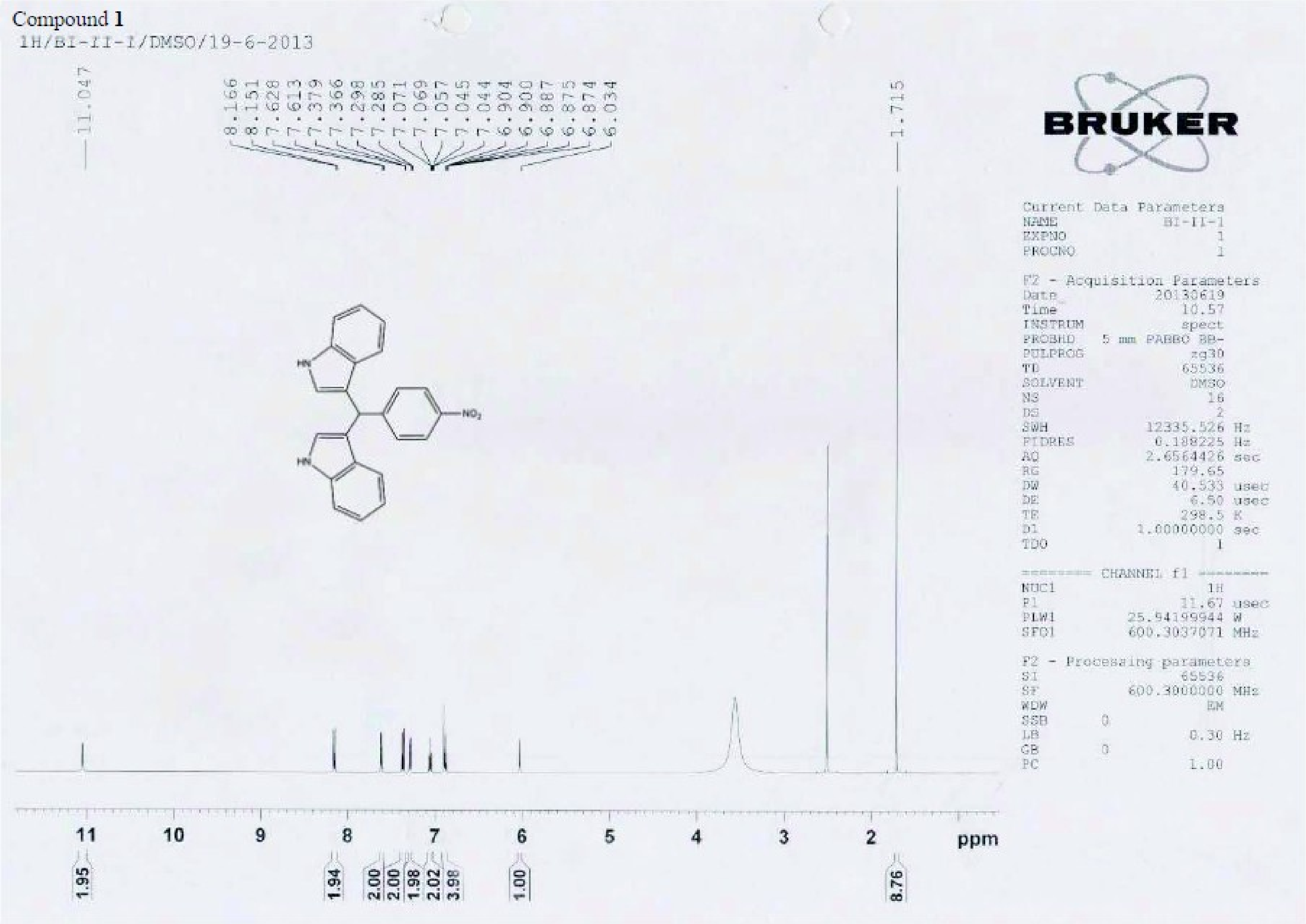

Compound 2

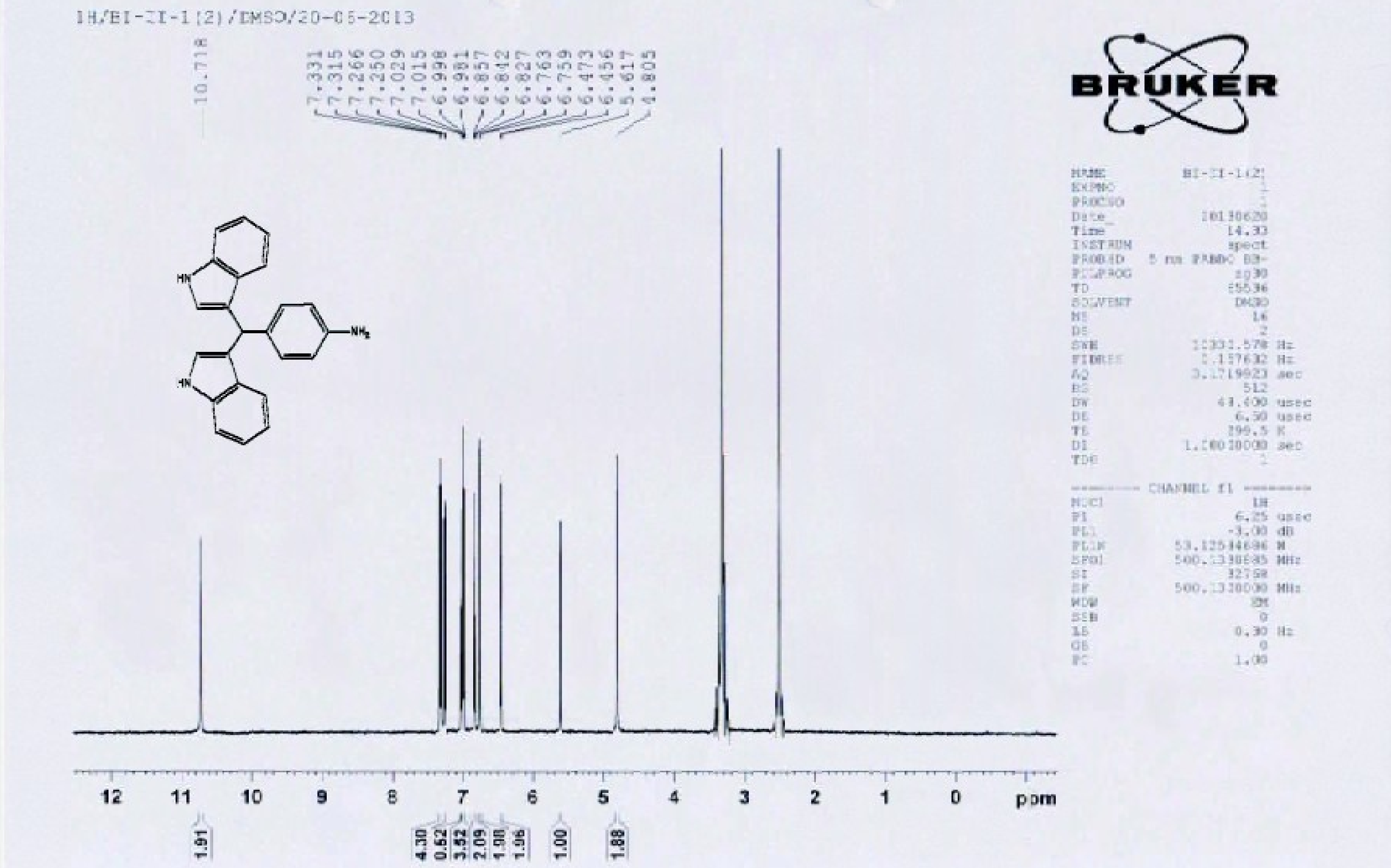
1H/BI-ZI-1(2)/DMSO/20-06-2013
10.718
7.331
7.315
7.266
7.250
7.029
7.015
6.998
6.981
6.857
6.842
6.827
6.763
6.759
6.473
6.456
5.617
4.805
BRUKER
HN
HN
NH2
CHANNEL f1
12
11
10
9
8
7
6
5
4
3
2
1
0
ppm
1.91
4.30
0.52
3.52
2.09
1.98
1.96
1.00
1.88

Compound **2**

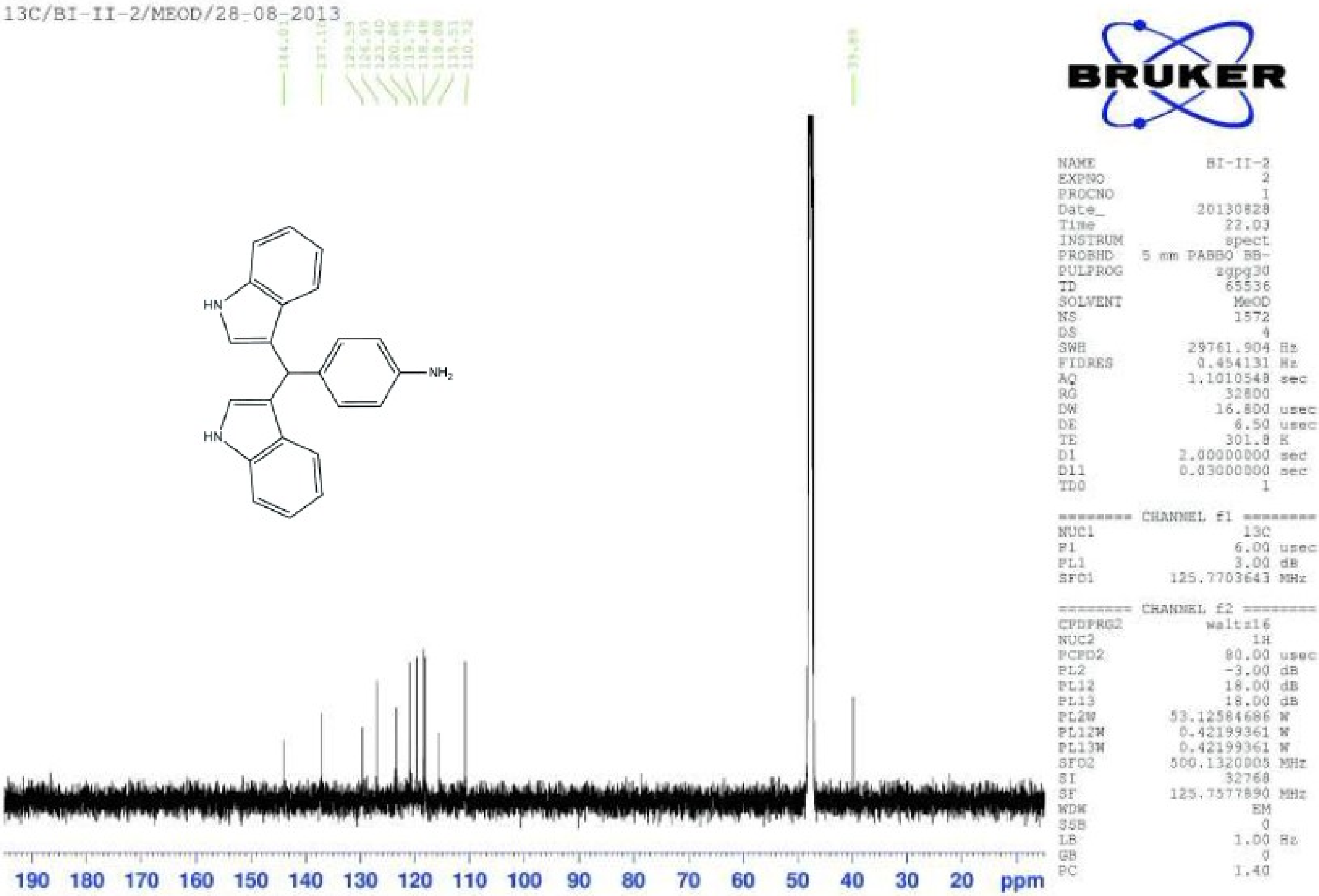
13C/BI-II-2/MEOD/28-08-2013
HN
HN
NH2
190 180 170 160 150 140 130 120 110 100 90 80 70 60 50 40 30 20 ppm
BRUKER
NAME BI-II-2
EXPNO 2
PROCNO 1
Date_ 20130828
Time 22.03
INSTRUM spect
PROBHD 5 mm PABBO BB-
PULPROG zgpg30
TD 65536
SOLVENT MeOD
NS 1572
DS 4
SWH 29761.904 Hz
FIDRES 0.454131 Hz
AQ 1.1010548 sec
RG 32800
DW 16.800 usec
DE 6.50 usec
TE 301.8 K
D1 2.00000000 sec
D11 0.03000000 sec
TD0 1
======== CHANNEL f1 ========
NUC1 13C
P1 6.00 usec
PL1 3.00 dB
SFO1 125.7703643 MHz
======== CHANNEL f2 ========
CPDPRG2 waltz16
NUC2 1H
PCPD2 80.00 usec
PL2 -3.00 dB
PL12 18.00 dB
PL13 18.00 dB
PL2W 53.12584686 W
PL12W 0.42199361 W
PL13W 0.42199361 W
SFO2 500.1320005 MHz
SI 32768
SF 125.7577890 MHz
WDW EM
SSB 0
LB 1.00 Hz
GB 0
PC 1.40

Compound **3**

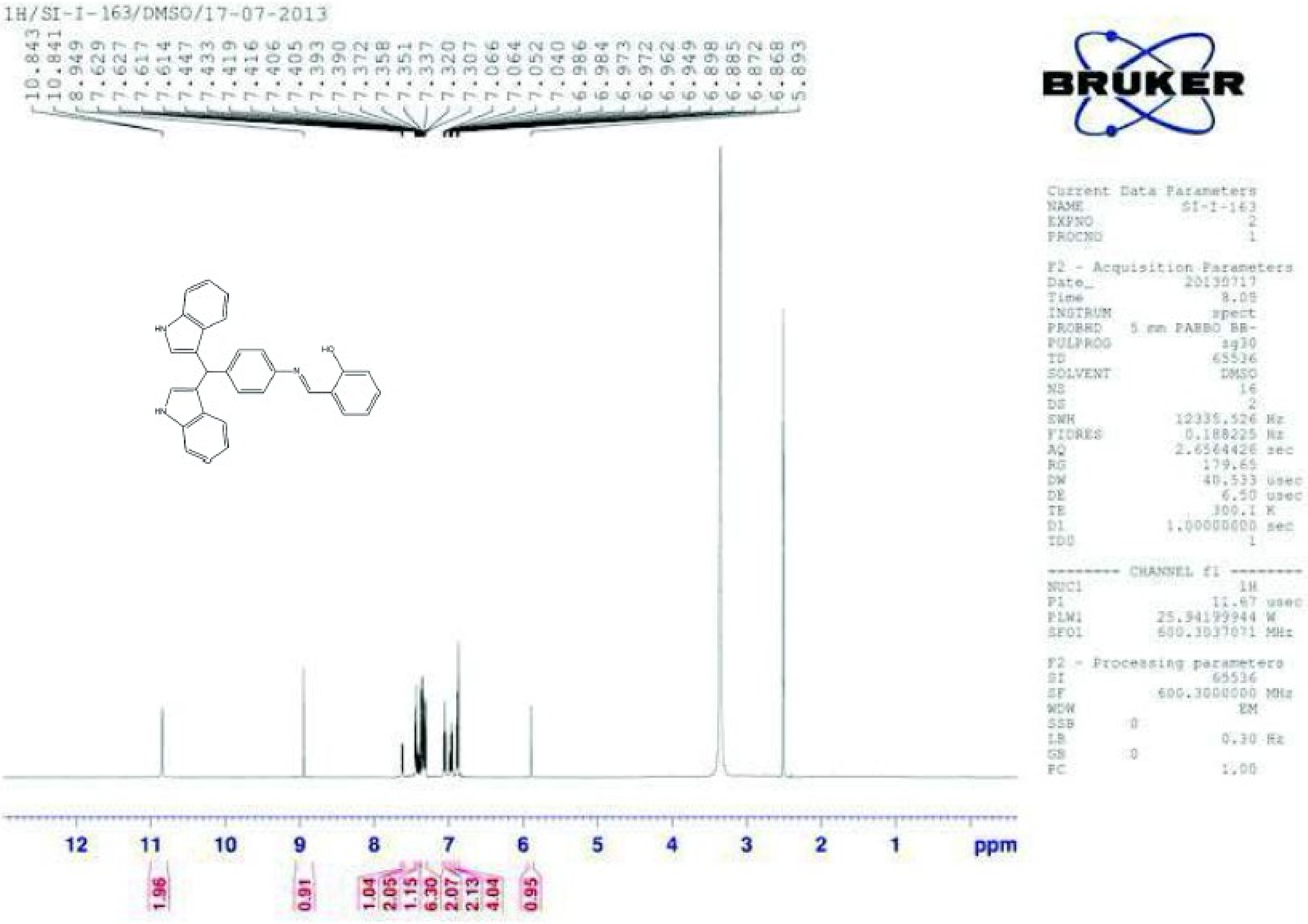
1H/SI-I-163/DMSO/17-07-2013
10.843
10.841
8.949
7.629
7.627
7.617
7.614
7.447
7.433
7.419
7.416
7.406
7.405
7.393
7.390
7.372
7.358
7.351
7.337
7.320
7.307
7.066
7.064
7.052
7.040
6.986
6.984
6.973
6.972
6.962
6.949
6.898
6.885
6.872
6.868
5.893
HN
HN
HO
N
BRUKER
Current Data Parameters
NAME SI-I-163
EXPNO 2
PROCNO 1
F2 - Acquisition Parameters
Date_ 20130717
Time 8.08
INSTRUM spect
PROBHD 5 mm PABBO BB-
PULPROG zg30
TD 65536
SOLVENT DMSO
NS 16
DS 2
SWH 12335.526 Hz
FIDRES 0.188225 Hz
AQ 2.6564426 sec
RG 179.65
DW 40.533 usec
DE 6.50 usec
TE 300.1 K
D1 1.00000000 sec
TD0 1
======== CHANNEL f1 ========
NUC1 1H
P1 11.67 usec
PLW1 25.94199944 W
SFO1 600.3037071 MHz
F2 - Processing parameters
SI 65536
SF 600.3000000 MHz
WDW EM
SSB 0
LB 0.30 Hz
GB 0
PC 1.00
12
11
10
9
8
7
6
5
4
3
2
1
ppm
1.96
0.91
1.04
2.05
1.15
6.30
2.07
2.13
4.04
0.95

Compound **3**

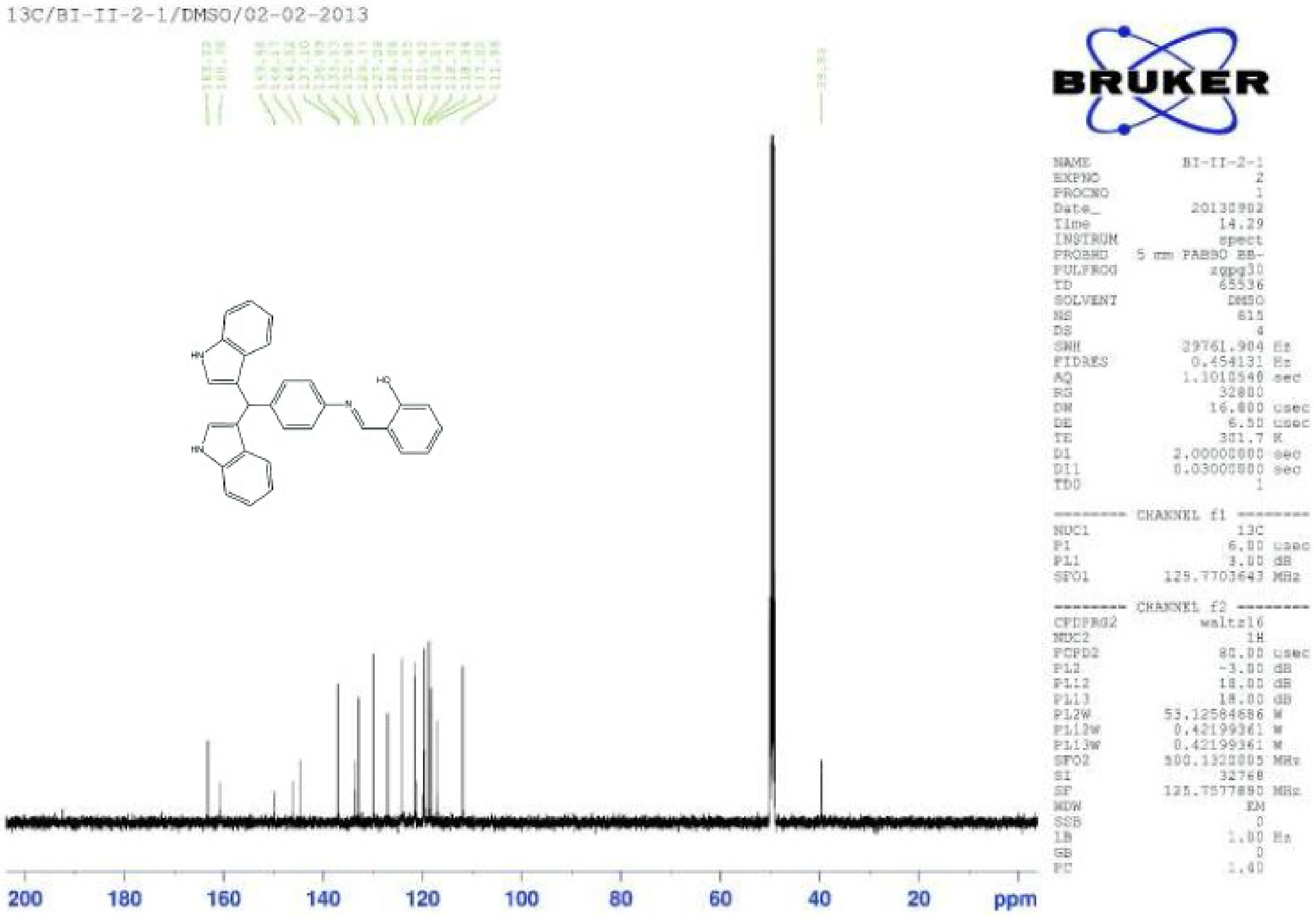
13C/BI-II-2-1/DMSO/02-02-2013
BRUKER
NAME BI-II-2-1
EXPNO 2
PROCNO 1
Date_ 20130202
Time 14.29
INSTRUM spect
PROBHD 5 mm PABBO BB-
PULPROG zgpg30
TD 65536
SOLVENT DMSO
NS 615
DS 4
SWH 29761.904 Hz
FIDRES 0.454131 Hz
AQ 1.1010540 sec
RG 32800
DW 16.800 usec
DE 6.50 usec
TE 301.7 K
D1 2.00000000 sec
D11 0.03000000 sec
TD0 1
======== CHANNEL f1 ========
NUC1 13C
P1 6.00 usec
PL1 3.00 dB
SFO1 125.7703643 MHz
======== CHANNEL f2 ========
CPDPRG2 waltz16
NUC2 1H
PCPD2 80.00 usec
PL2 -3.00 dB
PL12 18.00 dB
PL13 18.00 dB
PL2W 53.12584686 W
PL12W 0.42199361 W
PL13W 0.42199361 W
SFO2 500.1320005 MHz
SI 32768
SF 125.7577890 MHz
WDW EM
SSB 0
LB 1.00 Hz
GB 0
PC 1.40
200
180
160
140
120
100
80
60
40
20
ppm

Compound **4**

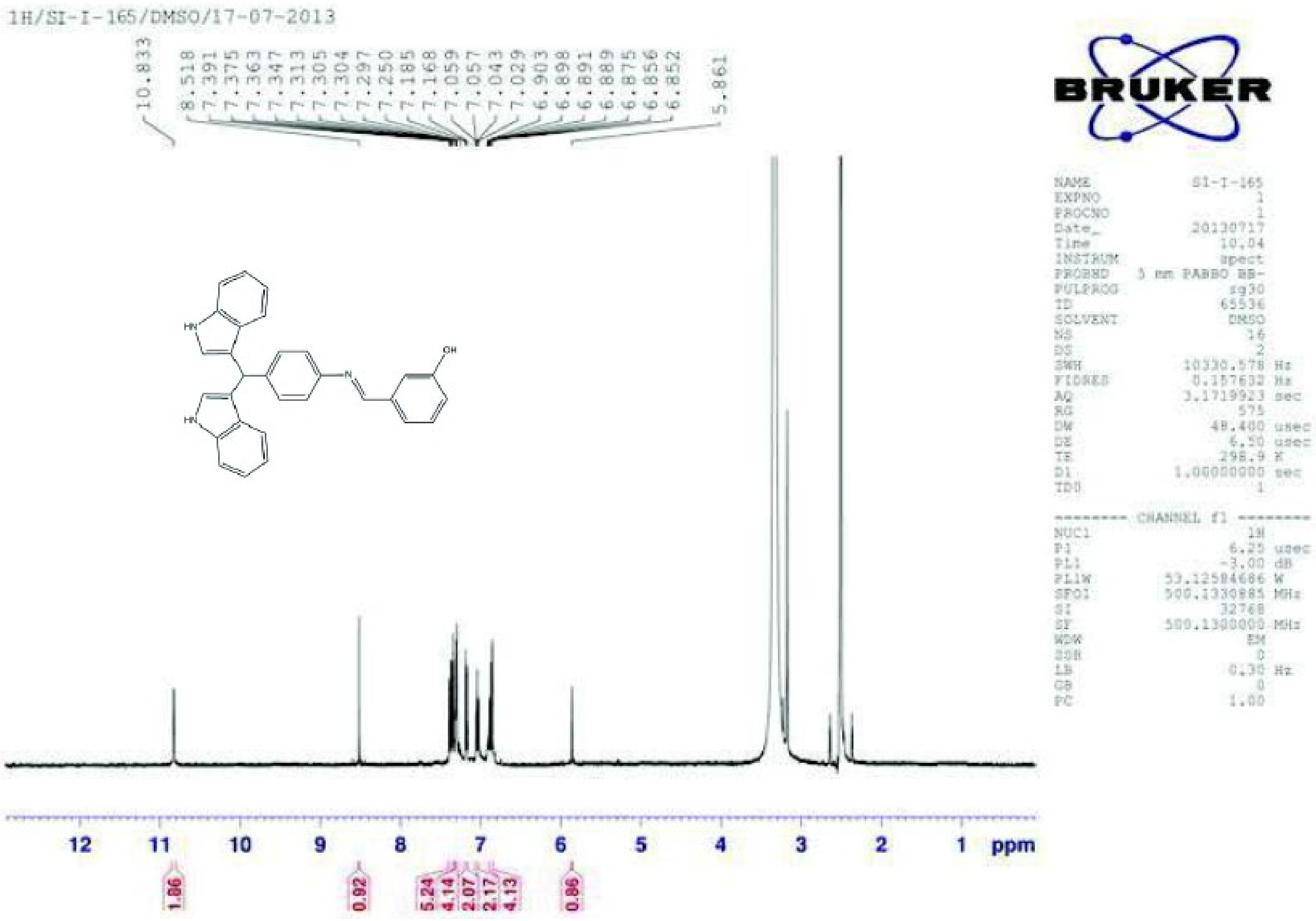
1H/SI-I-165/DMSO/17-07-2013
10.833
8.518
7.391
7.375
7.363
7.347
7.313
7.305
7.304
7.297
7.250
7.185
7.168
7.059
7.057
7.043
7.029
6.903
6.898
6.891
6.889
6.875
6.856
6.852
5.861
BRUKER
HN
HN
N
OH
NAME SI-I-165
EXPNO 1
PROCNO 1
Date_ 20130717
Time 10.04
INSTRUM spect
PROBHD 5 mm PABBO BB-
PULPROG zg30
TD 65536
SOLVENT DMSO
NS 16
DS 2
SWH 10330.578 Hz
FIDRES 0.157632 Hz
AQ 3.1719923 sec
RG 575
DW 48.400 usec
DE 6.50 usec
TE 298.9 K
D1 1.00000000 sec
TD0 1
======== CHANNEL f1 ========
NUC1 1H
P1 6.25 usec
PL1 -3.00 dB
PL1W 53.12584686 W
SFO1 500.1330885 MHz
SI 32768
SF 500.1300000 MHz
WDW EM
SSB 0
LB 0.30 Hz
GB 0
PC 1.00
12
11
10
9
8
7
6
5
4
3
2
1
ppm
1.96
0.92
5.24
4.14
2.07
2.17
4.13
0.86

Compound **4**

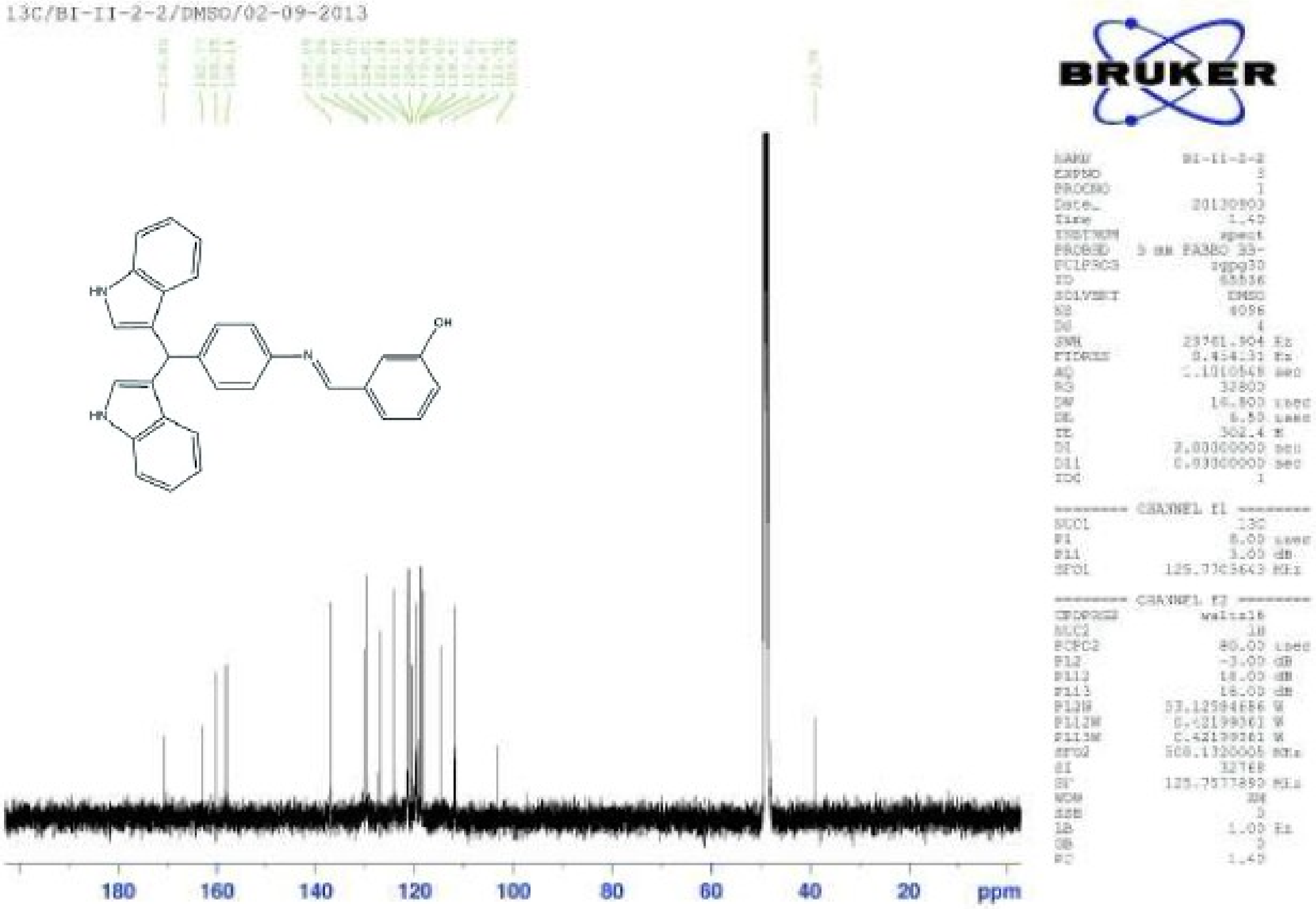
13C/BI-II-2-2/DMSO/02-09-2013
BRUKER
HN
HN
N
OH
180
160
140
120
100
80
60
40
20
ppm

Compound 5

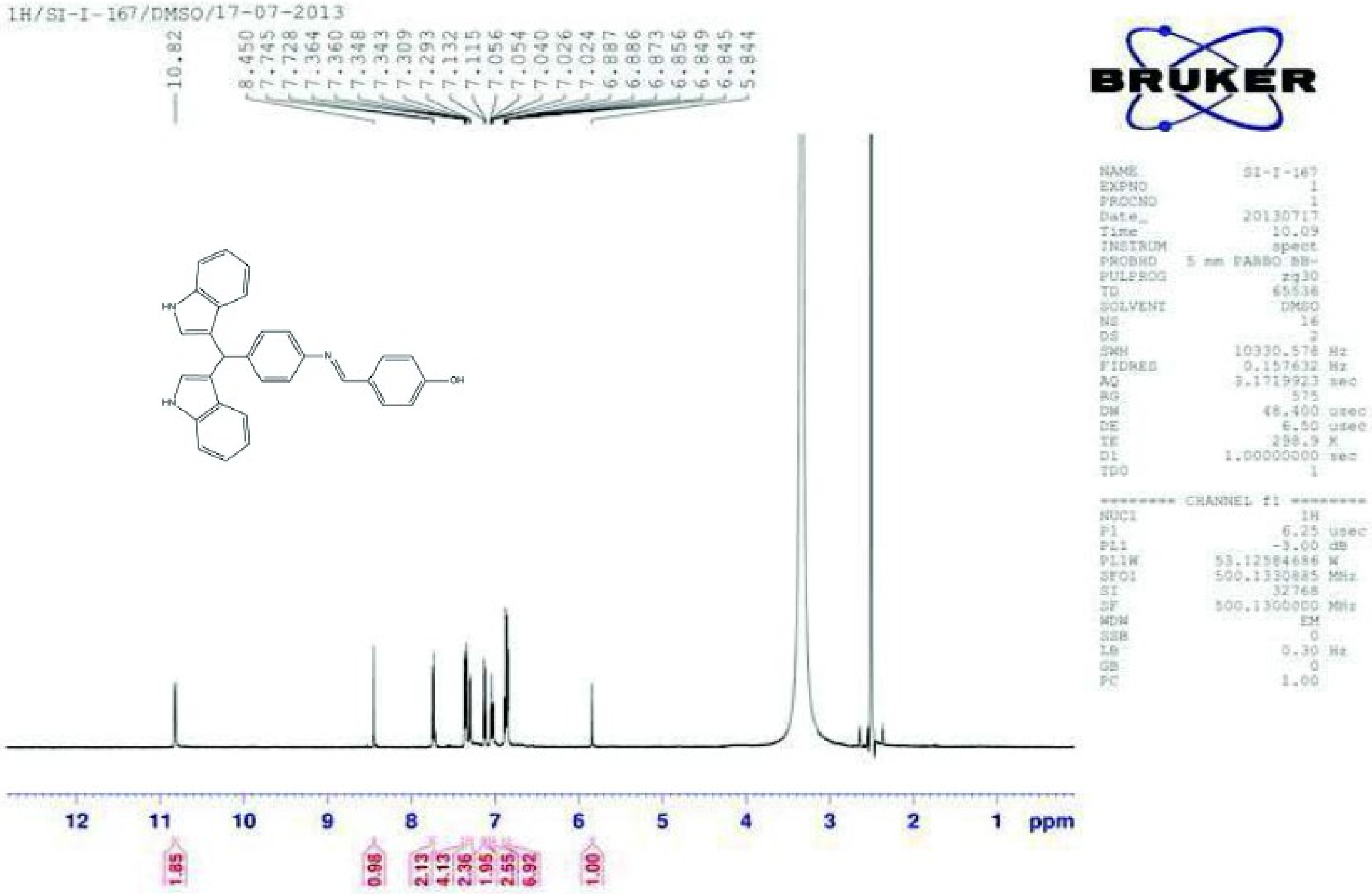
1H/SI-I-167/DMSO/17-07-2013
10.82
8.450
7.745
7.728
7.364
7.360
7.348
7.343
7.309
7.293
7.132
7.115
7.056
7.054
7.040
7.026
7.024
6.887
6.886
6.873
6.856
6.849
6.845
5.844
HN
HN
N
OH
BRUKER
NAME SI-I-167
EXPNO 1
PROCNO 1
Date_ 20130717
Time 10.09
INSTRUM spect
PROBHD 5 mm PABBO BB-
PULPROG zg30
TD 65536
SOLVENT DMSO
NS 16
DS 2
SWH 10330.578 Hz
FIDRES 0.157632 Hz
AQ 3.1719923 sec
RG 575
DW 48.400 usec
DE 6.50 usec
TE 298.9 K
D1 1.00000000 sec
TD0 1
======== CHANNEL f1 ========
NUC1 1H
P1 6.25 usec
PL1 -3.00 dB
PL1W 53.12584686 W
SFO1 500.1330885 MHz
SI 32768
SF 500.1300000 MHz
WDW EM
SSB 0
LB 0.30 Hz
GB 0
PC 1.00
12
11
10
9
8
7
6
5
4
3
2
1
ppm
1.85
0.98
2.13
4.13
2.36
1.95
2.55
6.92
1.00

Compound 5

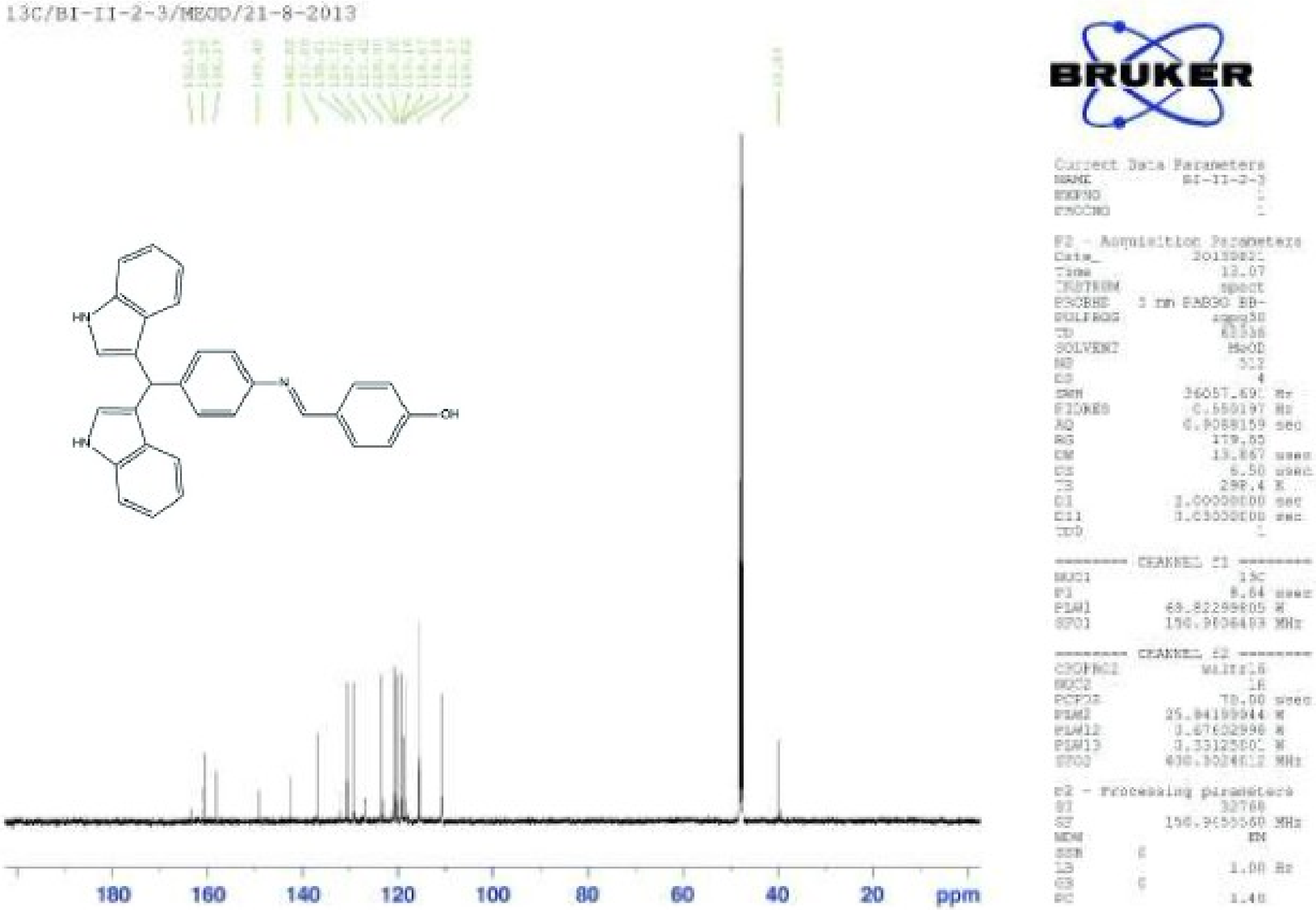
13C/BI-II-2-3/MEOD/21-8-2013
HN
HN
N
OH
BRUKER
Current Data Parameters
NAME BI-II-2-3
F2 - Acquisition Parameters
SOLVENT MeOD
CHANNEL f1
13C
CHANNEL f2
1H
F2 - Processing parameters
180
160
140
120
100
80
60
40
20
ppm

Compound 6

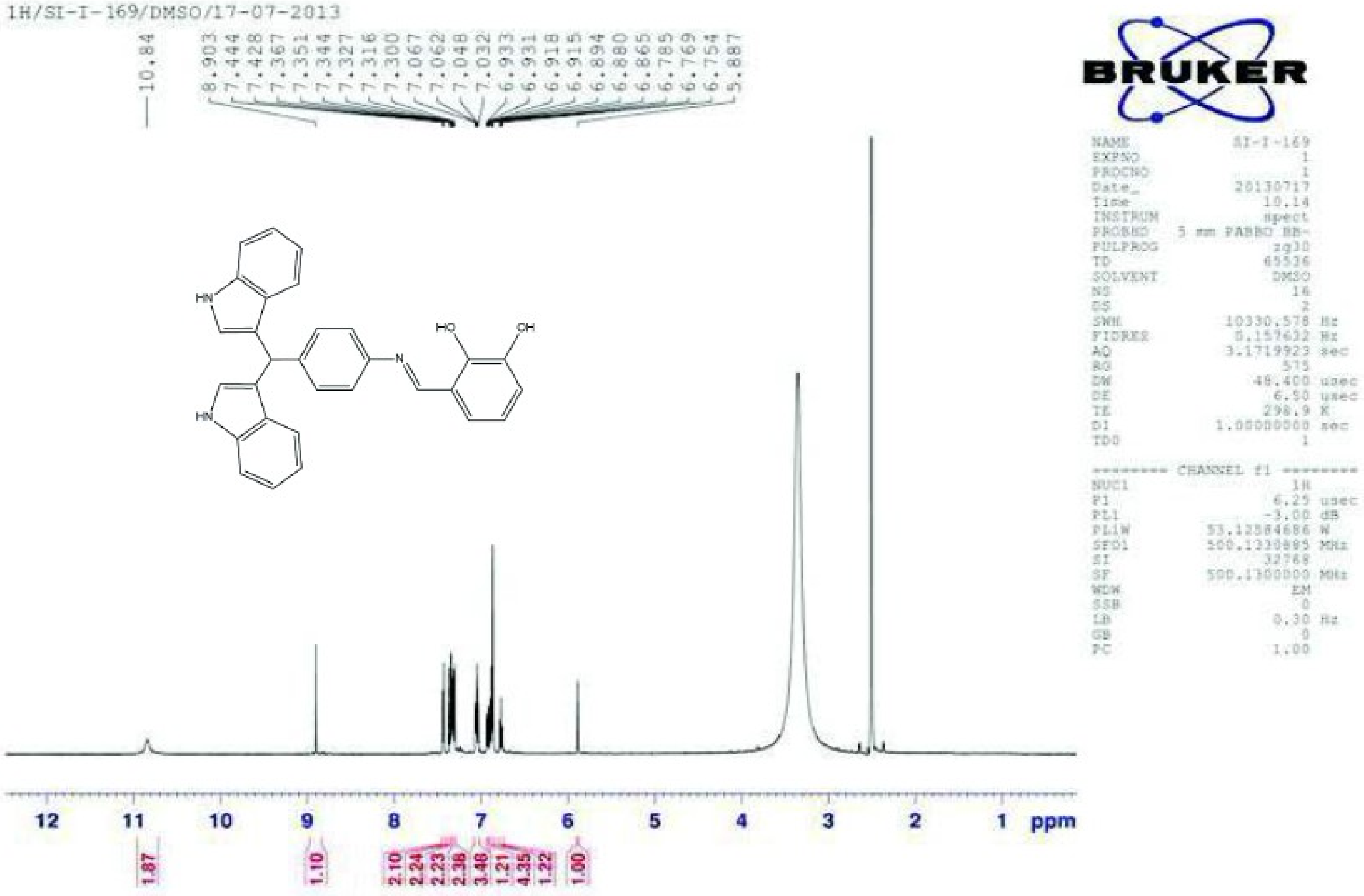
1H/SI-I-169/DMSO/17-07-2013
10.84
8.903 7.444 7.428 7.367 7.351 7.344 7.327 7.316 7.300 7.067 7.062 7.048 7.032 6.933 6.931 6.918 6.915 6.894 6.880 6.865 6.785 6.769 6.754 5.887
HN
HN
HO
OH
N
BRUKER
NAME SI-I-169
EXPNO 1
PROCNO 1
Date_ 20130717
Time 10.14
INSTRUM spect
PROBHD 5 mm PABBO BB-
PULPROG zg30
TD 65536
SOLVENT DMSO
NS 16
DS 2
SWH 10330.578 Hz
FIDRES 0.157632 Hz
AQ 3.1719923 sec
RG 575
DW 48.400 usec
DE 6.50 usec
TE 298.9 K
D1 1.00000000 sec
TD0 1
======== CHANNEL f1 ========
NUC1 1H
P1 6.25 usec
PL1 -3.00 dB
PL1W 53.12584686 W
SFO1 500.1330885 MHz
SI 32768
SF 500.1300000 MHz
WDW EM
SSB 0
LB 0.30 Hz
GB 0
PC 1.00
12 11 10 9 8 7 6 5 4 3 2 1 ppm
1.87 1.10 2.10 2.24 2.23 2.38 3.48 1.21 4.35 1.22 1.00

Compound 6

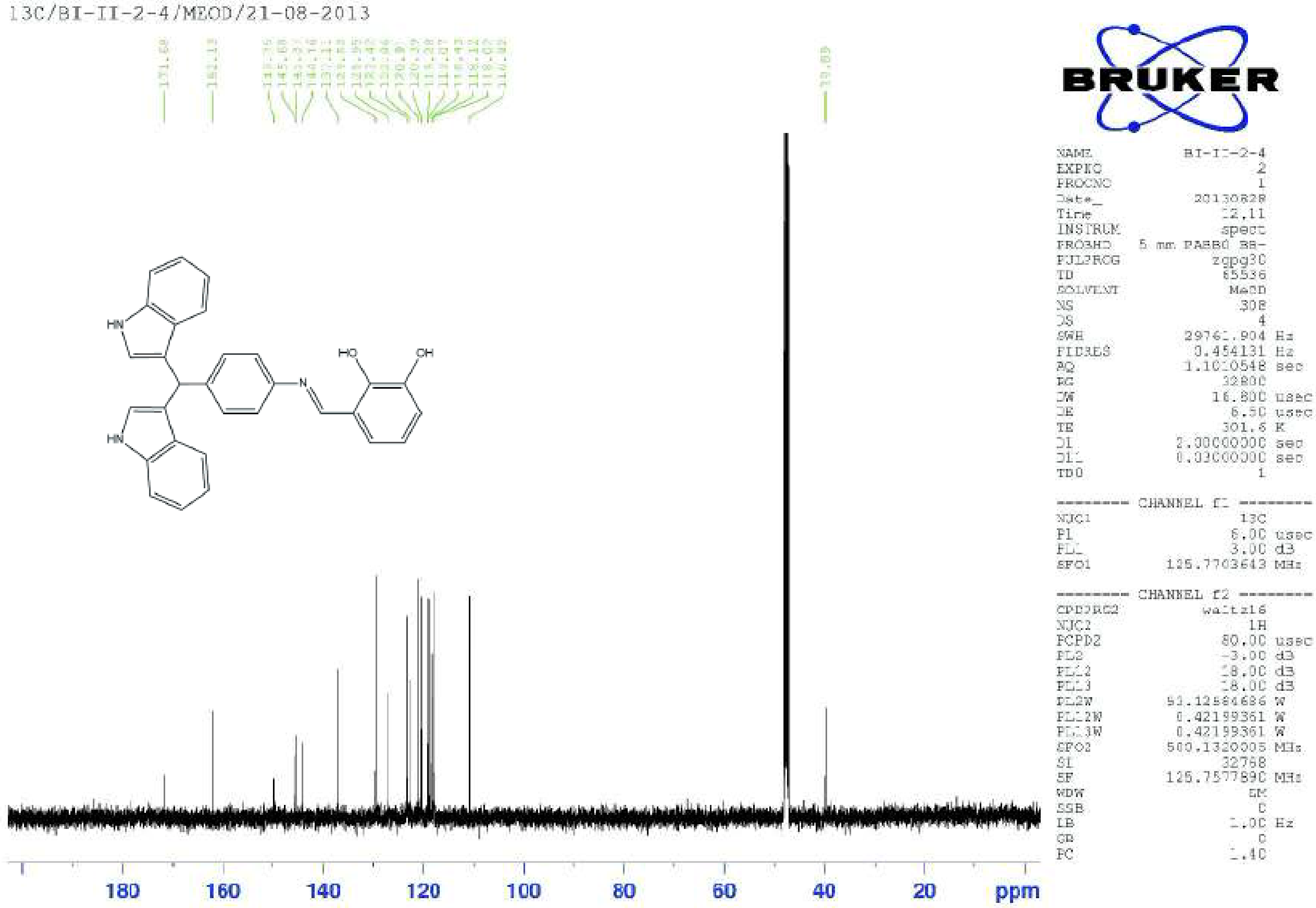
13C/BI-II-2-4/MEOD/21-08-2013
HN
HN
HO
OH
N
BRUKER
NAME BI-II-2-4
EXPNO 2
PROCNO 1
TD 65536
NS 308
DS 4
TE 301.6 K
TD0 1
CHANNEL f1
NUC1 13C
P1 6.00 usec
SFO1 125.7703643 MHz
CHANNEL f2
NUC2 1H
PCPD2 80.00 usec
SFO2 500.1320005 MHz
SI 32768
SF 125.7577890 MHz
WDW EM
SSB 0
GB 0
PC 1.40
180
160
140
120
100
80
60
40
20
ppm

Compound 7

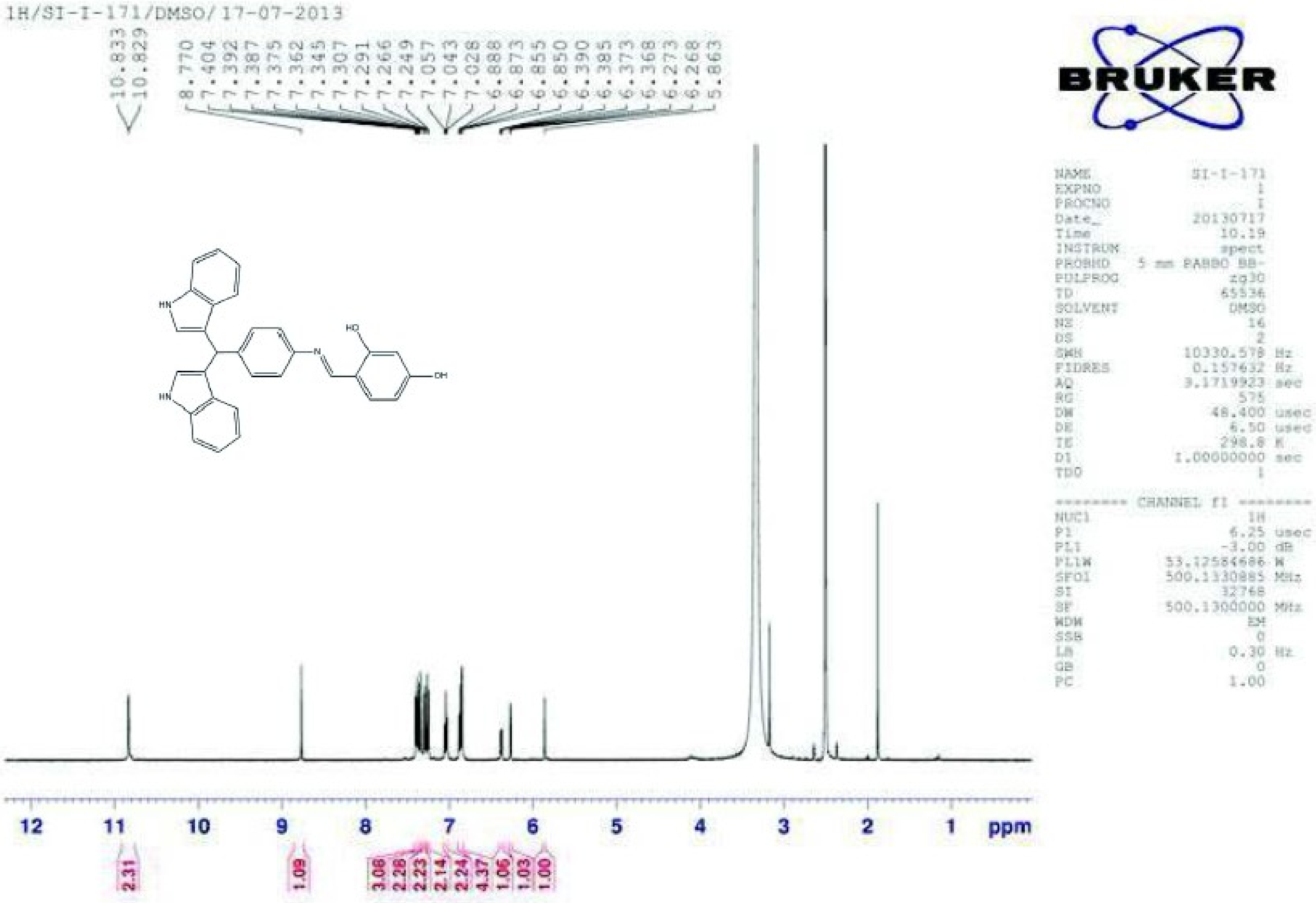
1H/SI-I-171/DMSO/ 17-07-2013
10.833
10.829
8.770
7.404
7.392
7.387
7.375
7.362
7.345
7.307
7.291
7.266
7.249
7.057
7.043
7.028
6.888
6.873
6.855
6.850
6.390
6.385
6.373
6.368
6.273
6.268
5.863
HN
HN
HO
N
OH
12
11
10
9
8
7
6
5
4
3
2
1
ppm
2.31
1.09
3.06
2.28
2.23
2.14
2.24
4.37
1.06
1.03
1.00
BRUKER
NAME SI-I-171
EXPNO 1
PROCNO 1
Date_ 20130717
Time 10.19
INSTRUM spect
PROBHD 5 mm PABBO BB-
PULPROG zg30
TD 65536
SOLVENT DMSO
NS 16
DS 2
SWH 10330.578 Hz
FIDRES 0.157632 Hz
AQ 3.1719923 sec
RG 575
DW 48.400 usec
DE 6.50 usec
TE 298.8 K
D1 1.00000000 sec
TD0 1
======== CHANNEL f1 ========
NUC1 1H
P1 6.25 usec
PL1 -3.00 dB
PL1W 53.12584686 W
SFO1 500.1330885 MHz
SI 32768
SF 500.1300000 MHz
WDW EM
SSB 0
LB 0.30 Hz
GB 0
PC 1.00

Compound 7

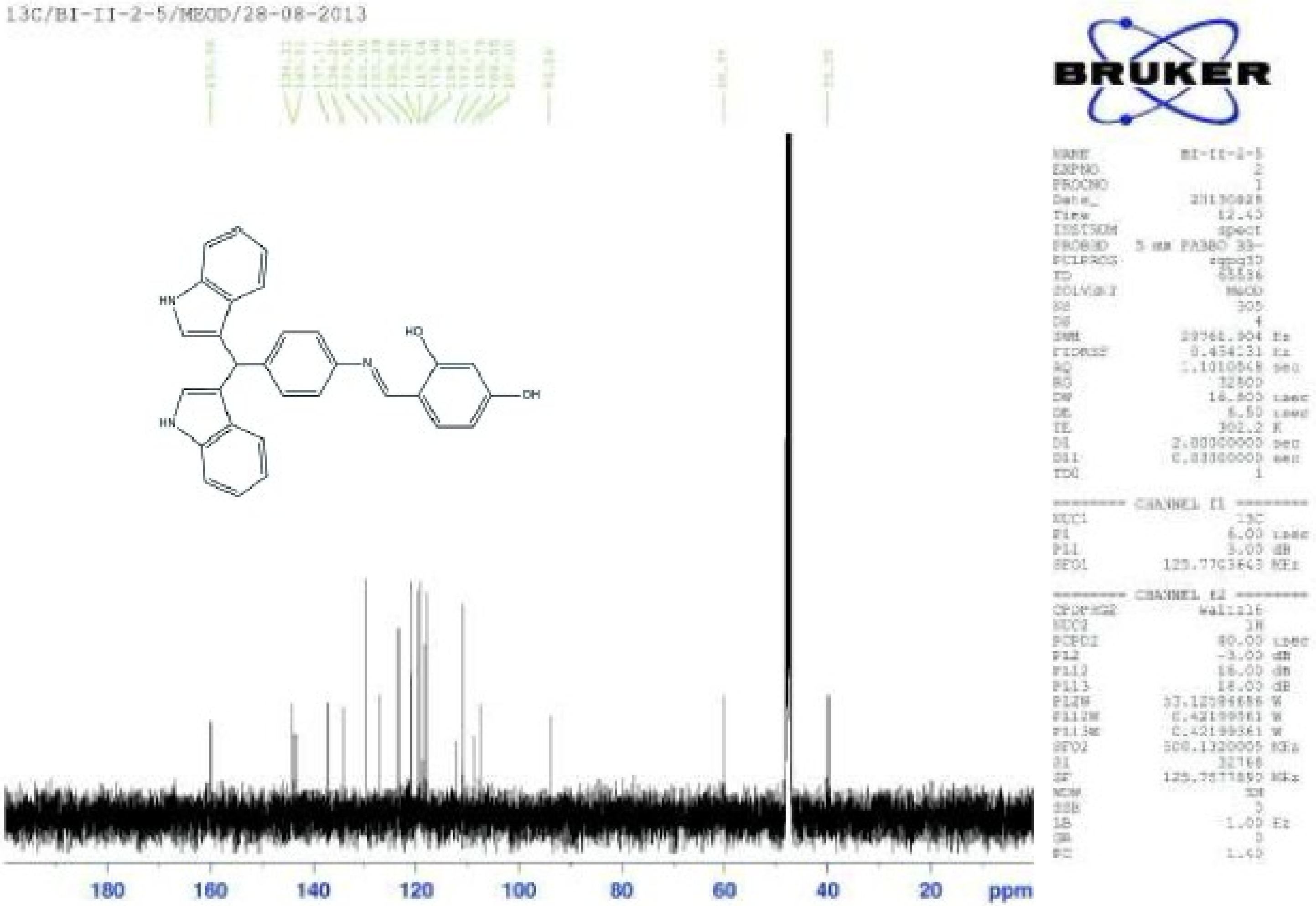
13C/BI-II-2-5/MEOD/28-08-2013
BRUKER
HN
HN
HO
N
OH
180
160
140
120
100
80
60
40
20
ppm

Compound 8

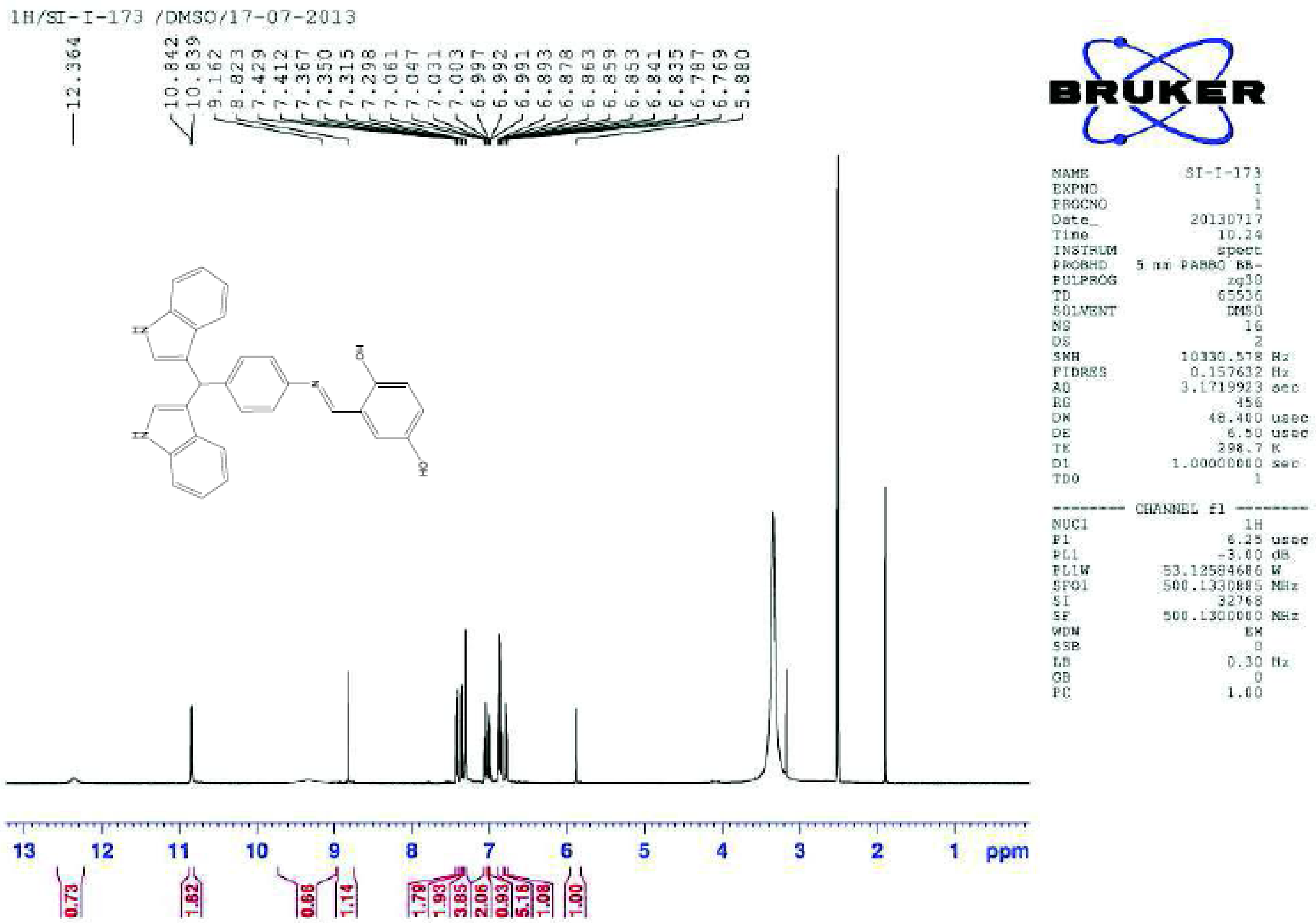
1H/SI-I-173 /DMSO/17-07-2013
BRUKER
NAME SI-I-173
EXPNO 1
PROCNO 1
Date_ 20130717
Time 10.24
INSTRUM spect
PROBHD 5 mm PABBO BB-
PULPROG zg30
TD 65536
SOLVENT DMSO
NS 16
DS 2
SWH 10330.578 Hz
FIDRES 0.157632 Hz
AQ 3.1719923 sec
RG 456
DW 48.400 usec
DE 6.50 usec
TE 298.7 K
D1 1.00000000 sec
TD0 1
CHANNEL f1
NUC1 1H
P1 6.25 usec
PL1 -3.00 dB
PL1W 53.12584686 W
SFO1 500.1330885 MHz
SI 32768
SF 500.1300000 MHz
WDW EM
SSB 0
LB 0.30 Hz
GB 0
PC 1.00
ppm

Compound 8

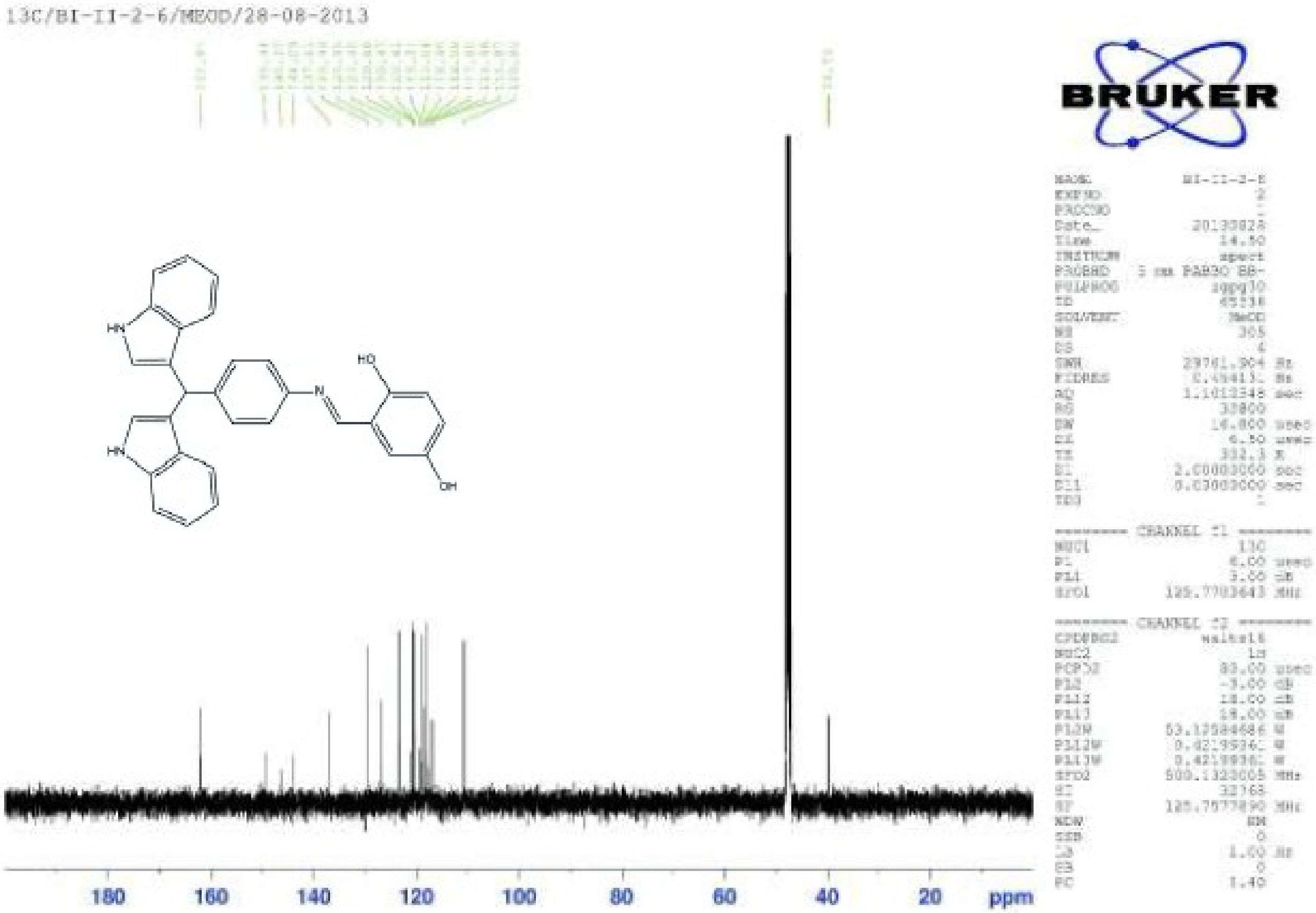
13C/BI-II-2-6/MEOD/28-08-2013
HN
HN
HO
N
OH
BRUKER
180
160
140
120
100
80
60
40
20
ppm

Compound 9

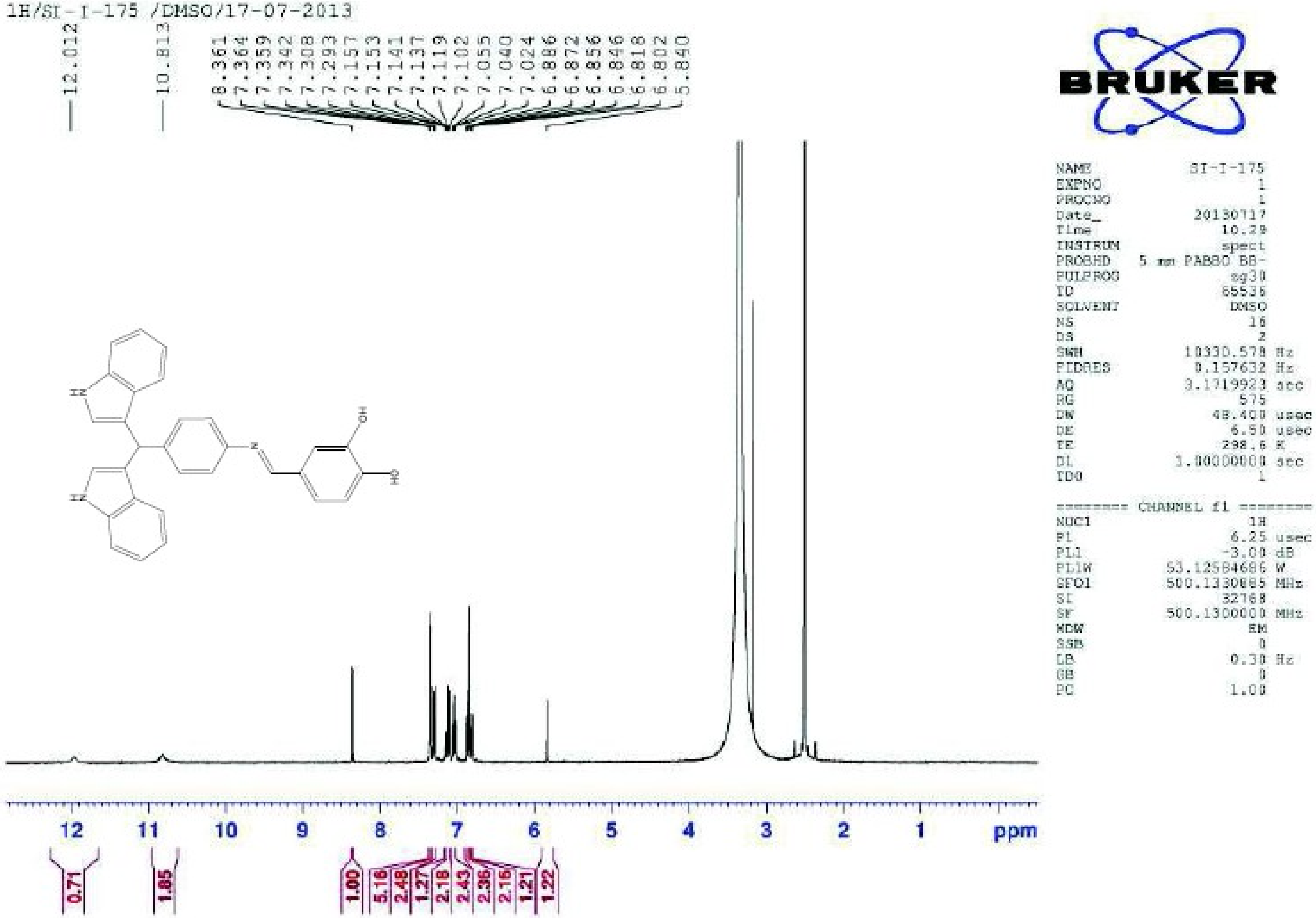

1H/SI-I-175 /DMSO/17-07-2013
12.012
10.813
8.361
7.364
7.359
7.342
7.308
7.293
7.157
7.153
7.141
7.137
7.119
7.102
7.055
7.040
7.024
6.886
6.872
6.856
6.846
6.818
6.802
5.840
NAME SI-I-175
EXPNO 1
PROCNO 1
Date_ 20130717
Time 10.29
INSTRUM spect
PROBHD 5 mm PABBO BB-
PULPROG zg30
TD 65536
SOLVENT DMSO
NS 16
DS 2
SWH 10330.578 Hz
FIDRES 0.157632 Hz
AQ 3.1719923 sec
RG 575
DW 48.400 usec
DE 6.50 usec
TE 298.6 K
D1 1.00000000 sec
TD0 1
======== CHANNEL f1 ========
NUC1 1H
P1 6.25 usec
PL1 -3.00 dB
PL1W 53.12584686 W
SFO1 500.1330885 MHz
SI 32768
SF 500.1300000 MHz
WDW EM
SSB 0
LB 0.30 Hz
GB 0
PC 1.00
12
11
10
9
8
7
6
5
4
3
2
1
ppm
0.71
1.85
1.00
5.16
2.48
1.27
2.18
2.43
2.36
2.15
1.21
1.22

Compound 9

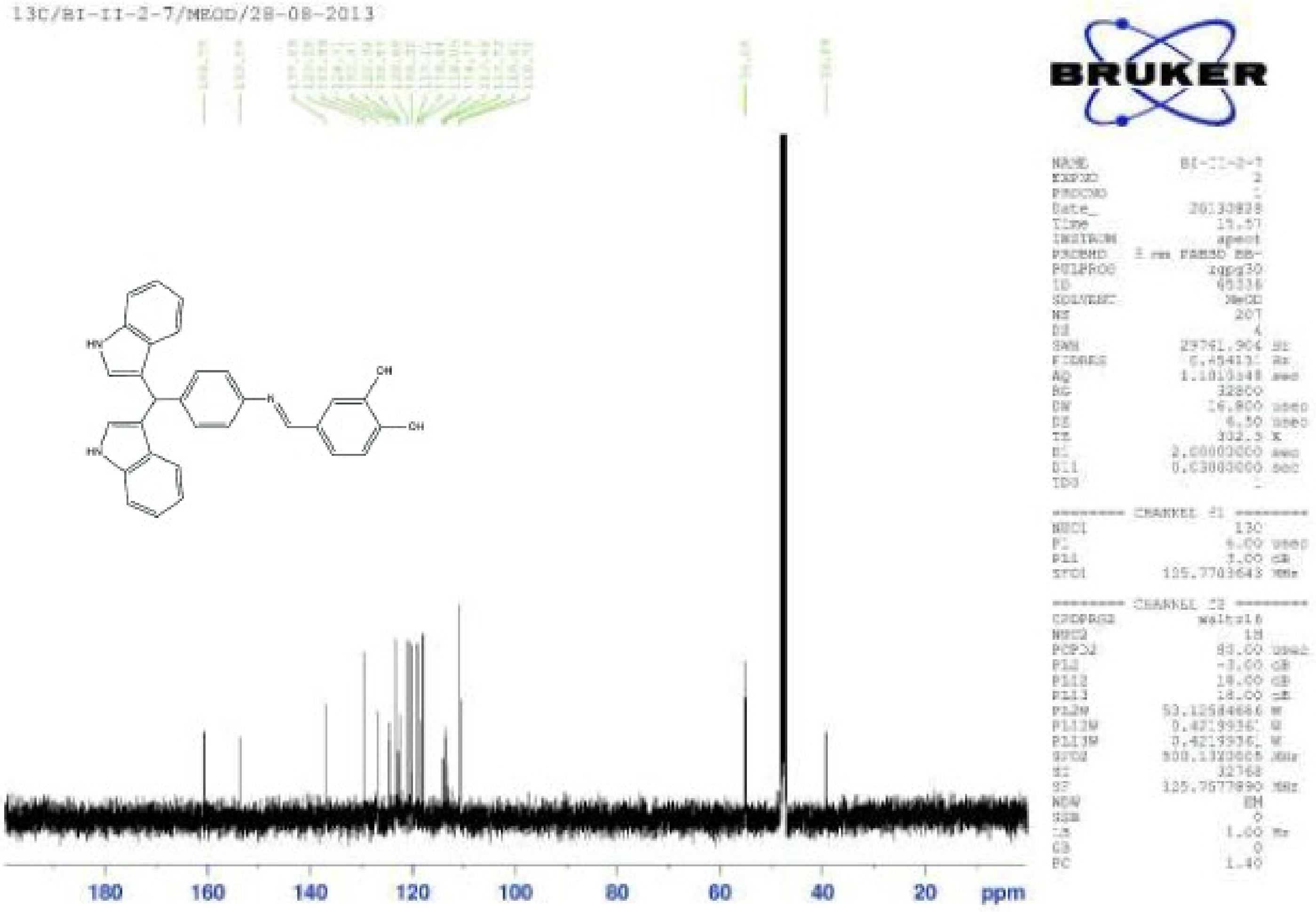
13C/BI-II-2-7/MEOD/28-08-2013
OH
OH
HN
HN
N
180
160
140
120
100
80
60
40
20
ppm

Compound 10

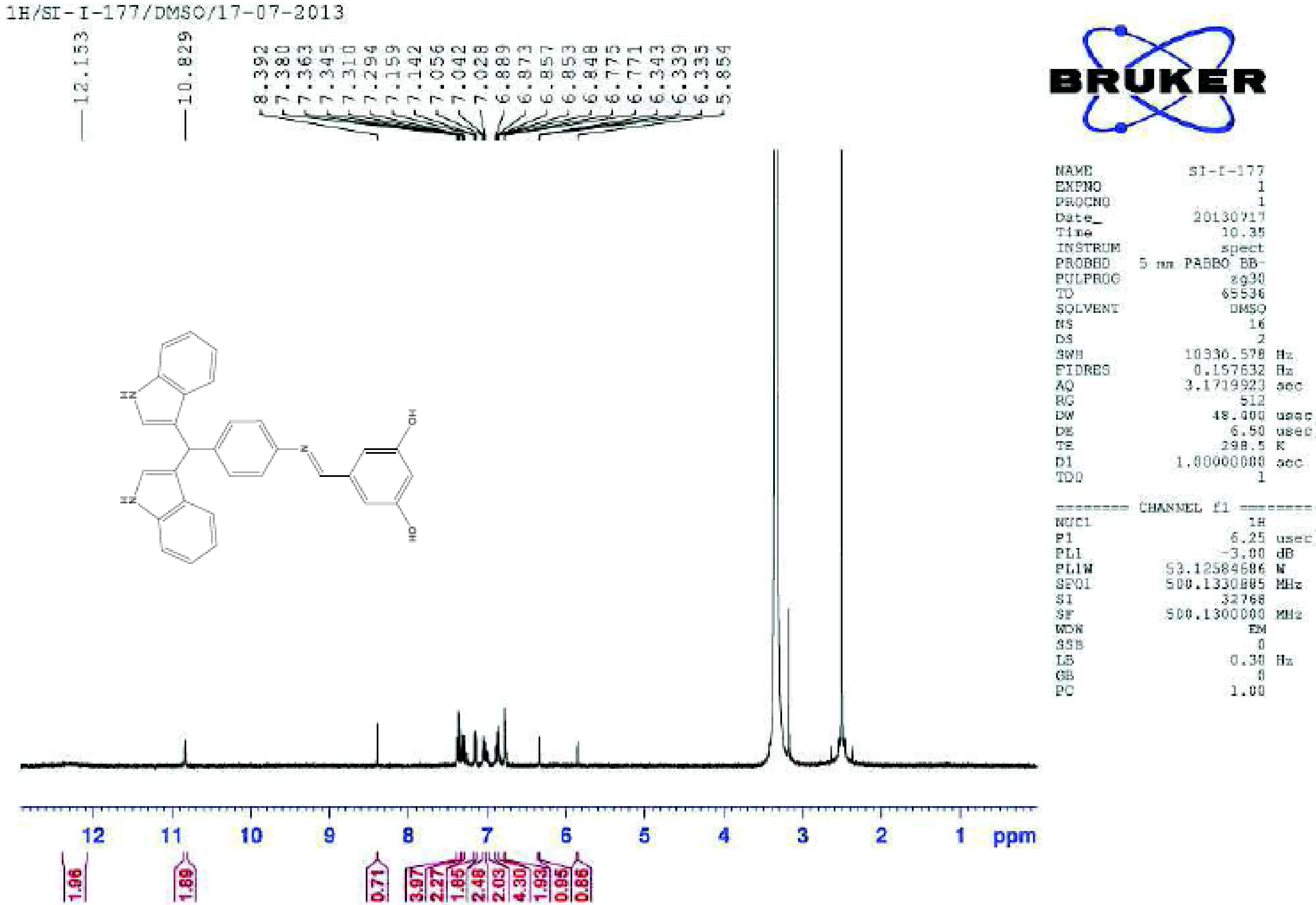
1H/SI-I-177/DMSO/17-07-2013
12.153
10.829
8.392
7.380
7.363
7.345
7.310
7.294
7.159
7.142
7.056
7.042
7.028
6.889
6.873
6.857
6.853
6.848
6.775
6.771
6.343
6.339
6.335
5.854
NAME SI-I-177
EXPNO 1
PROCNO 1
Date_ 20130717
Time 10.35
INSTRUM spect
PROBHD 5 mm PABBO BB-
PULPROG zg30
TD 65536
SOLVENT DMSO
NS 16
DS 2
SWH 10330.578 Hz
FIDRES 0.157632 Hz
AQ 3.1719923 sec
RG 512
DW 48.400 usec
DE 6.50 usec
TE 298.5 K
D1 1.00000000 sec
TD0 1
======== CHANNEL f1 ========
NUC1 1H
P1 6.25 usec
PL1 -3.00 dB
PL1W 53.12584686 W
SFO1 500.1330885 MHz
SI 32768
SF 500.1300000 MHz
WDW EM
SSB 0
LB 0.30 Hz
GB 0
PC 1.00
12 11 10 9 8 7 6 5 4 3 2 1 ppm
1.96
1.89
0.71
3.97
2.27
1.85
2.48
2.03
4.30
1.93
0.95
0.86

Compound 10

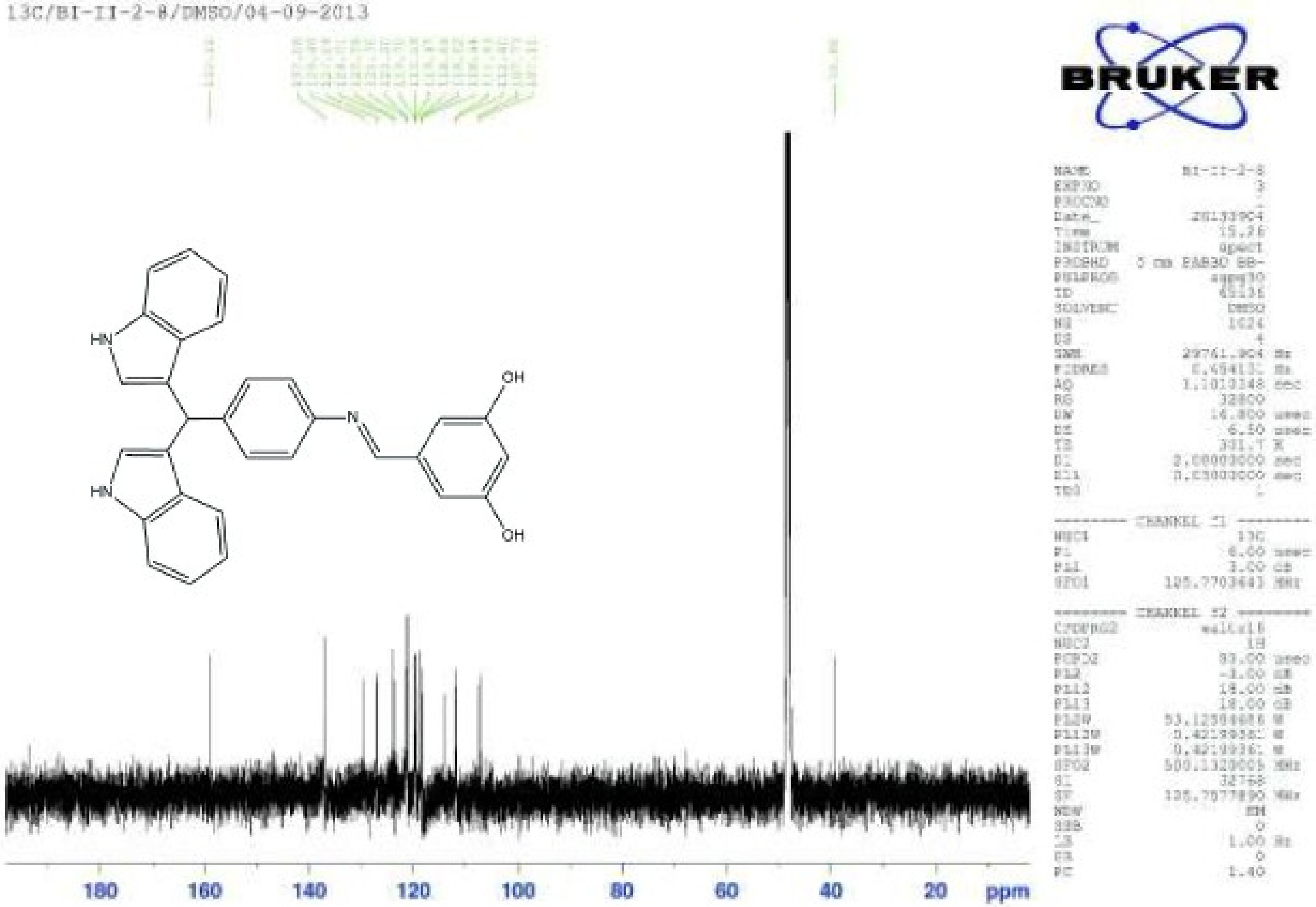
13C/BI-II-2-8/DMSO/04-09-2013
BRUKER
HN
HN
N
OH
OH
180
160
140
120
100
80
60
40
20
ppm

Compound 11

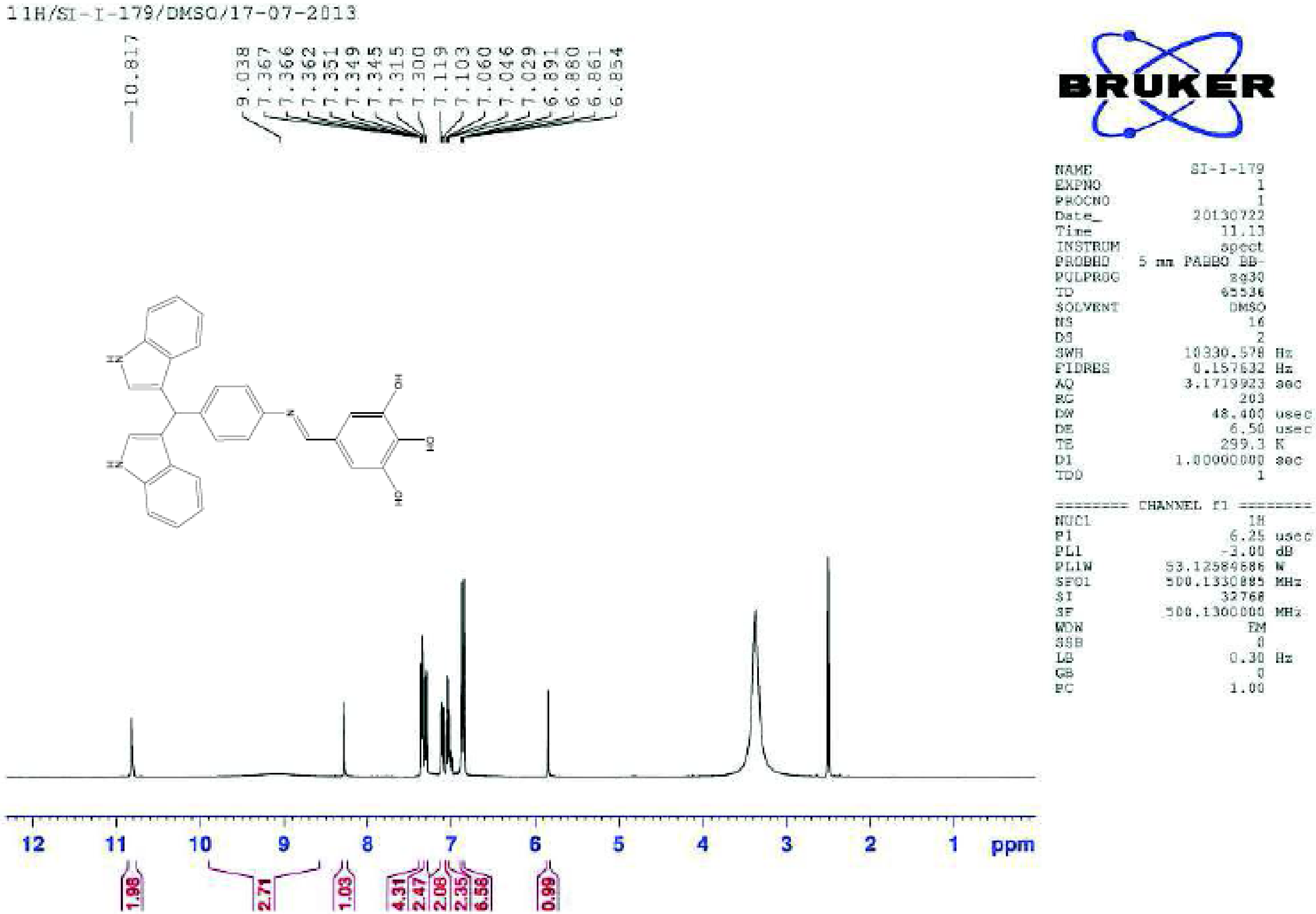
11H/SI-I-179/DMSO/17-07-2013
10.817
9.038
7.367
7.366
7.362
7.351
7.349
7.345
7.315
7.300
7.119
7.103
7.060
7.046
7.029
6.891
6.880
6.861
6.854
BRUKER
NAME SI-I-179
EXPNO 1
PROCNO 1
Date_ 20130722
Time 11.13
INSTRUM spect
PROBHD 5 mm PABBO BB-
PULPROG zg30
TD 65536
SOLVENT DMSO
NS 16
DS 2
SWH 10330.578 Hz
FIDRES 0.157632 Hz
AQ 3.1719923 sec
RG 203
DW 48.400 usec
DE 6.50 usec
TE 299.3 K
D1 1.00000000 sec
TD0 1
======== CHANNEL f1 ========
NUC1 1H
P1 6.25 usec
PL1 -3.00 dB
PL1W 53.12584686 W
SFO1 500.1330885 MHz
SI 32768
SF 500.1300000 MHz
WDW EM
SSB 0
LB 0.30 Hz
GB 0
PC 1.00
12 11 10 9 8 7 6 5 4 3 2 1 ppm
1.98
2.71
1.03
4.31
2.47
2.08
2.35
6.58
0.99

Compound 11

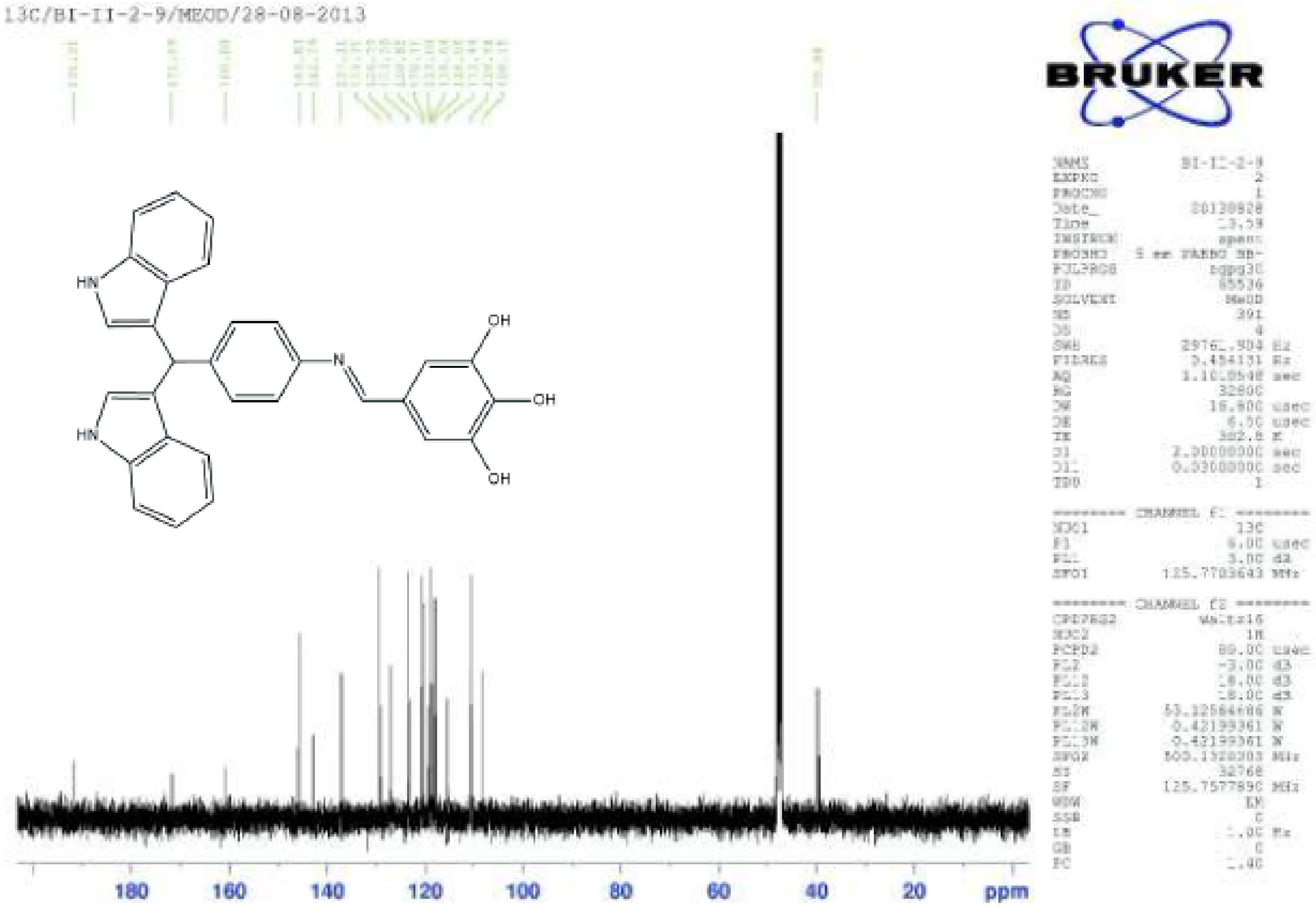

Compound 12

1H/SI-I-181/DMSO/17-07-2013

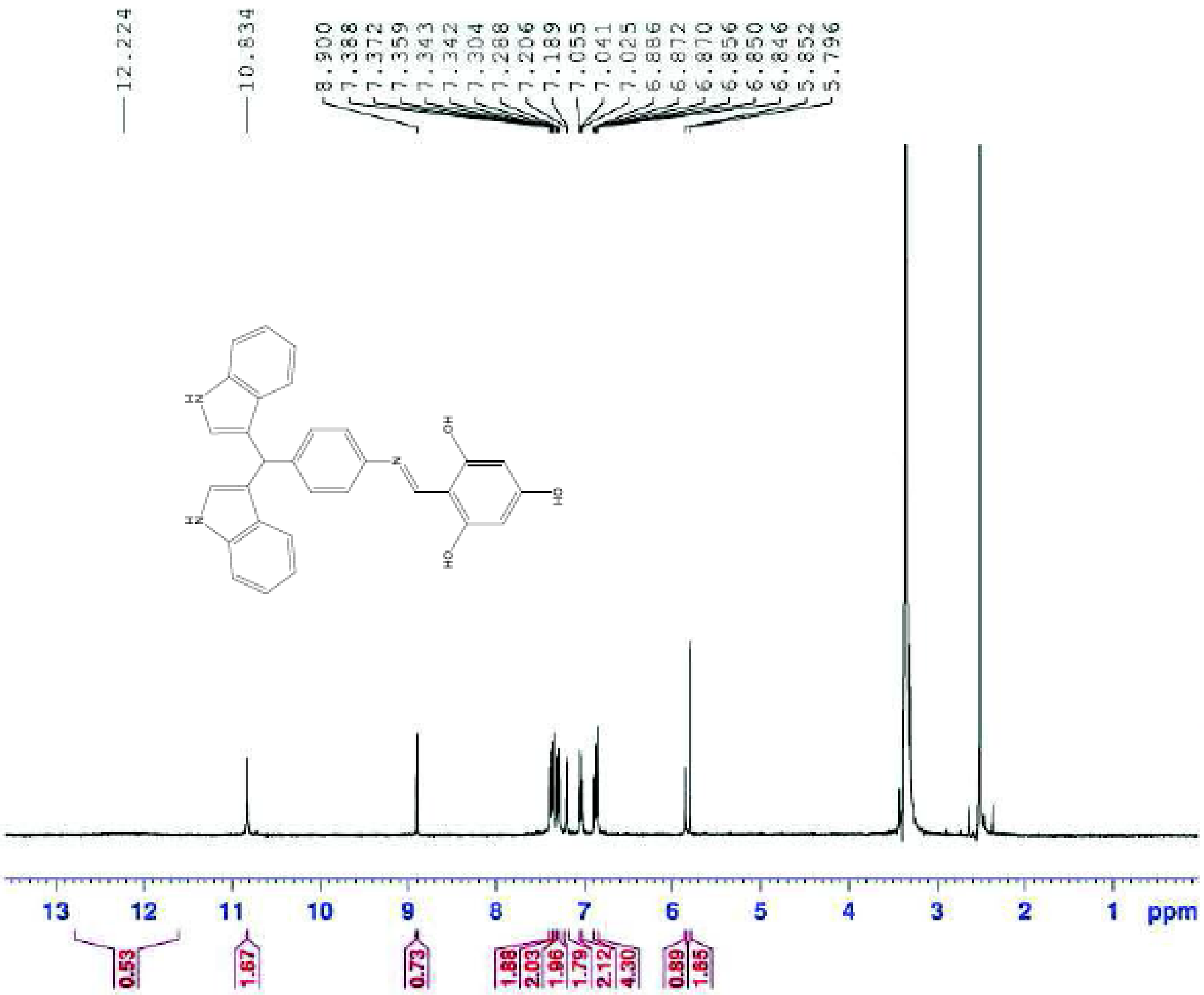

NAME	SI-I-181
EXPNO	1
PROCNO	1
Date_	20130722
Time	11.17
INSTRUM	spect
PROBHD	5 mm PABBO BB-
PULPROG	zg30
TD	65536
SOLVENT	DMSO
NS	16
DS	2
SWH	10330.578 Hz
FIDRES	0.157632 Hz
AQ	3.1719923 sec
RG	406
DW	48.400 usec
DE	6.50 usec
TE	299.2 K
D1	1.00000000 sec
TD0	1

======== CHANNEL f1 ========

NUC1	1H
P1	6.25 usec
PL1	-3.00 dB
PL1W	53.12584686 W
SFO1	500.1330885 MHz
SI	32768
SF	500.1300000 MHz
WDW	EM
SSB	0
LB	0.30 Hz
GB	0
PC	1.00

Compound 12

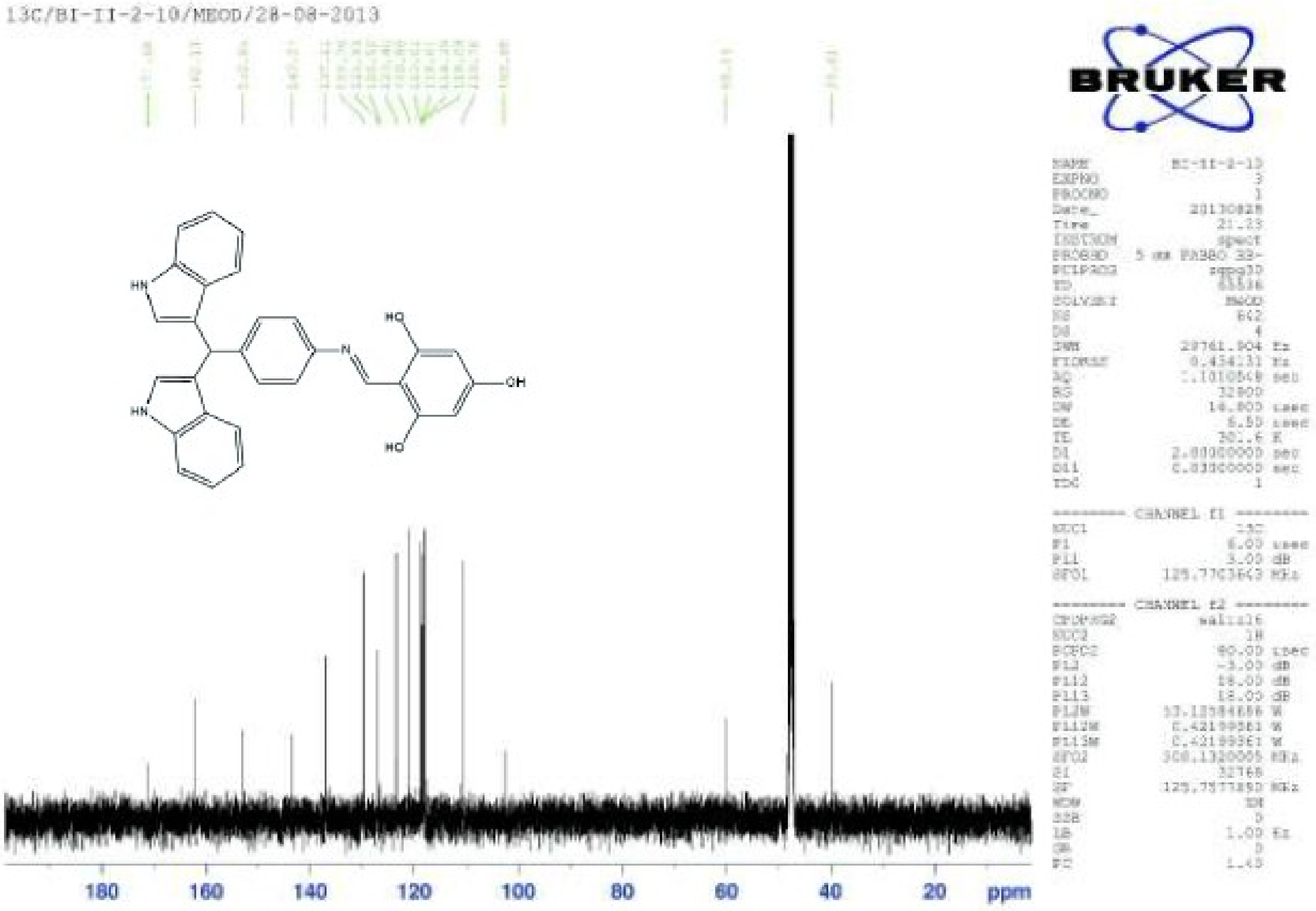
13C/BI-II-2-10/MEOD/28-08-2013
BRUKER
180
160
140
120
100
80
60
40
20
ppm

Compound 13

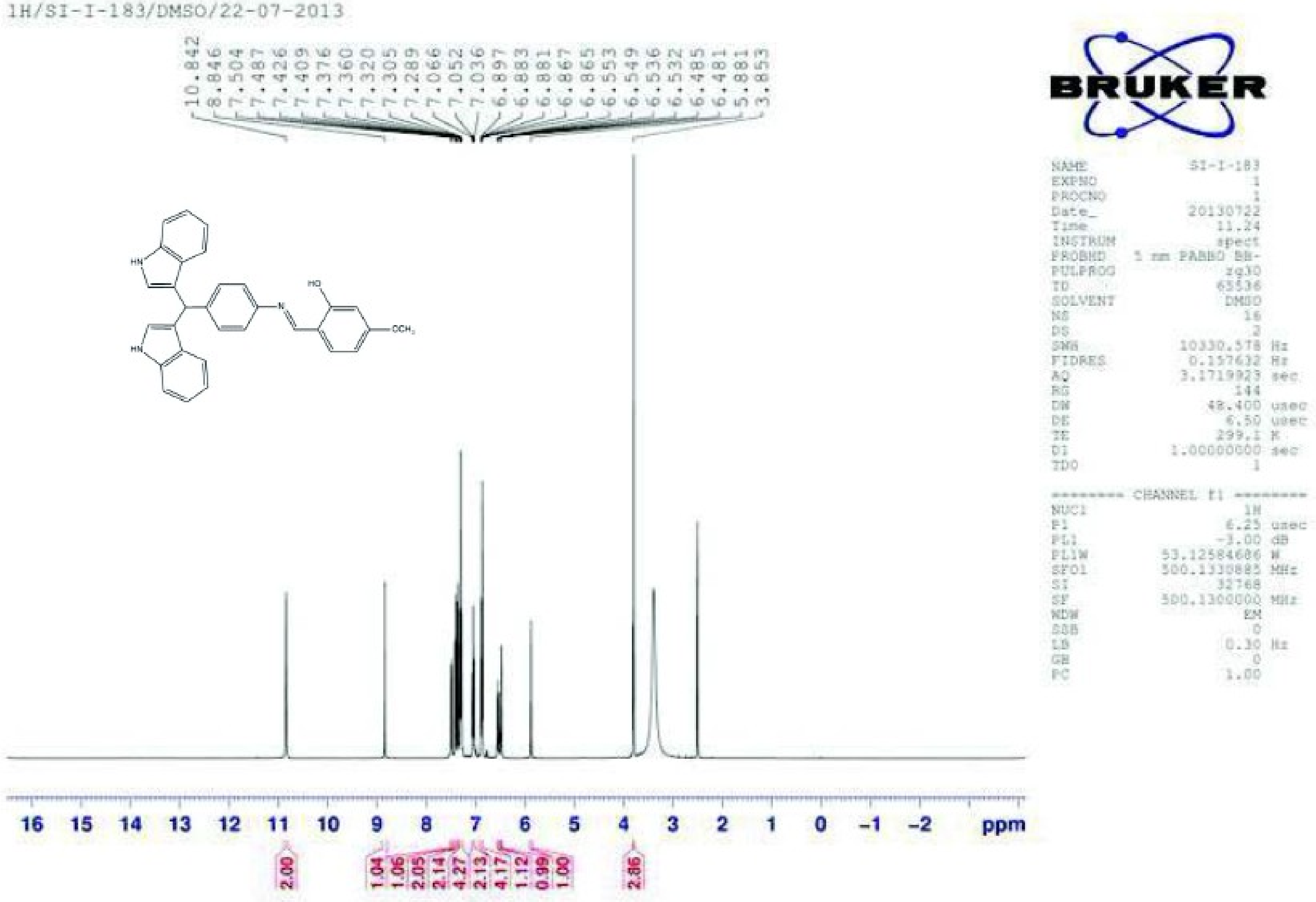
1H/SI-I-183/DMSO/22-07-2013
10.842
8.846
7.504
7.487
7.426
7.409
7.376
7.360
7.320
7.305
7.289
7.066
7.052
7.036
6.897
6.883
6.881
6.867
6.865
6.553
6.549
6.536
6.532
6.485
6.481
5.881
3.853
BRUKER
NAME SI-I-183
EXPNO 1
PROCNO 1
Date_ 20130722
Time 11.24
INSTRUM spect
PROBHD 5 mm PABBO BB-
PULPROG zg30
TD 65536
SOLVENT DMSO
NS 16
DS 2
SWH 10330.578 Hz
FIDRES 0.157632 Hz
AQ 3.1719923 sec
RG 144
DW 48.400 usec
DE 6.50 usec
TE 299.1 K
D1 1.00000000 sec
TD0 1
======== CHANNEL f1 ========
NUC1 1H
P1 6.25 usec
PL1 -3.00 dB
PL1W 53.12584686 W
SFO1 500.1330885 MHz
SI 32768
SF 500.1300000 MHz
WDW EM
SSB 0
LB 0.30 Hz
GB 0
PC 1.00
ppm
2.00
1.04
1.06
2.05
2.14
4.27
2.13
4.17
1.12
0.99
1.00
2.86

Compound 13

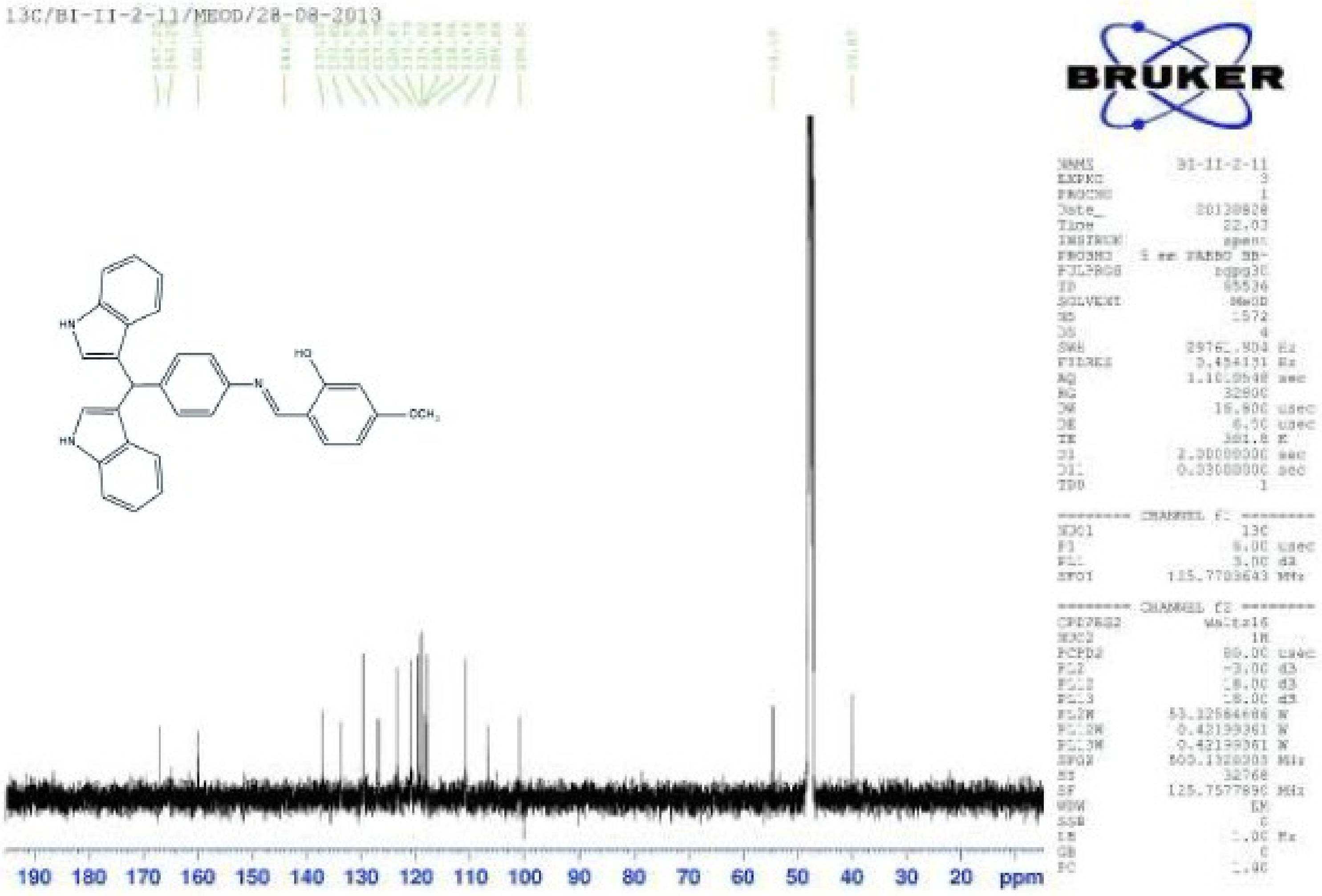
13C/BI-II-2-11/MEOD/28-08-2013
HN
HN
HO
N
OCH3
190 180 170 160 150 140 130 120 110 100 90 80 70 60 50 40 30 20 ppm

Compound 14

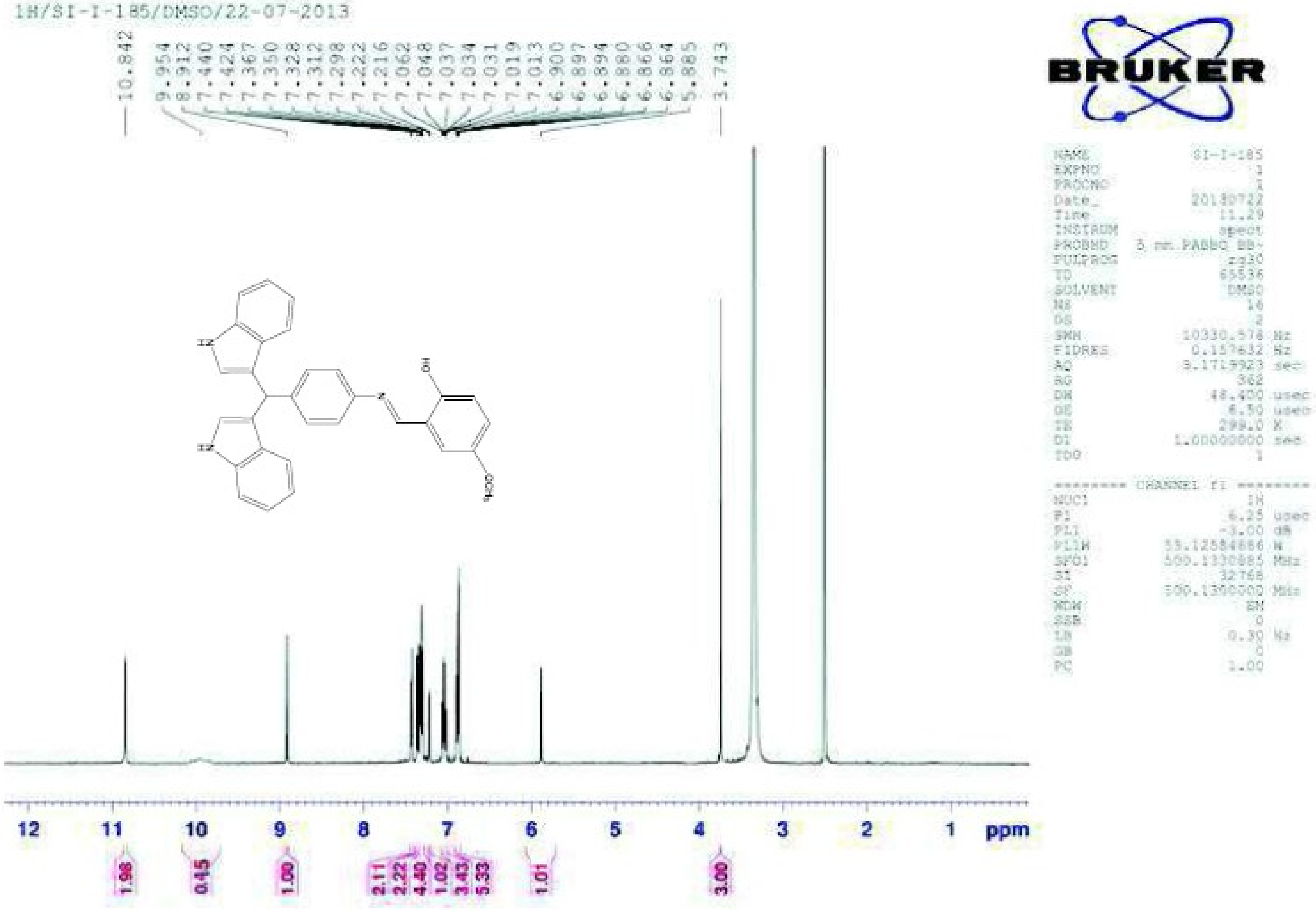
1H/SI-I-185/DMSO/22-07-2013
BRUKER
NAME SI-I-185
EXPNO 1
PROCNO 1
SOLVENT DMSO
NS 16
DS 2
NUC1 1H
ppm

Compound 14

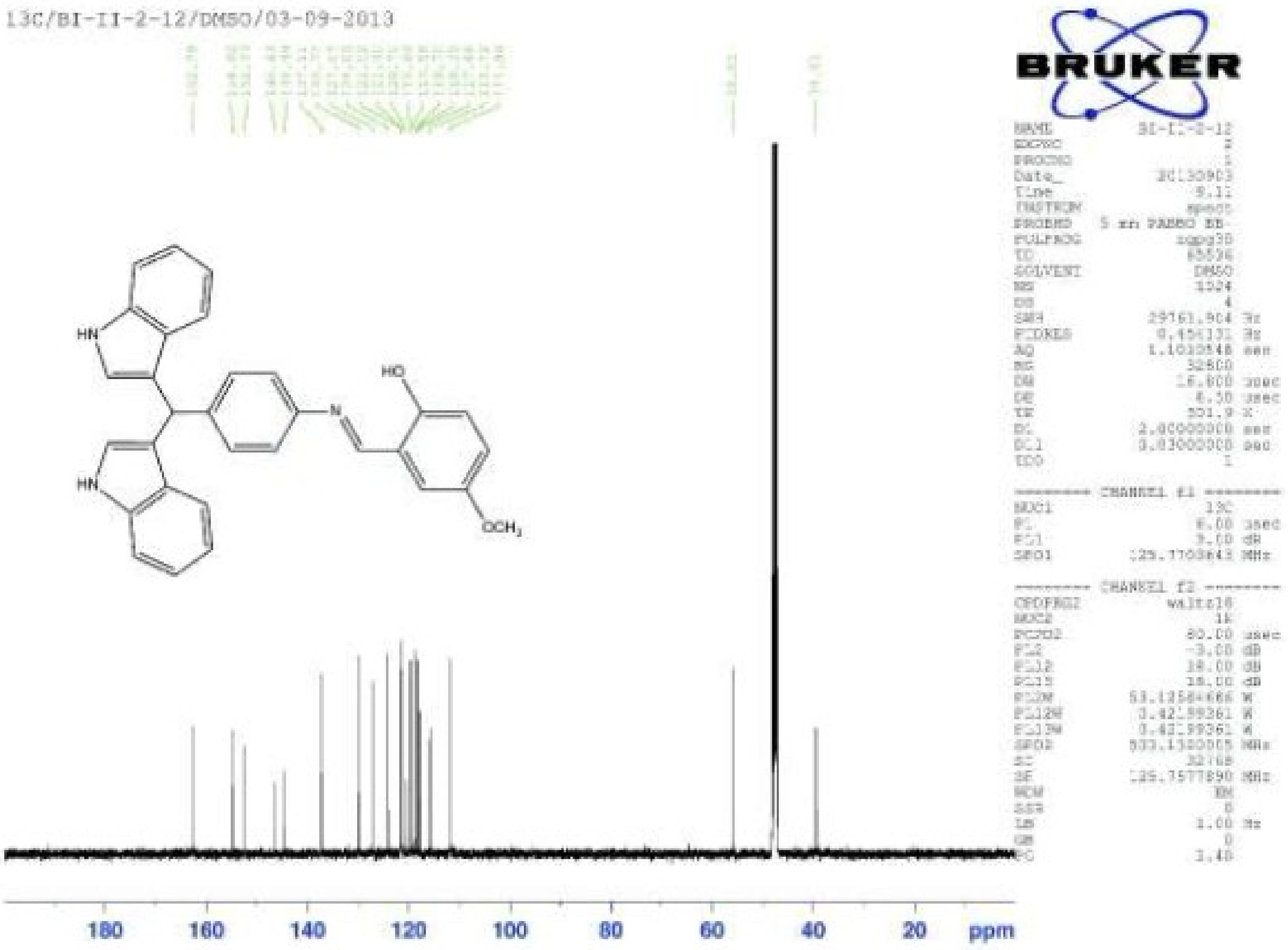
13C/BI-II-2-12/DMSO/03-09-2013
HN
HN
HO
N
OCH3
180
160
140
120
100
80
60
40
20
ppm

Compound 15

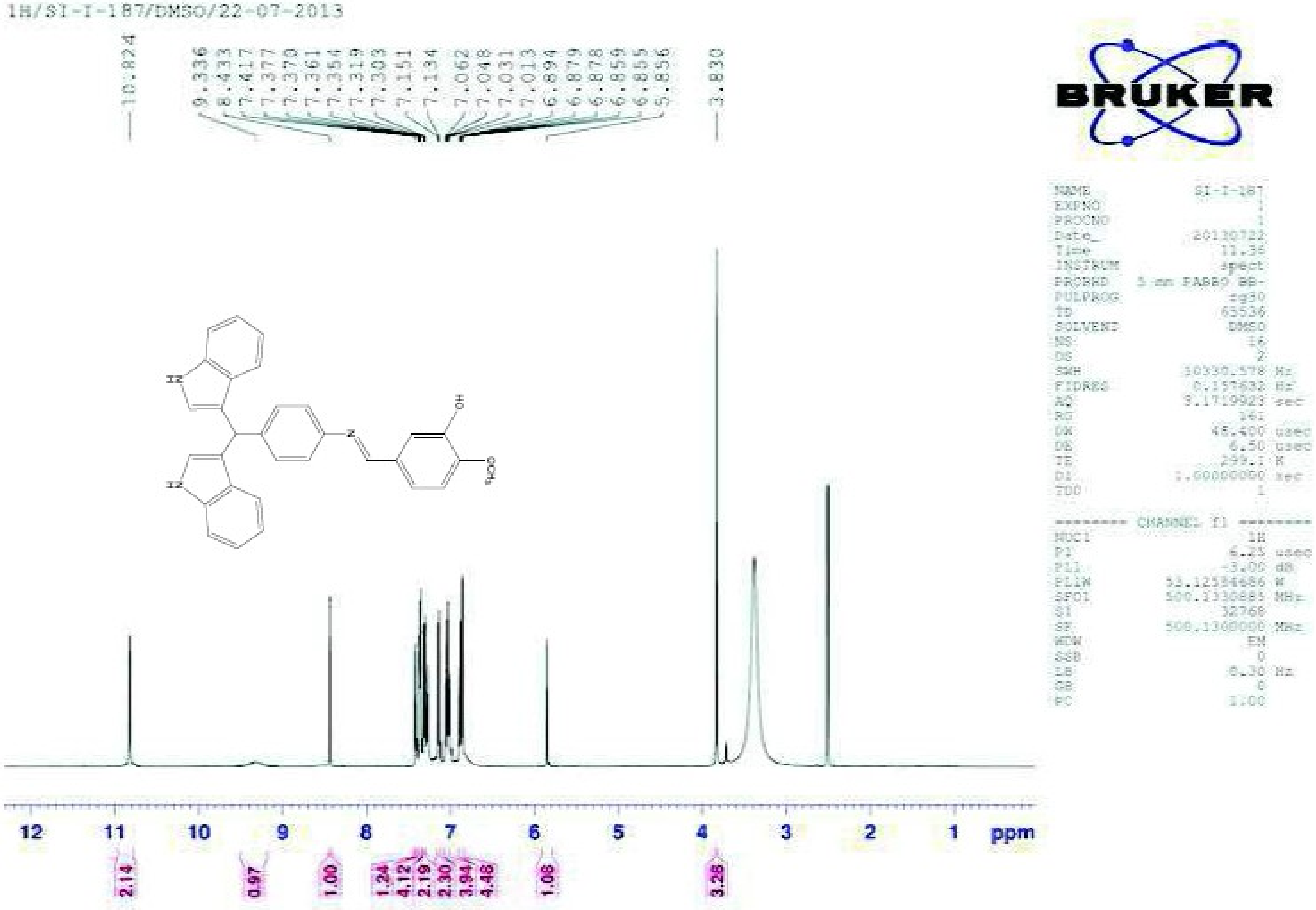
1H/SI-I-187/DMSO/22-07-2013
10.824
9.336
8.433
7.417
7.377
7.370
7.361
7.354
7.319
7.303
7.151
7.134
7.062
7.048
7.031
7.013
6.894
6.879
6.878
6.859
6.855
5.856
3.830
BRUKER
NAME SI-I-187
EXPNO 1
PROCNO 1
Date_ 20130722
Time 11.36
INSTRUM spect
SOLVENT DMSO
NS 16
DS 2
SWH 10330.578 Hz
RG 161
TE 299.1 K
D1 1.00000000 sec
TD0 1
CHANNEL f1
NUC1 1H
SFO1 500.1330885 MHz
SI 32768
SF 500.1300000 MHz
WDW EM
LB 0.30 Hz
PC 1.00
OH
OCH3
12
11
10
9
8
7
6
5
4
3
2
1
ppm
2.14
0.97
1.00
1.24
4.12
2.19
2.30
3.94
4.48
1.08
3.28

Compound 15

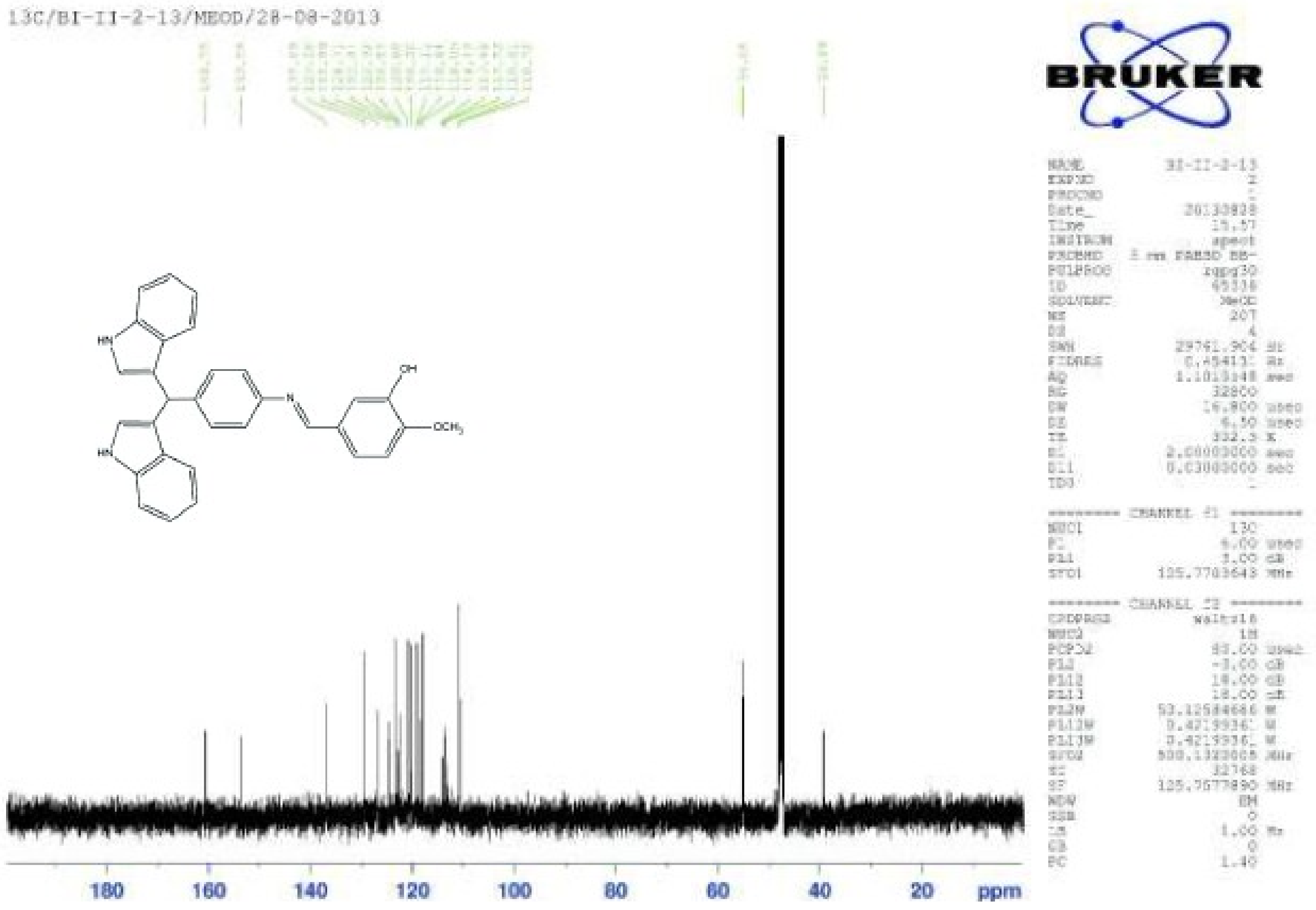
13C/BI-II-2-13/MEOD/28-08-2013
BRUKER
HN
HN
OH
N
OCH3
180
160
140
120
100
80
60
40
20
ppm

Compound 16

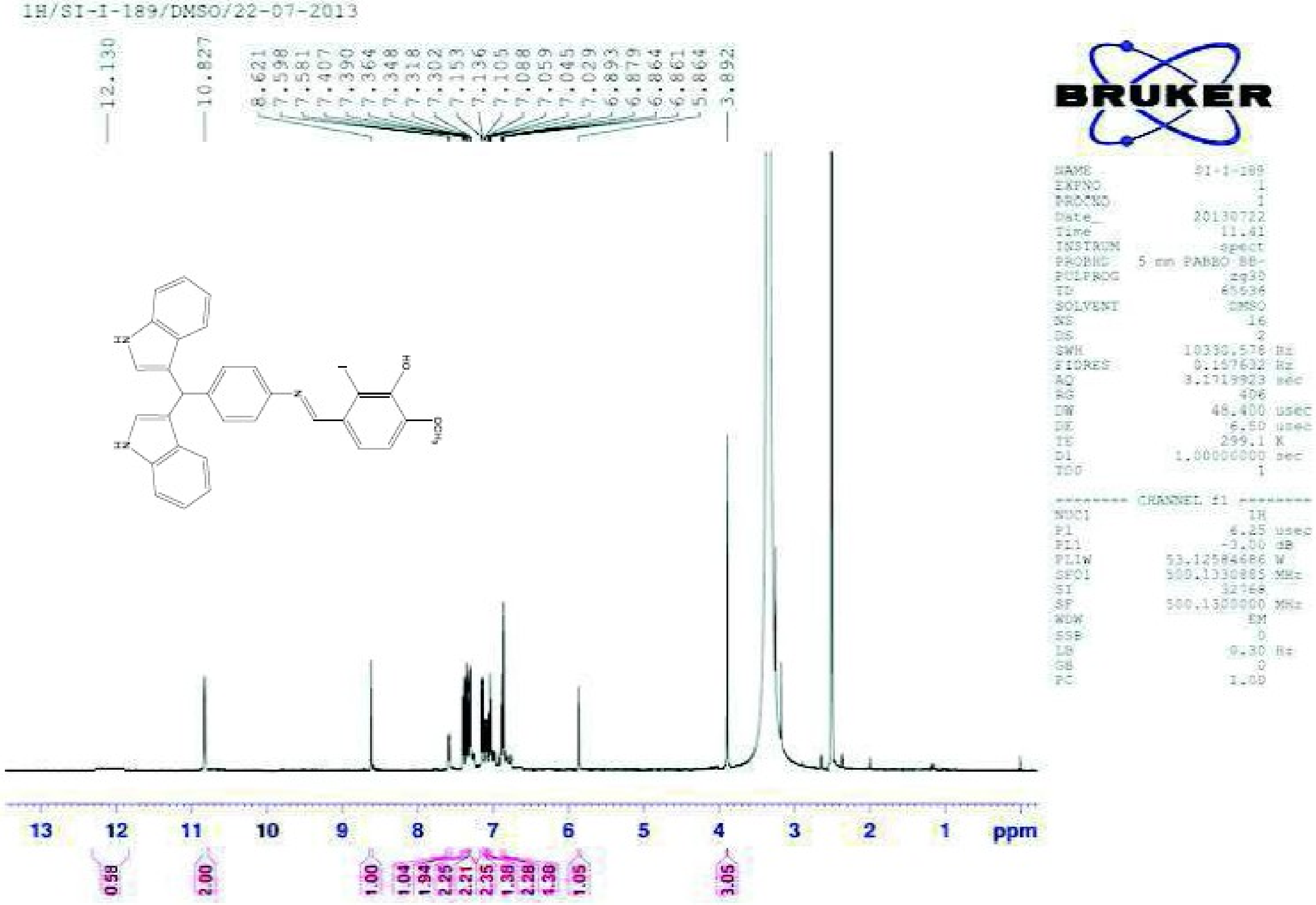

Compound 16

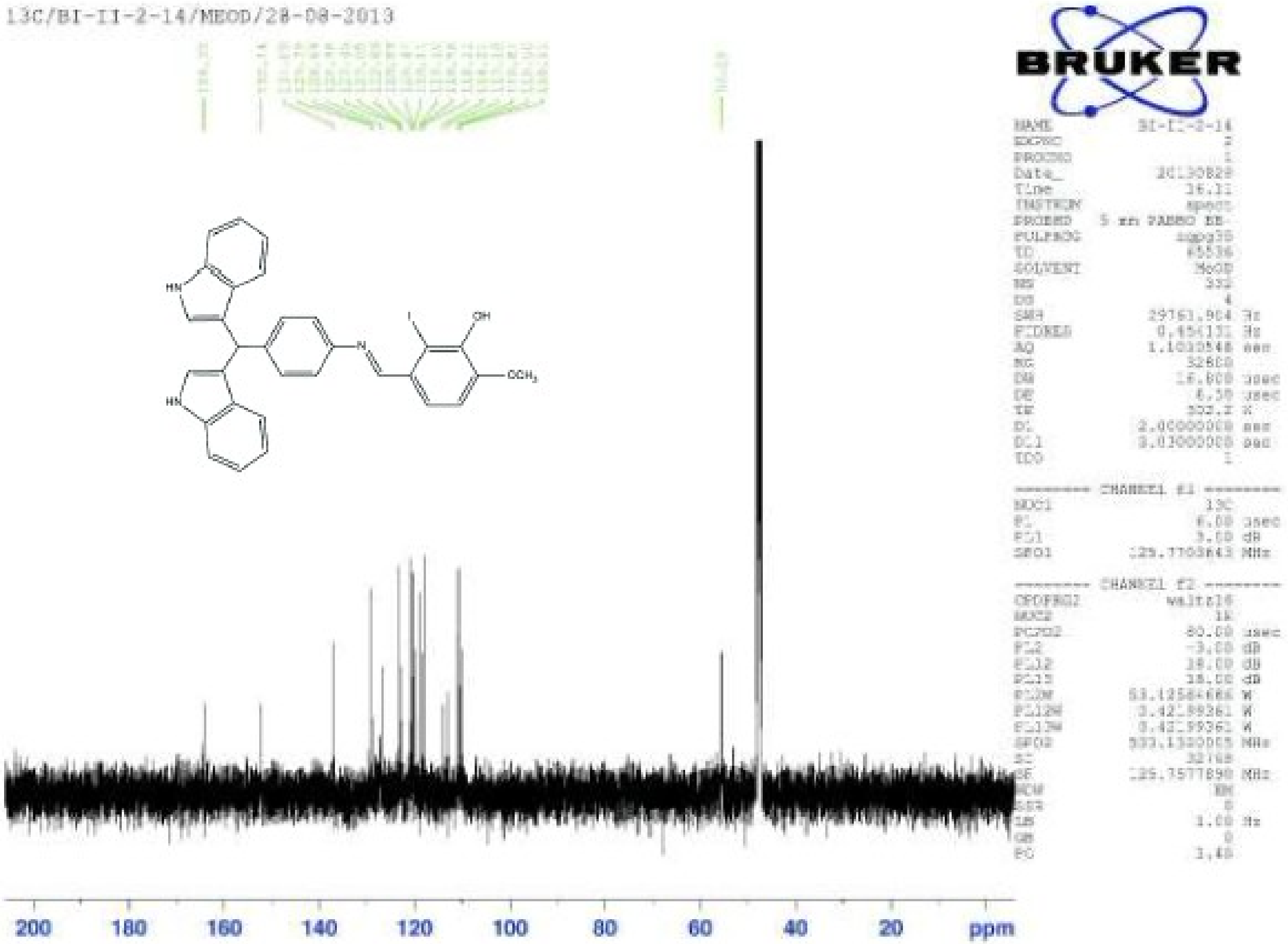

Compound 17

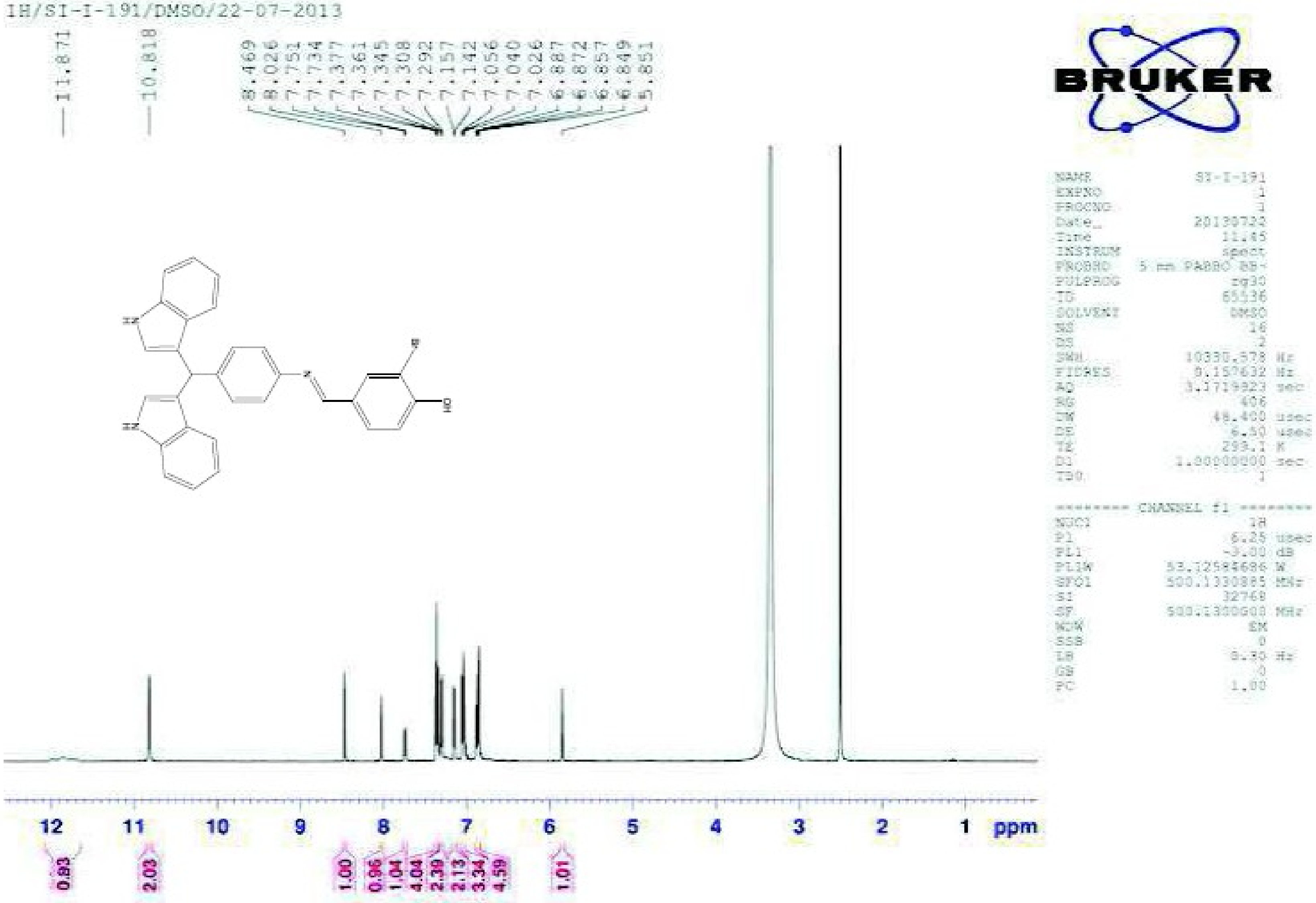
1H/SI-I-191/DMSO/22-07-2013
11.871
10.818
8.469 8.026 7.751 7.734 7.377 7.361 7.345 7.308 7.292 7.157 7.142 7.056 7.040 7.026 6.887 6.872 6.857 6.849 5.851
BRUKER
NAME SI-I-191
EXPNO 1
PROCNO 1
Date_ 20130722
Time 11.45
INSTRUM spect
PROBHD 5 mm PABBO BB-
PULPROG zg30
TD 65536
SOLVENT DMSO
NS 16
DS 2
SWH 10330.578 Hz
FIDRES 0.157632 Hz
AQ 3.1719923 sec
RG 406
DW 48.400 usec
DE 6.50 usec
TE 299.1 K
D1 1.00000000 sec
TD0 1
======== CHANNEL f1 ========
NUC1 1H
P1 6.25 usec
PL1 -3.00 dB
PL1W 53.12584686 W
SFO1 500.1330885 MHz
SI 32768
SF 500.1300000 MHz
WDW EM
SSB 0
LB 0.30 Hz
GB 0
PC 1.00
12 11 10 9 8 7 6 5 4 3 2 1 ppm
0.93 2.03 1.00 0.96 1.04 4.04 2.30 2.13 3.34 4.59 1.01

Compound 17

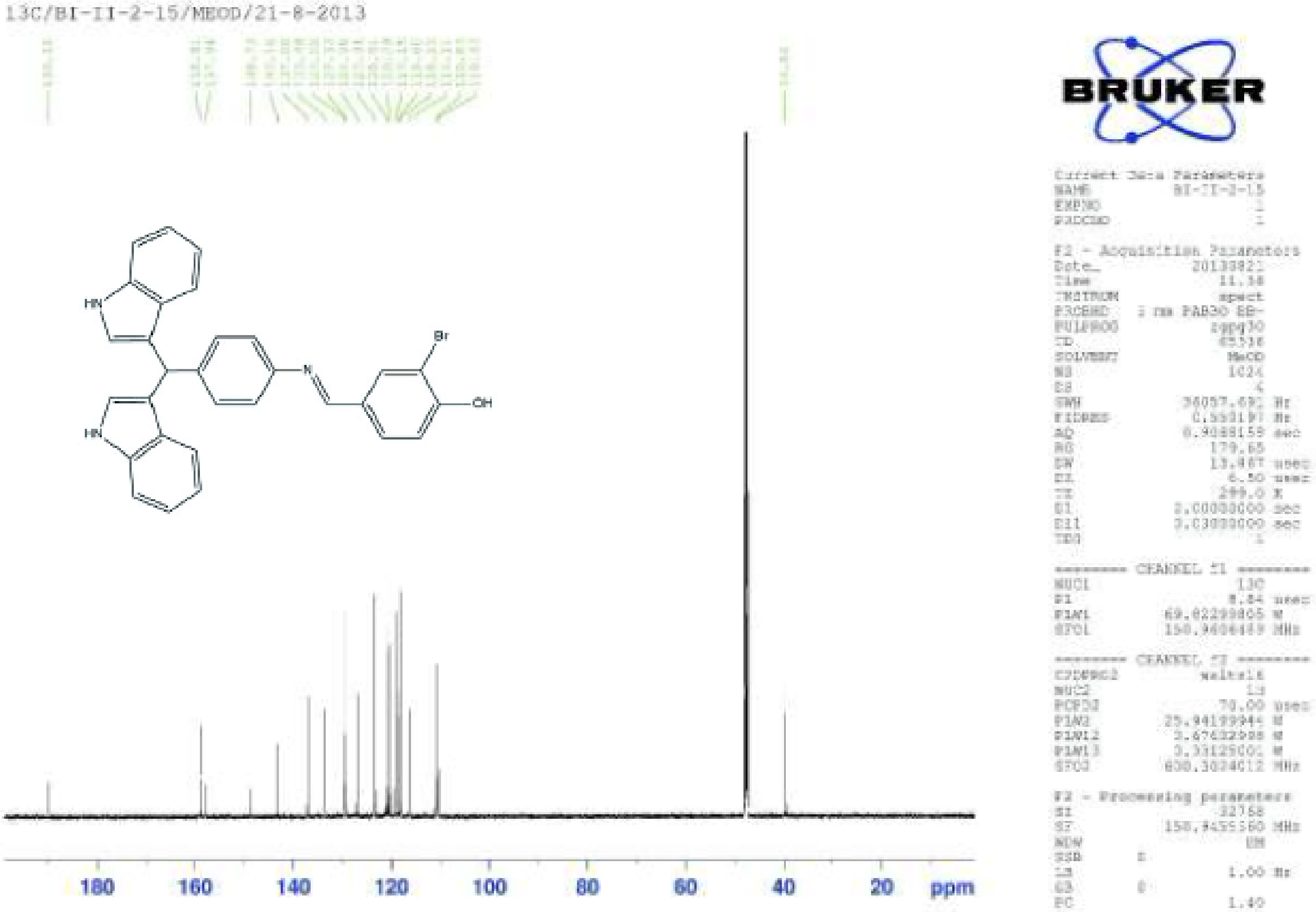
13C/BI-II-2-15/MEOD/21-8-2013
HN
HN
N
Br
OH
BRUKER
180
160
140
120
100
80
60
40
20
ppm

Compound 18

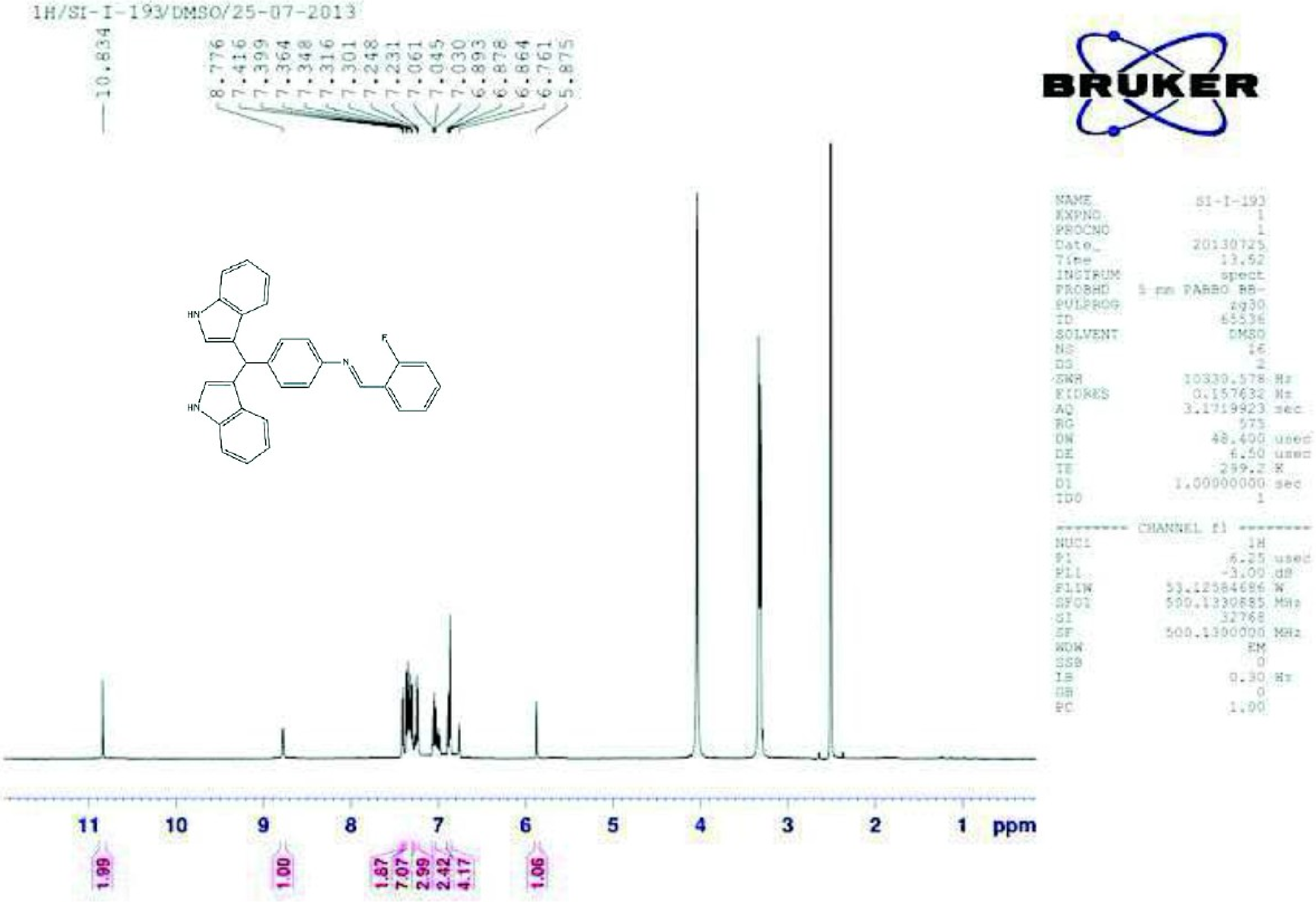
1H/SI-I-193/DMSO/25-07-2013
10.834
8.776 7.416 7.399 7.364 7.348 7.316 7.301 7.248 7.231 7.061 7.045 7.030 6.893 6.878 6.864 6.761 5.875
HN
HN
F
N
BRUKER
NAME SI-I-193
EXPNO 1
PROCNO 1
Date_ 20130725
Time 13.52
INSTRUM spect
PROBHD 5 mm PABBO BB-
PULPROG zg30
TD 65536
SOLVENT DMSO
NS 16
DS 2
SWH 10330.578 Hz
FIDRES 0.157632 Hz
AQ 3.1719923 sec
RG 575
DW 48.400 usec
DE 6.50 usec
TE 299.2 K
D1 1.00000000 sec
TD0 1
======== CHANNEL f1 ========
NUC1 1H
P1 6.25 usec
PL1 -3.00 dB
PL1W 53.12584686 W
SFO1 500.1330885 MHz
SI 32768
SF 500.1300000 MHz
WDW EM
SSB 0
LB 0.30 Hz
GB 0
PC 1.00
11 10 9 8 7 6 5 4 3 2 1 ppm
1.99 1.00 1.87 7.07 2.99 2.42 4.17 1.06

Compound 18

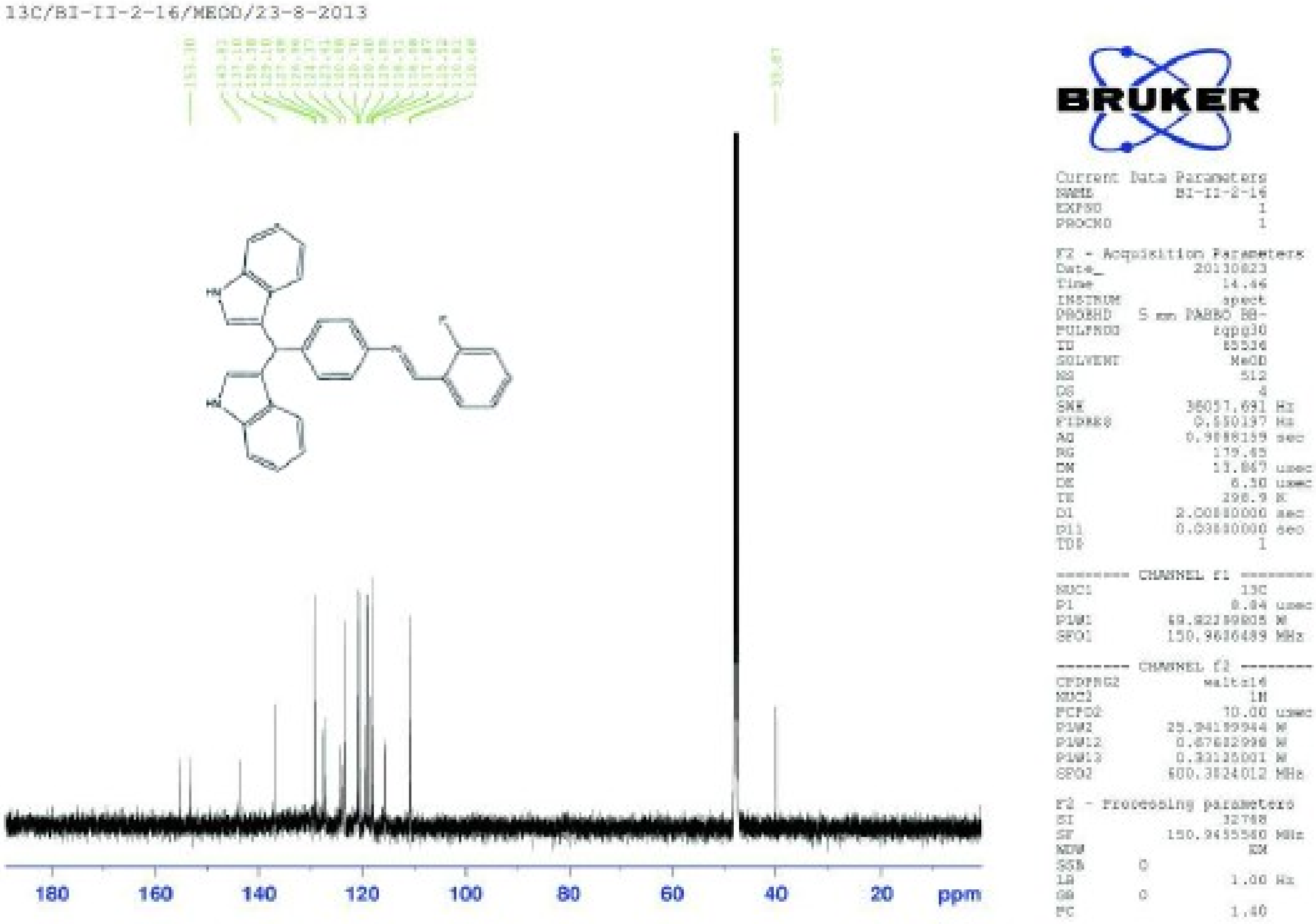

13C/BI-II-2-16/MEOD/23-8-2013
BRUKER
Current Data Parameters
NAME BI-II-2-16
EXPNO 1
PROCNO 1
F2 - Acquisition Parameters
Date_ 20130823
Time 14.46
INSTRUM spect
PROBHD 5 mm PABBO BB-
PULPROG zgpg30
TD 65536
SOLVENT MeOD
NS 512
DS 4
SWH 36057.691 Hz
FIDRES 0.550197 Hz
AQ 0.9088159 sec
RG 179.65
DW 13.867 usec
DE 6.50 usec
TE 298.9 K
D1 2.00000000 sec
D11 0.03000000 sec
TD0 1
======== CHANNEL f1 ========
NUC1 13C
P1 8.84 usec
SFO1 150.9636489 MHz
======== CHANNEL f2 ========
CPDPRG2 waltz16
NUC2 1H
PCPD2 70.00 usec
SFO2 600.3824012 MHz
F2 - Processing parameters
SI 32768
SF 150.9455560 MHz
WDW EM
SSB 0
LB 1.00 Hz
GB 0
PC 1.40
180
160
140
120
100
80
60
40
20
ppm

Compound 19

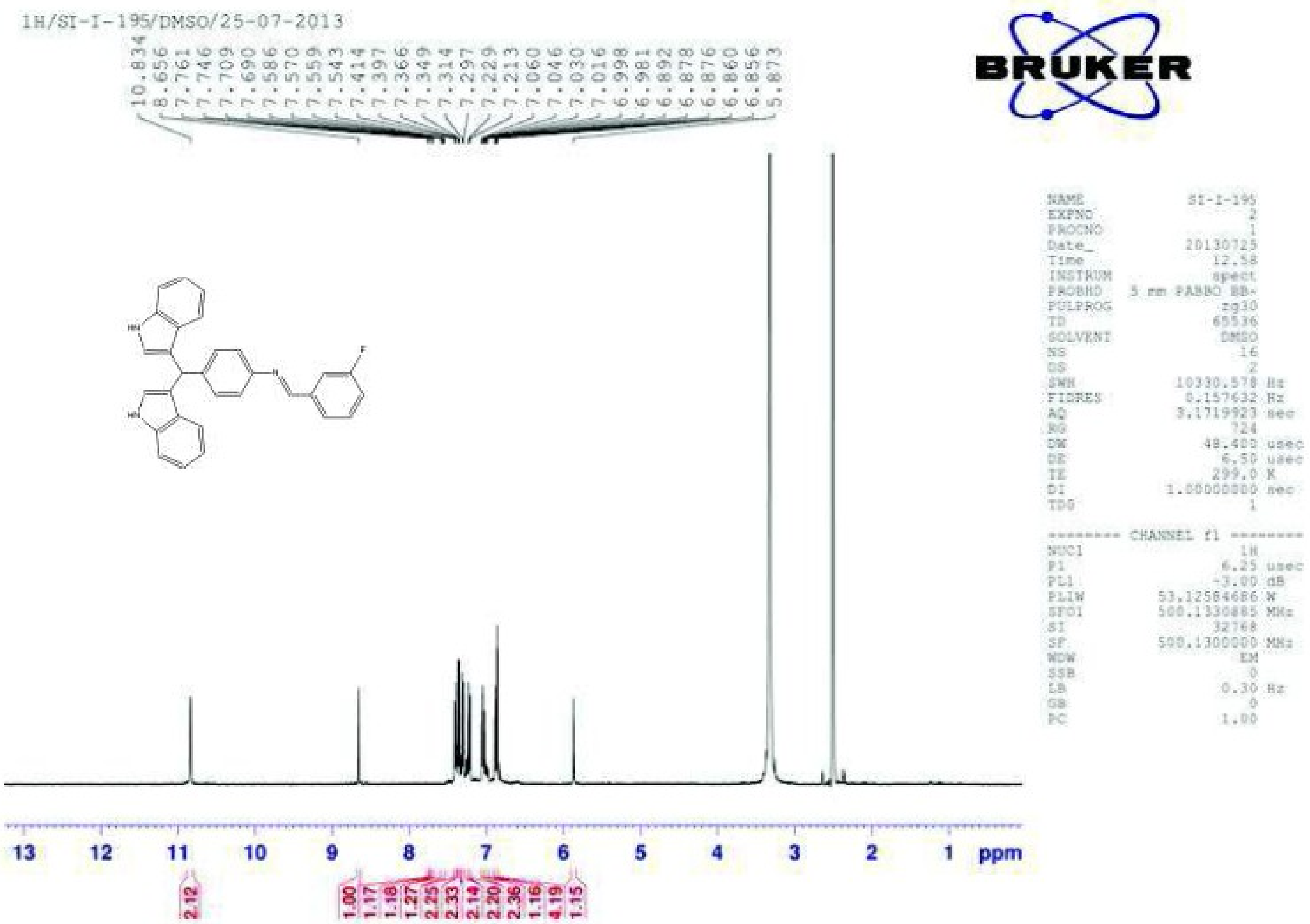
1H/SI-I-195/DMSO/25-07-2013
10.834
8.656
7.761
7.746
7.709
7.690
7.586
7.570
7.559
7.543
7.414
7.397
7.366
7.349
7.314
7.297
7.229
7.213
7.060
7.046
7.030
7.016
6.998
6.981
6.892
6.878
6.876
6.860
6.856
5.873
NAME SI-I-195
EXPNO 2
PROCNO 1
Date_ 20130725
Time 12.58
INSTRUM spect
PROBHD 5 mm PABBO BB-
PULPROG zg30
TD 65536
SOLVENT DMSO
NS 16
DS 2
SWH 10330.578 Hz
FIDRES 0.157632 Hz
AQ 3.1719923 sec
RG 724
DW 48.400 usec
DE 6.50 usec
TE 299.0 K
D1 1.00000000 sec
TD0 1
======== CHANNEL f1 ========
NUC1 1H
P1 6.25 usec
PL1 -3.00 dB
PL1W 53.12564686 W
SFO1 500.1330885 MHz
SI 32768
SF 500.1300000 MHz
WDW EM
SSB 0
LB 0.30 Hz
GB 0
PC 1.00
13 12 11 10 9 8 7 6 5 4 3 2 1 ppm
2.12
1.00
1.17
1.18
1.27
2.25
2.33
2.14
2.20
2.36
1.16
4.19
1.15

Compound 19

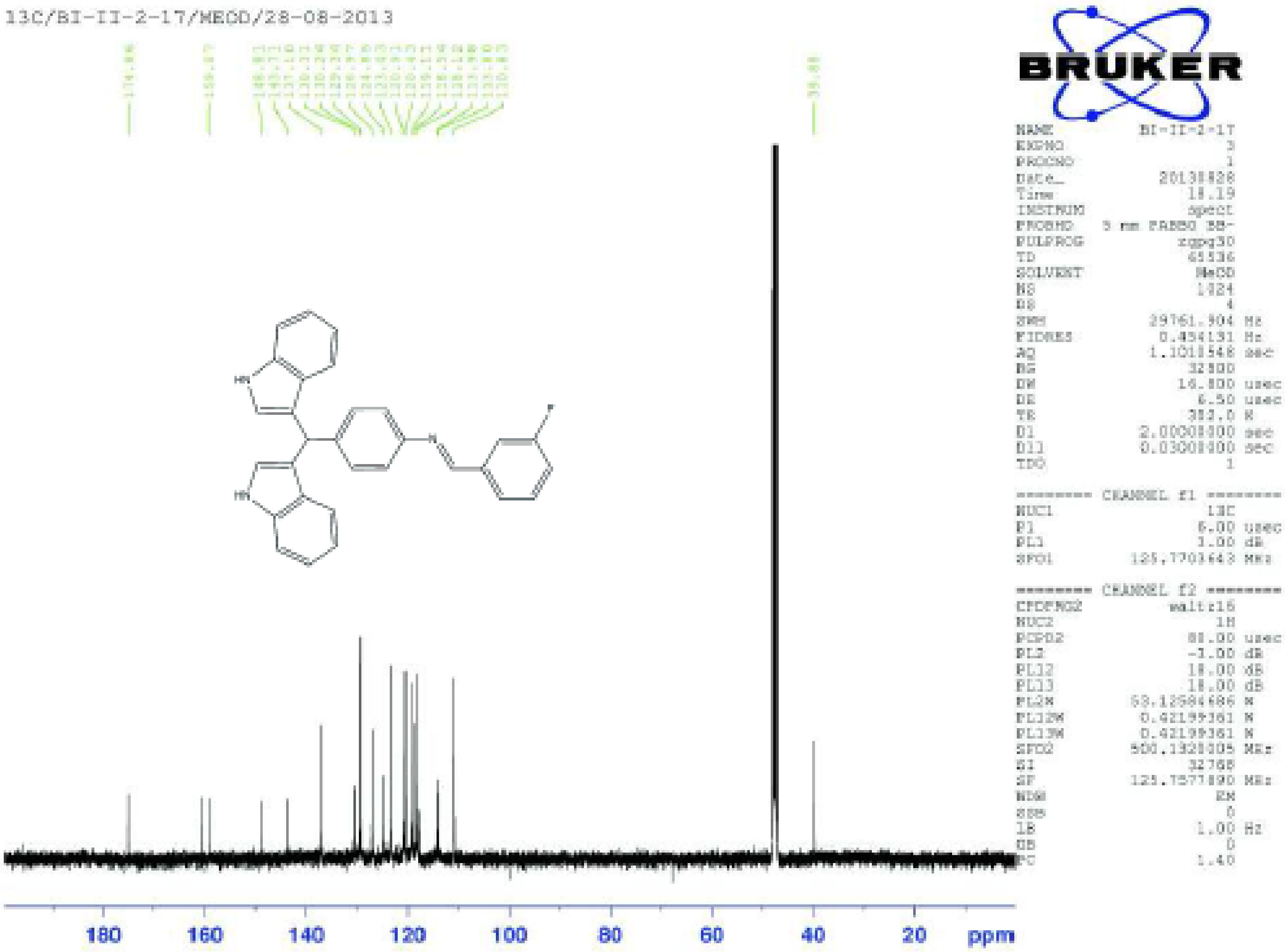
13C/BI-II-2-17/MEOD/28-08-2013
BRUKER
ppm
180
160
140
120
100
80
60
40
20

Compound 20

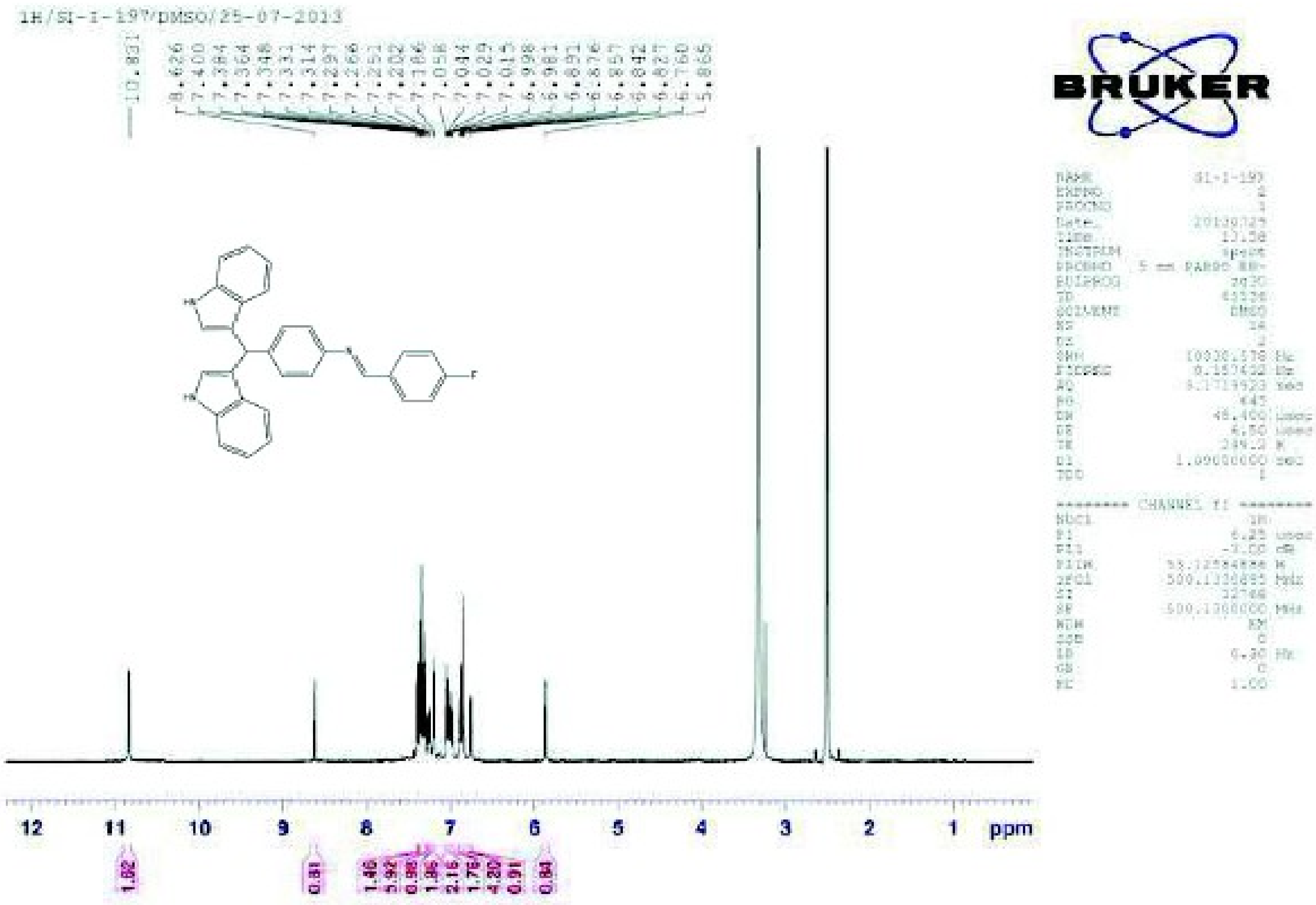

Compound 20

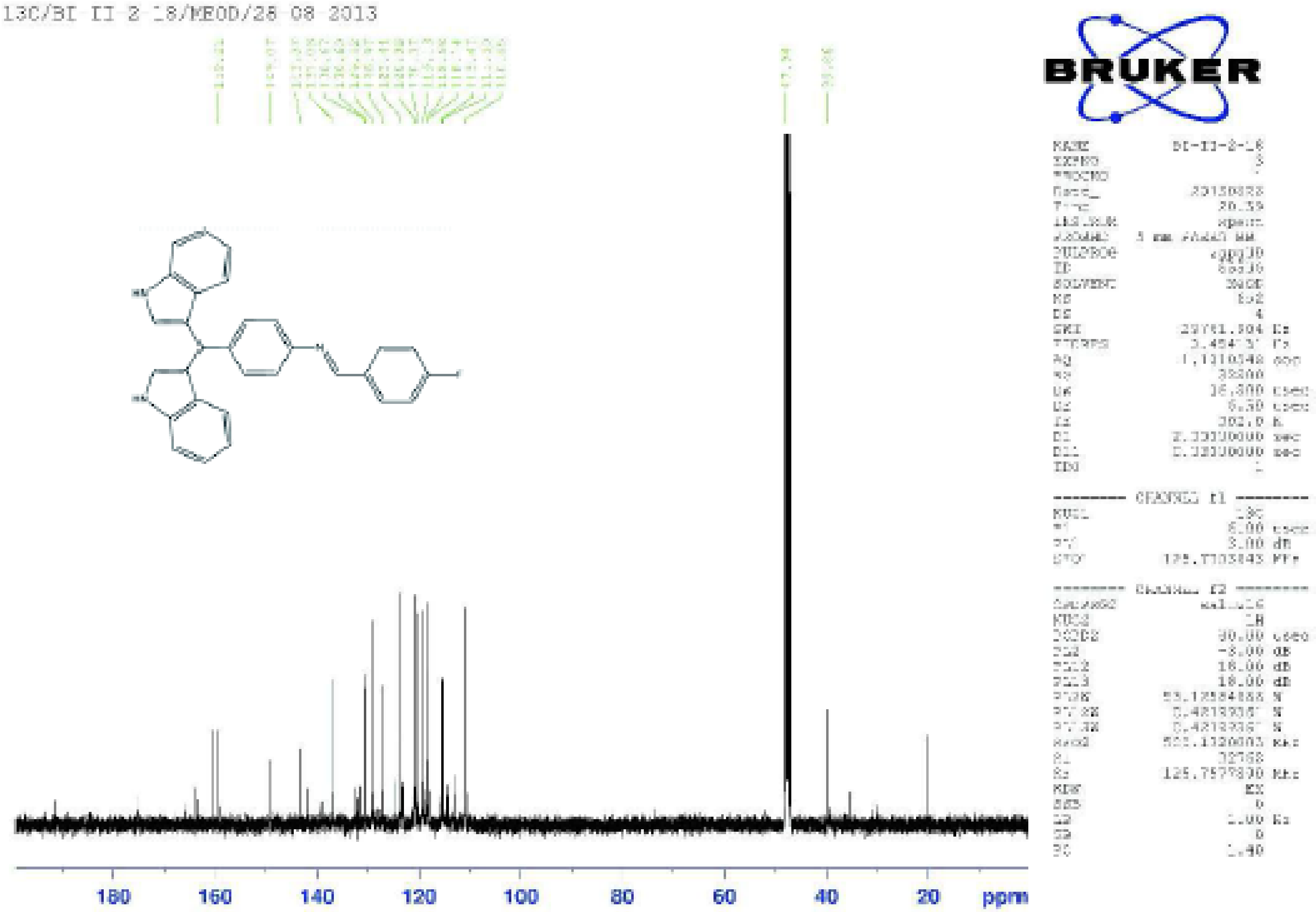

Compound 21

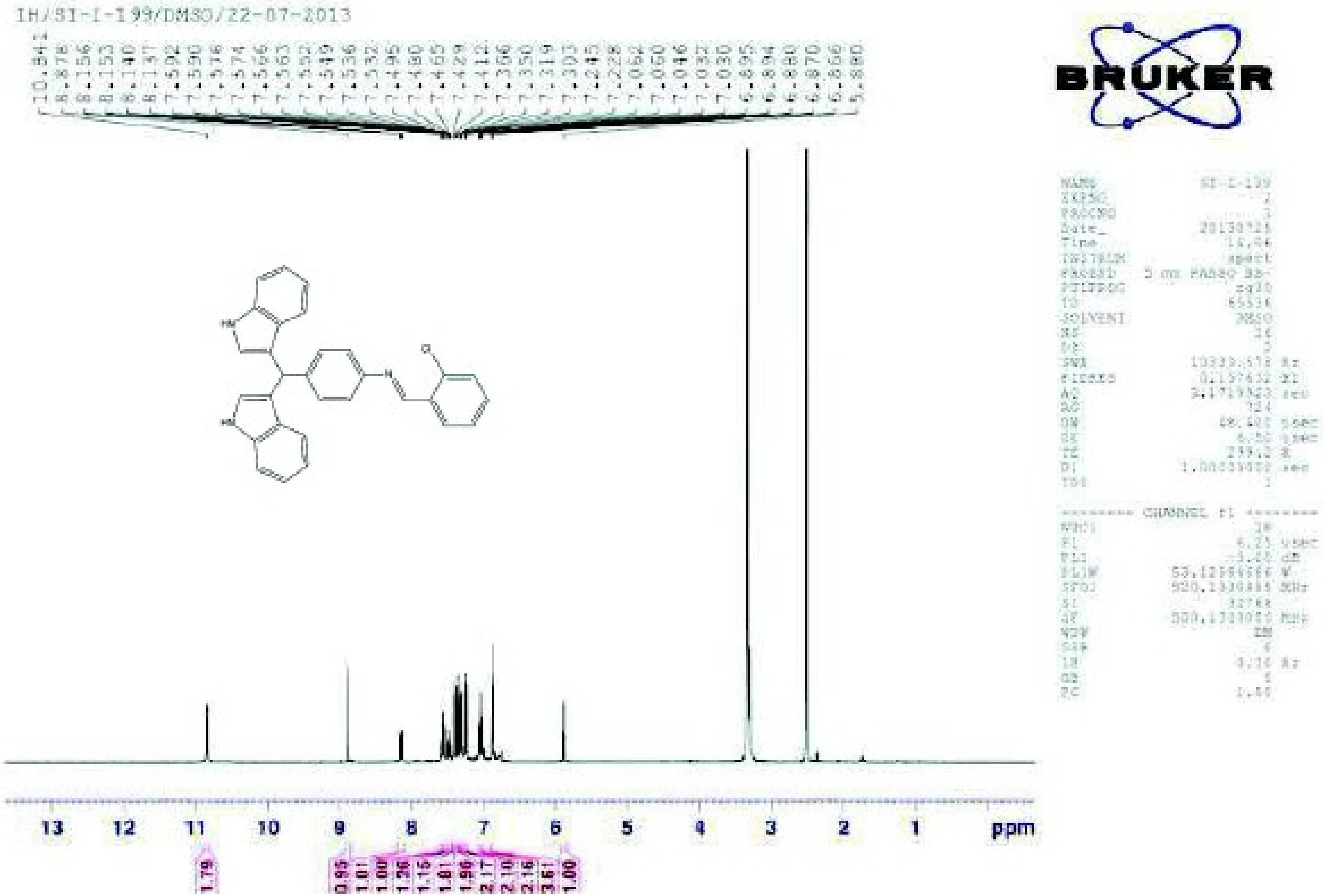

Compound 21

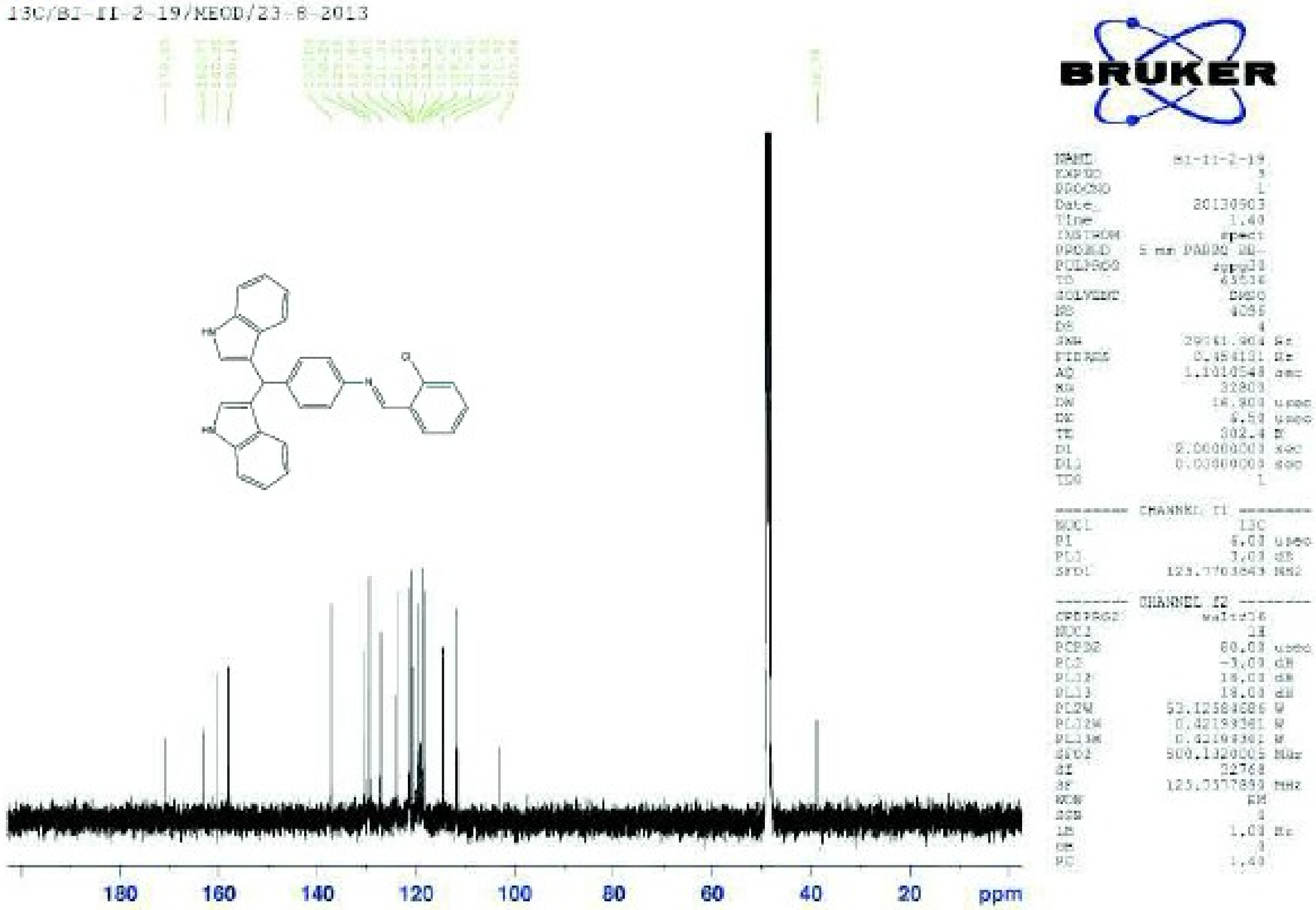
13C/BI-II-2-19/MEOD/23-8-2013
BRUKER
NAME
EXPNO
PROCNO
Date_
Time
INSTRUM
PROBHD
PULPROG
TD
SOLVENT
NS
DS
SWH
FIDRES
AQ
RG
DW
DE
TE
D1
D11
TD0
CHANNEL f1
NUC1
P1
PL1
SFO1
CHANNEL f2
CPDPRG2
NUC2
PCPD2
PL2
PL12
PL13
PL2W
PL12W
PL13W
SFO2
SI
SF
WDW
SSB
LB
GB
PC
180
160
140
120
100
80
60
40
20
ppm

Compound 22

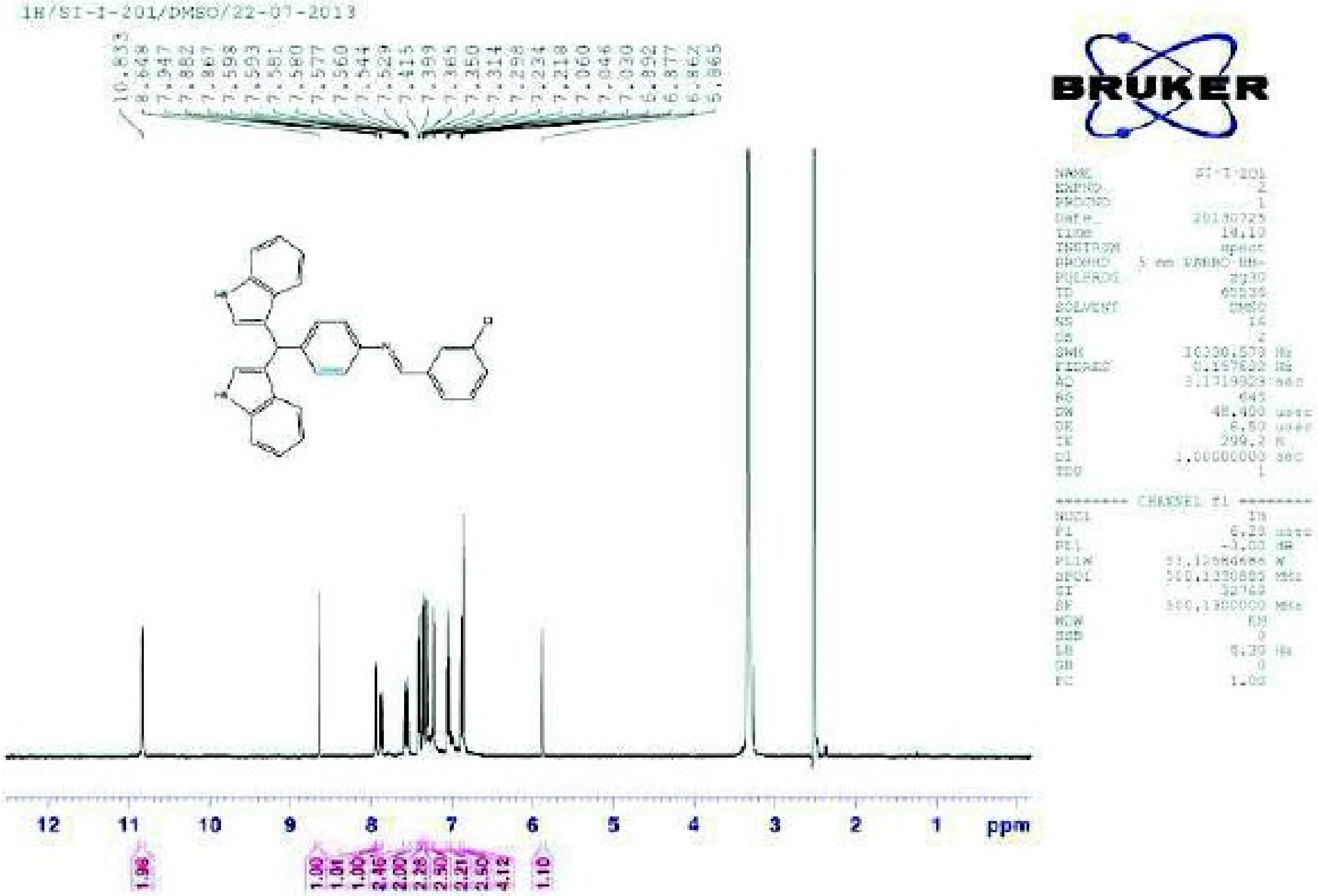

Compound 22

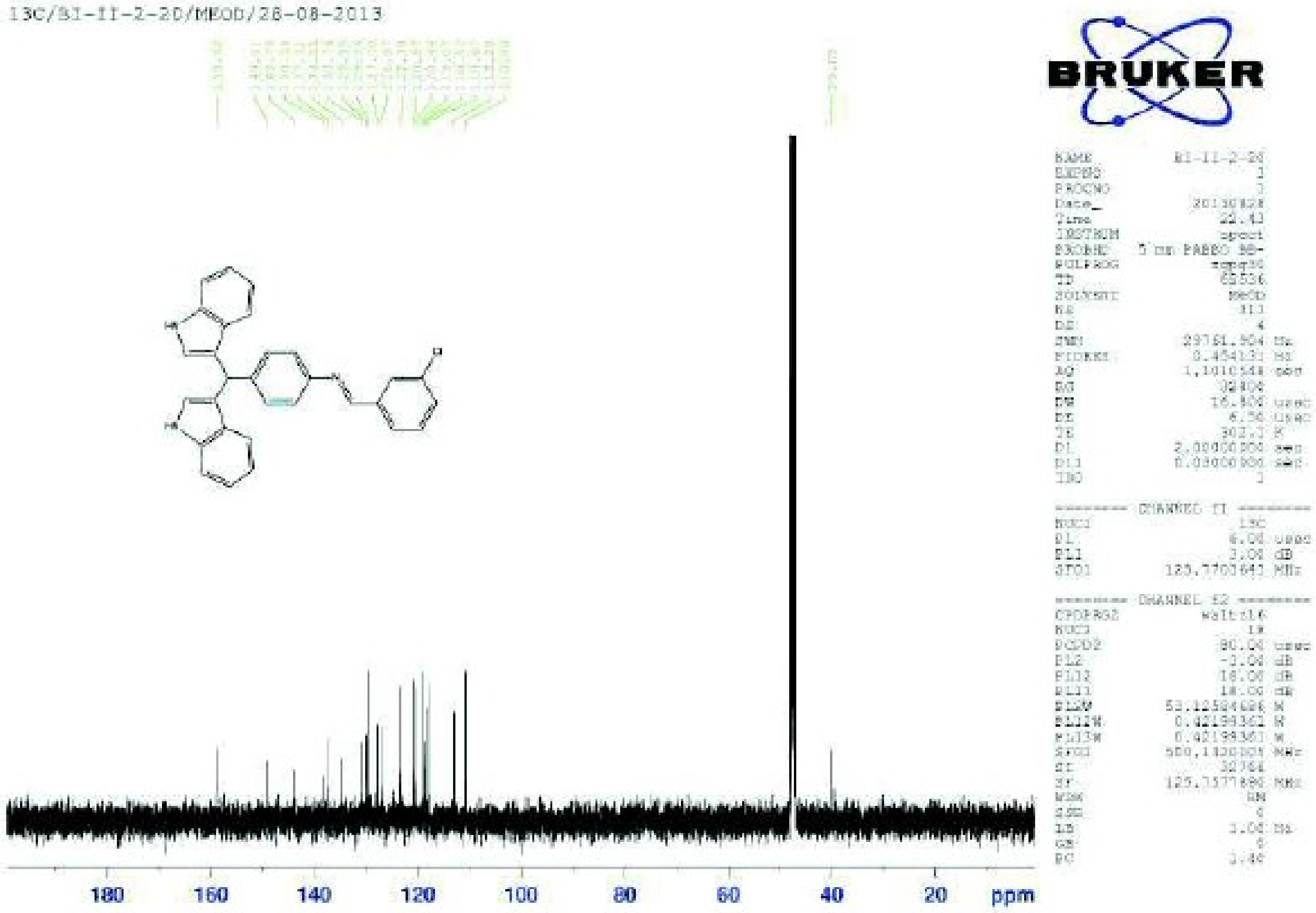

Compound 23

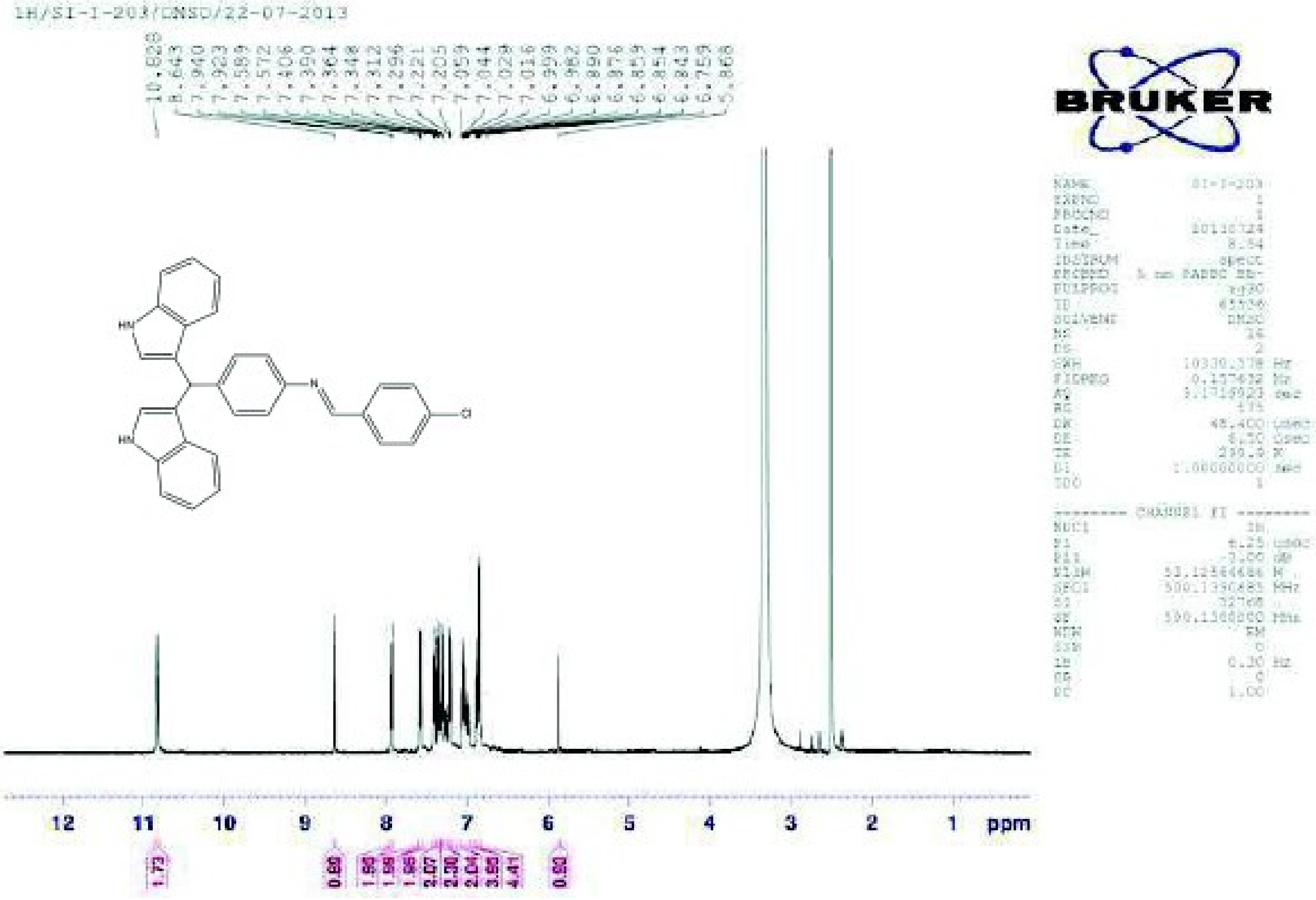

Compound 23

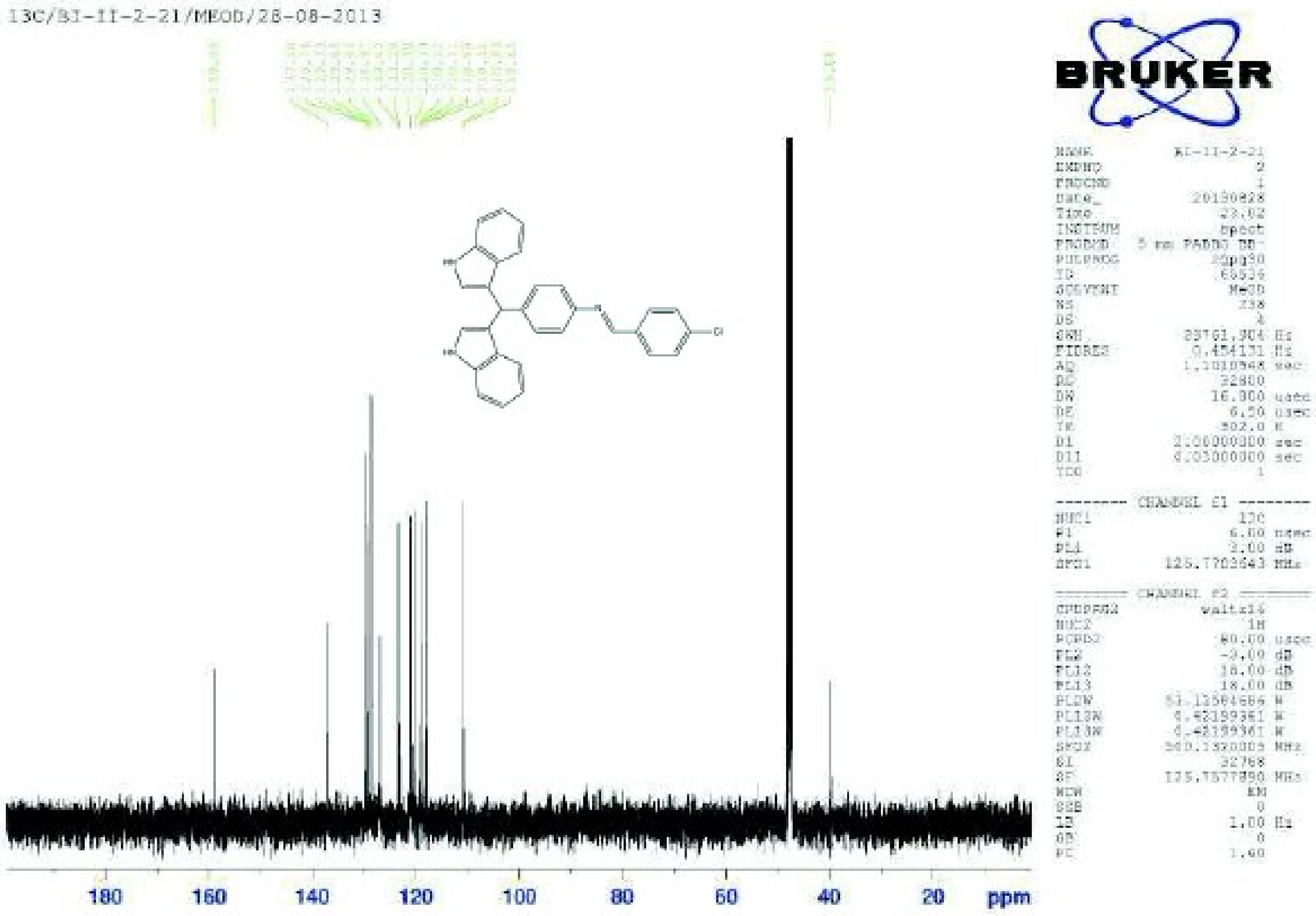
13C/BJ-II-2-21/MEOD/28-08-2013
Cl
180
160
140
120
100
80
60
40
20
ppm
CHANNEL f1
CHANNEL f2

Compound 24

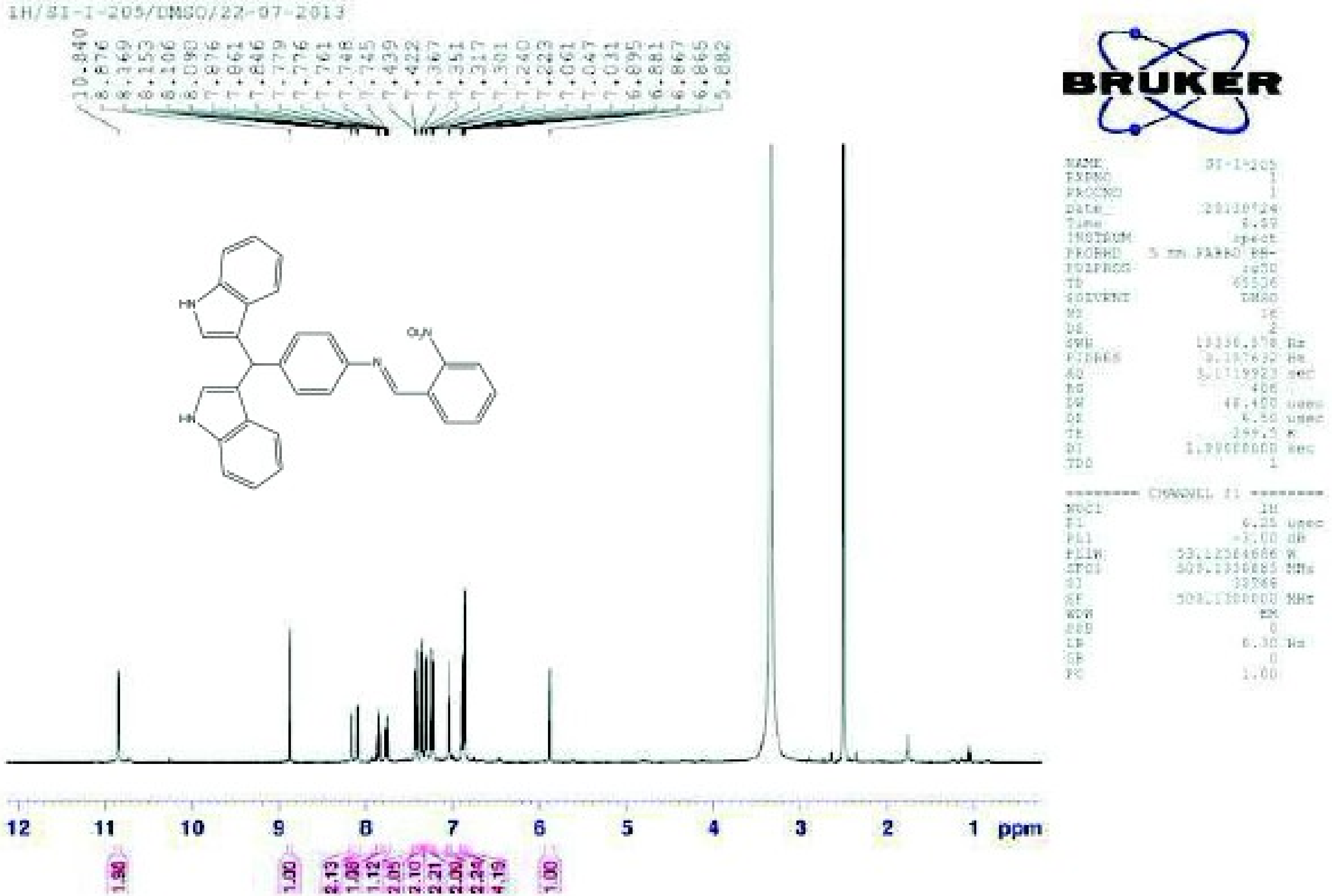

Compound 24

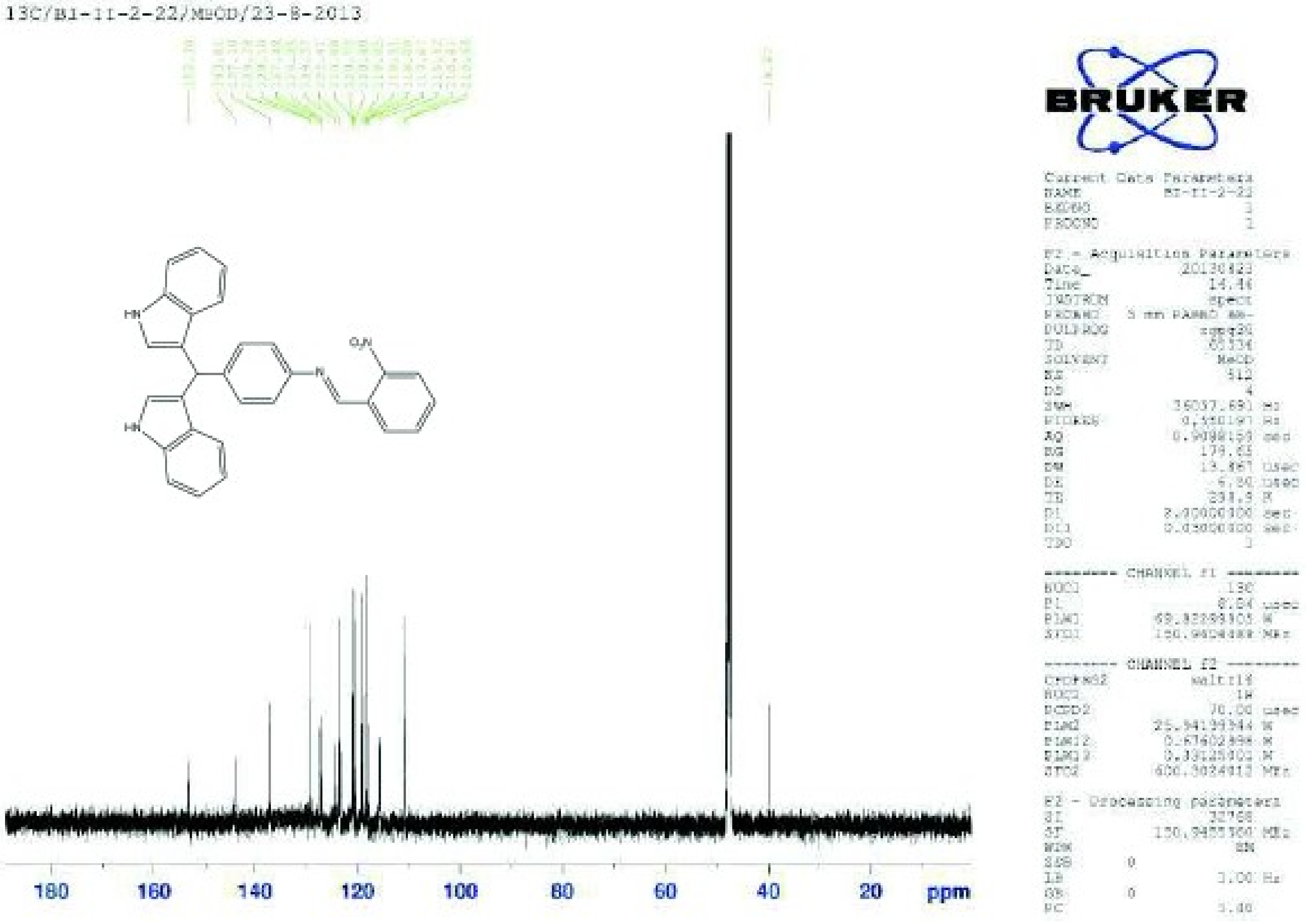
13C/BI-11-2-22/MeOD/23-8-2013
HN
HN
O_2N
N
180
160
140
120
100
80
60
40
20
ppm
Current Data Parameters
F2 - Acquisition Parameters
CHANNEL f1
CHANNEL f2
F2 - Processing parameters

Compound 25

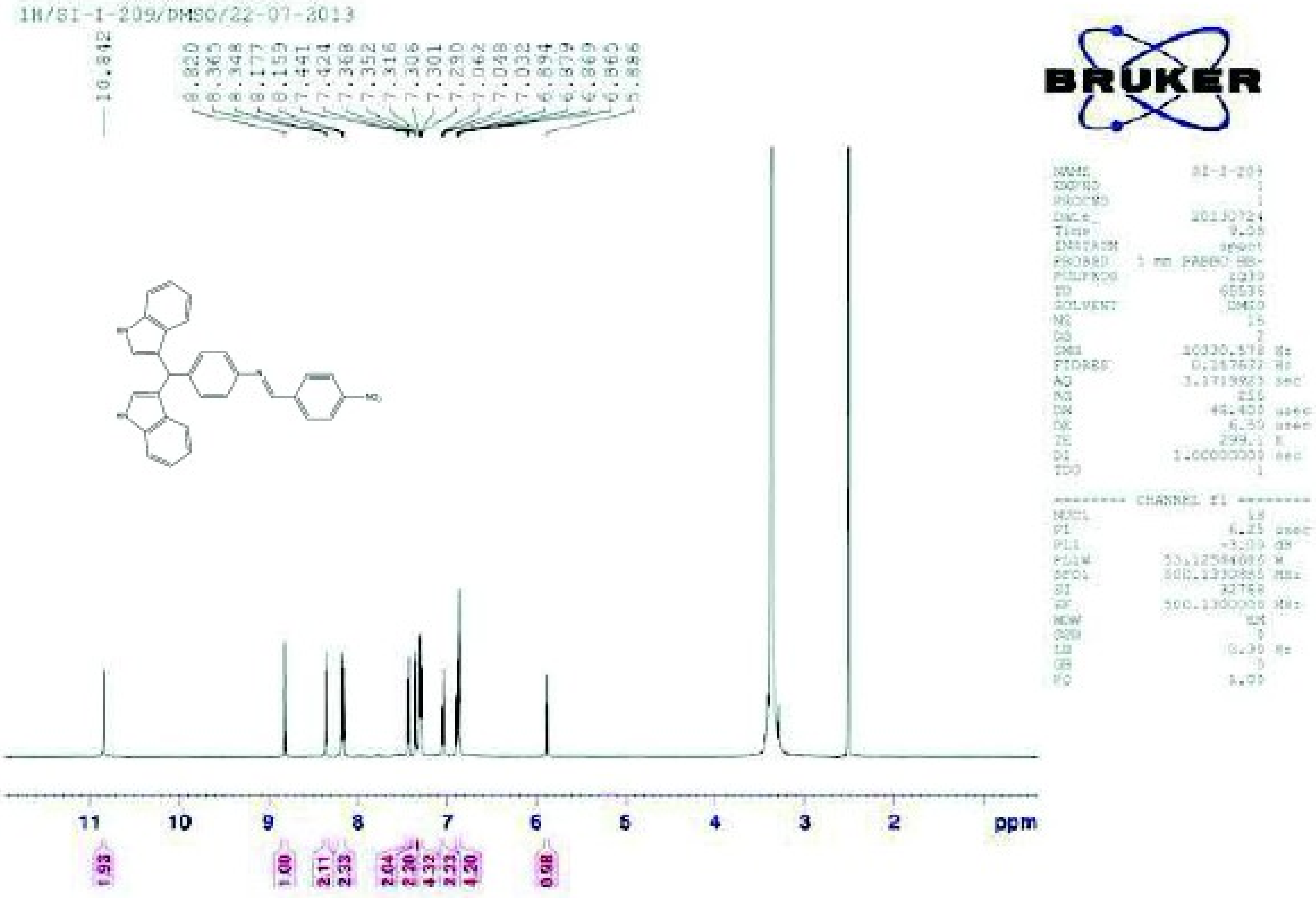
1H/SI-1-209/DMSO/22-07-2013
10.842
8.820
8.365
8.348
8.177
8.159
7.441
7.424
7.368
7.352
7.316
7.305
7.301
7.290
7.062
7.048
7.032
6.894
6.879
6.869
6.865
5.886
BRUKER
CHANNEL f1
11
10
9
8
7
6
5
4
3
2
ppm
1.98
1.00
2.11
2.33
2.04
2.20
4.32
2.23
4.20
0.98

Compound 25

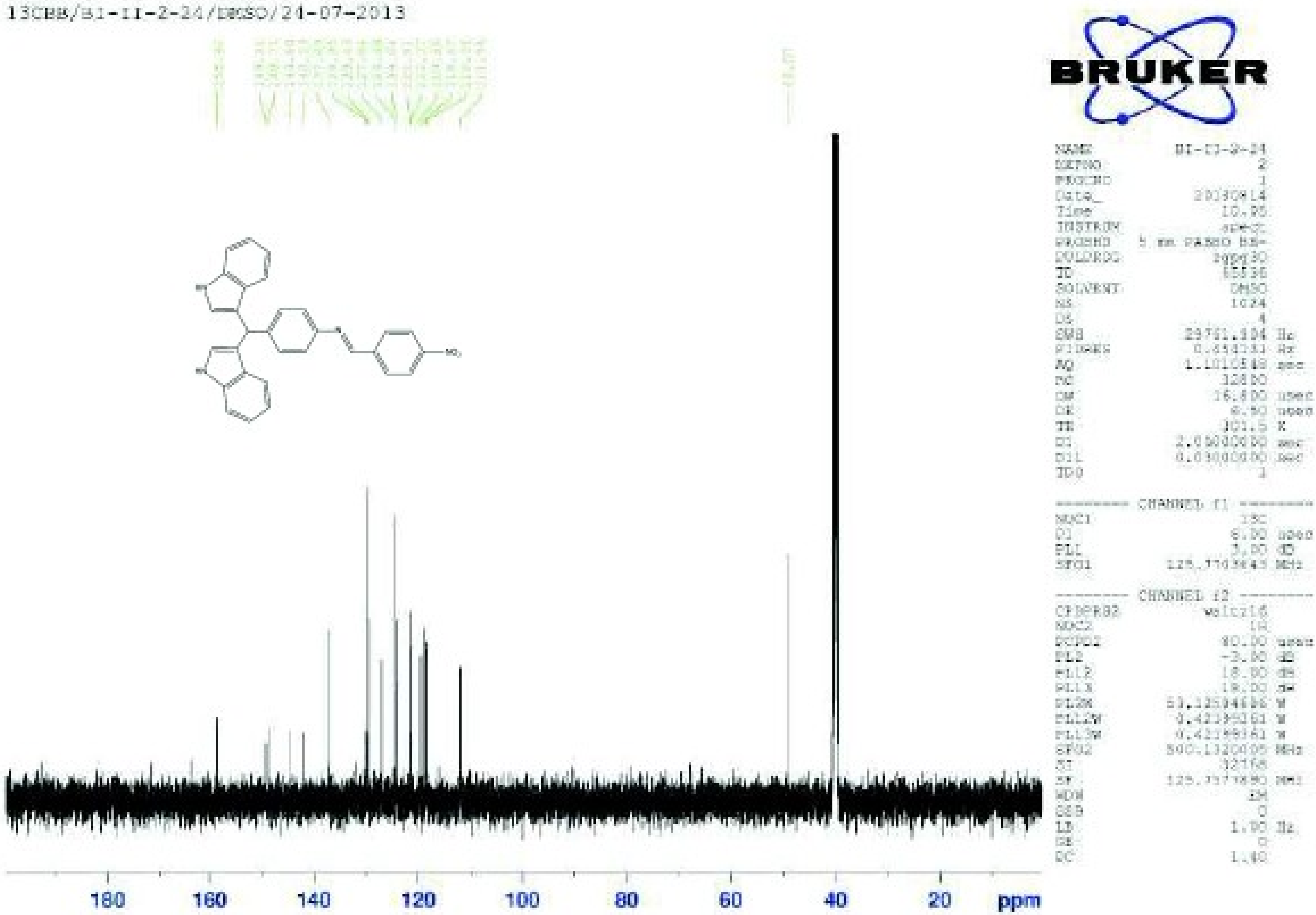
13CBB/BI-II-2-24/DMSO/24-07-2013
NAME
EXPNO
PROCNO
SOLVENT DMSO
NS 1024
CHANNEL f1
NUC1 13C
CHANNEL f2
NUC2 1H
180
160
140
120
100
80
60
40
20
ppm

Compound 26

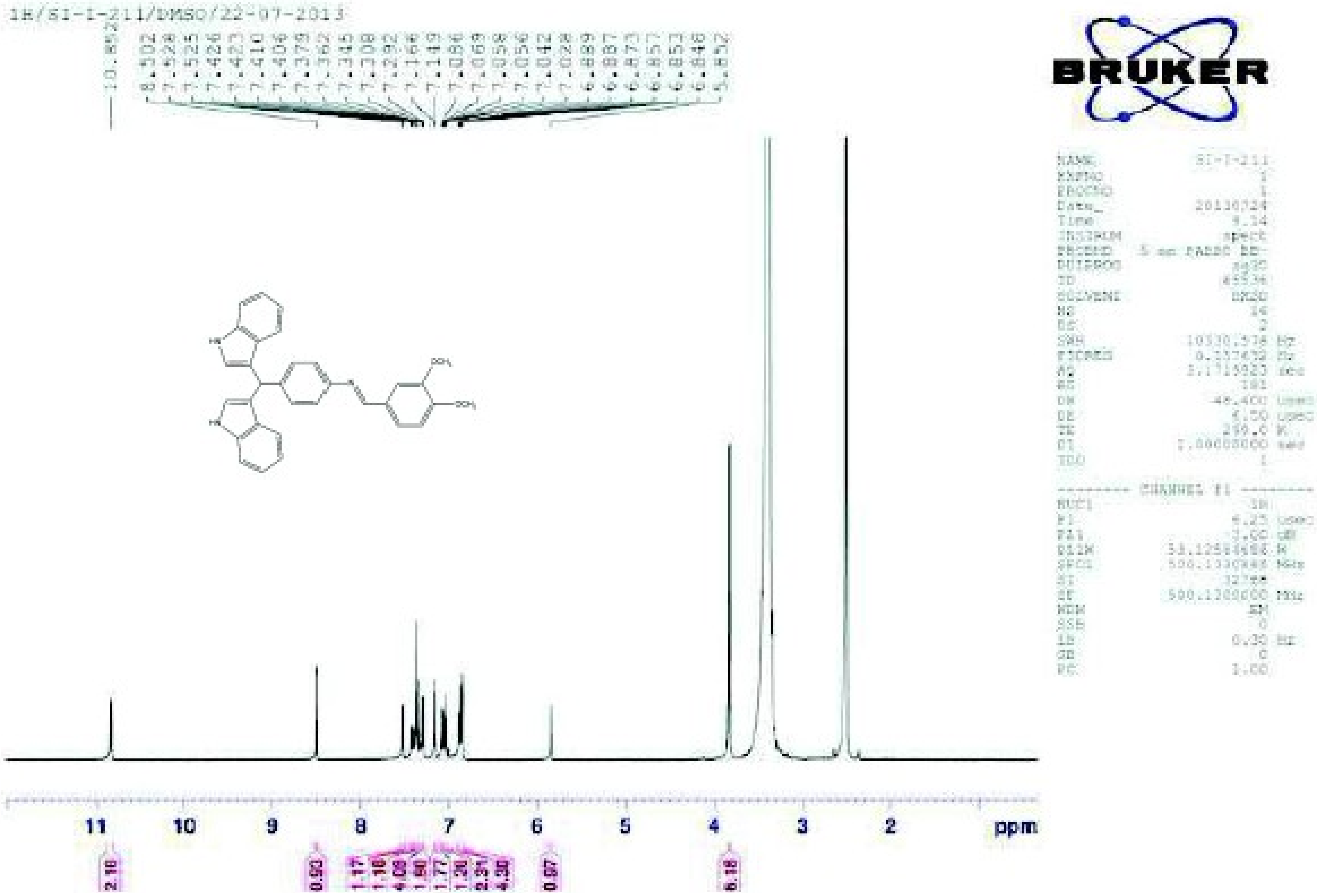

Compound 26

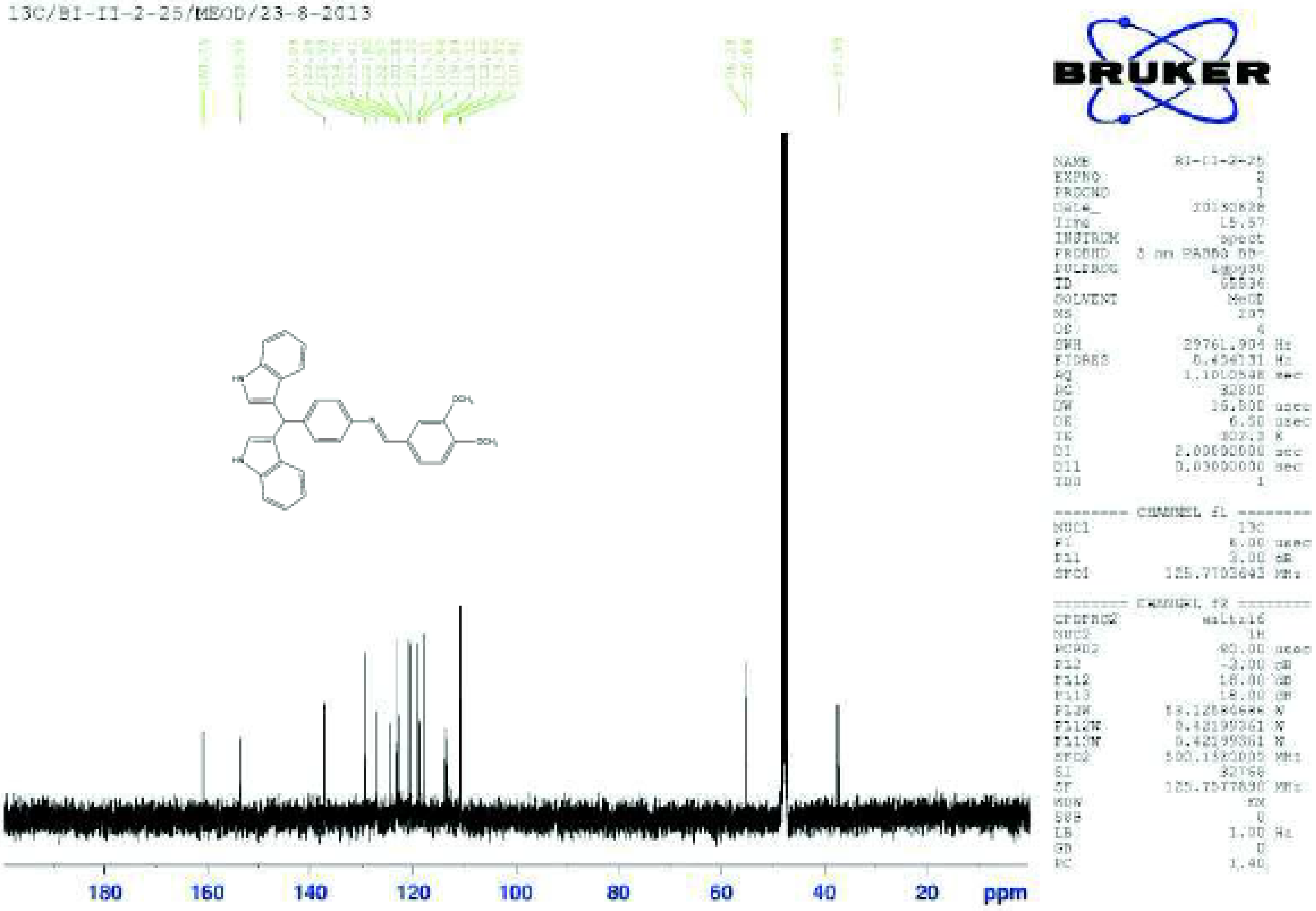
13C/BI-II-2-25/MEOD/23-8-2013
BRUKER
NAME
EXPNO 2
PROCNO 1
INSTRUM spect
SOLVENT MeOD
CHANNEL f1
NUC1 13C
CHANNEL f2
NUC2 1H
SI 32768
LB 1.00 Hz
180
160
140
120
100
80
60
40
20
ppm
